# CircHIPK2 Contributes Cell Growth in Intestinal Epithelial of Colitis and Colorectal Cancer through Promoting TAZ Translation

**DOI:** 10.1002/advs.202401588

**Published:** 2024-07-09

**Authors:** Xixi Zeng, Jielin Tang, Qian Zhang, Chenxing Wang, Ji Qi, Yusi Wei, Jiali Xu, Kaiyuan Yang, Zuolin Zhou, Hao Wu, Jiarong Luo, Yi Jiang, Zengqiang Song, Jinyu Wu, Jianmin Wu

**Affiliations:** ^1^ Key Laboratory of Laboratory Medicine Ministry of Education Institute of Genomic Medicine, School of Laboratory Medicine and Life Science Wenzhou Medical University Zhejiang 325035 China; ^2^ Zhejiang Key Laboratory of Intelligent Cancer Biomarker Discovery and Translation First Affiliated Hospital Wenzhou Medical University Zhejiang 325035 China; ^3^ The Joint Innovation Center for Health and Medicine Quzhou People's Hospital The Quzhou Affiliated Hospital of Wenzhou Medical University Zhejiang 324000 China; ^4^ Chemical Biology Research Center at School of Pharmaceutical Sciences Wenzhou Medical University Zhejiang 325035 China; ^5^ Department of Gastroenterology The Second Affiliated Hospital and Yuying Children of Wenzhou Medical University Zhejiang 325003 China; ^6^ Cixi Biomedical Research Institute Wenzhou Medical University Zhejiang 315302 China

**Keywords:** cell growth, circHIPK2, colorectal cancer, inflammatory bowel disease, TAZ

## Abstract

Colorectal cancer (CRC) and inflammatory bowel disease (IBD) are escalating global health concerns. Despite their distinct clinical presentations, both disorders share intricate genetic and molecular interactions. The Hippo signaling pathway plays a crucial role in regulating cell processes and is implicated in the pathogenesis of IBD and CRC. Circular RNAs (circRNAs) have gained attention for their roles in various diseases, including IBD and CRC. However, a comprehensive understanding of specific circRNAs involved in both IBD and CRC, and their functional roles is lacking. Here, it is found that circHIPK2 (hsa_circRNA_104507) is a bona fide circRNA consistently upregulated in both IBD and CRC suggesting its potential as a biomarker. Furthermore, silencing of circHIPK2 suppressed the growth of CRC cells in vitro and in vivo. Interestingly, decreased circHipk2 potentiated dextran sulfate sodium (DSS)‐induced colitis but alleviated colitis‐associated tumorigenesis. Most significantly, mechanistic investigations further unveil that circHIPK2, mediated by FUS, interacting with EIF4A3 to promote the translation of TAZ, ultimately increasing the transcription of downstream target genes CCN1 and CCN2. Taken together, circHIPK2 emerges as a key player in the shared mechanisms of IBD and CRC, modulating the Hippo signaling pathway. CircHIPK2‐EIF4A3 axis contributes to cell growth in intestinal epithelial of colitis and CRC by enhancing TAZ translation.

## Graphical abstract

Summary of the role of circHIPK2 in IBD and CRC. Circular RNA circHIPK2 emerges as a key player in the shared mechanisms of IBD and CRC. CircHIPK2 mediated by FUS, interacts with EIF4A3 to promote the translation of TAZ, ultimately increasing the transcription of downstream target genes CCN1 and CCN2, to contribute to cell growth in intestinal epithelial of colitis and CRC.

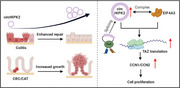

## 1 Introduction

Colorectal cancer (CRC) and inflammatory bowel disease (IBD), encompassing ulcerative colitis (UC) and Crohn's disease (CD), are complex gastrointestinal disorders with profound global health impacts.^[^ ^1^ ^]^ The prevalence of CRC and IBD has been significantly and alarmingly increasing on a global scale in recent years.^[^ ^2^ ^]^ CRC stands as the third most prevalent and second most fatal malignancy worldwide.^[^ ^1a^ ^]^ In 2020, an estimated 1.93 million new cases of CRC were diagnosed, with 0.9 million resulting in CRC‐related deaths.^[^ ^3^ ^]^ Extensive evidence, derived from a myriad of epidemiological investigations and experimental studies, consistently points that patients with IBD have a higher risk of developing CRC.^[^ ^1^, ^4^ ^]^ Despite these findings, our understanding of the precise mechanistic underpinnings driving colitis‐associated tumorigenesis remains limited.

While IBD and CRC have distinct clinical presentations and etiologies, they share a common feature‐complex interactions of genetic, environmental, and molecular factors.^[^ ^1^, ^5^ ^]^ The Hippo signaling pathway is a highly conserved kinase cascade that regulates cell proliferation, survival, mobility, stemness, and differentiation.^[^ ^6^ ^]^ Corruption of cell‐environment interplay leads to dysregulation of Hippo signaling including aberrant YAP and TAZ activation, which is instrumental in the development of IBD and CRC.^[^ ^6^ ^]^ As classical YAP/TAZ downstream targets, CCN1 and CCN2 also have been implicated in multiple diseases, including both IBD and CRC.^[^ ^7^ ^]^ Importantly, CCN1 expression is elevated in the colons of patients with CD or UC, as well as in mice with experimental colitis.^[^ ^8^ ^]^ CCN1 plays a role in promoting mucosal healing in murine colitis through IL‐6, further underscoring its relevance in the pathogenesis of IBD.^[^ ^7c^ ^]^ Moreover, CCN1 interacts with integrins to regulate intestinal stem cell (ISC) proliferation and differentiation.^[^ ^9^ ^]^ Therefore, amending aberrant Hippo signaling pathway such as YAP/TAZ activities and CCN axis has therapeutic potential for treating IBD and CRC.

Recent studies emphasize the significance of circular RNAs (circRNAs), an intriguing class of non‐coding RNAs that have attracted substantial attention for their potential roles in a wide range of diseases, including IBD and CRC.^[^ ^10^ ^]^ For example, circHIPK3 was reduced in the intestinal mucosa of IBD and sepsis patients. It promoted homeostasis of the intestinal epithelium.^[^ ^11^ ^]^ PRKAR2A‐derived circRNAs promoted the malignant transformation of colitis and predicted the prognosis of colitis‐associated CRC.^[^ ^12^ ^]^ Moreover, several circRNAs such as circRHOBTB3, circPTEN1, and circLECRC were shown to play tumor‐suppressive roles in CRC.^[^ ^13^ ^]^ However, despite these indications, we still lack a comprehensive understanding of the specific circRNAs involved in both IBD and CRC, and their functional roles have not been thoroughly explored.

To address this gap, our present study employed a systematic screening of the GEO database to identify differentially expressed circRNAs in both IBD and CRC. We found that circHIPK2 (hsa_circRNA_104507) was a bona fide circRNA upregulated in both IBD and CRC. Furthermore, silencing of circHIPK2 suppressed the growth of CRC cells in vitro and in vivo. Interestingly, decreased circHipk2 potentiated dextran sulfate sodium (DSS)‐induced colitis but alleviated colitis‐associated tumorigenesis. Most significantly, mechanistic investigations further unveiled that circHIPK2, mediated by FUS, bound to EIF4A3 to promote the translation of TAZ, ultimately upregulating the transcription of downstream target genes CCN1 and CCN2. Thus, modulating circHIPK2 expression holds potential as a therapeutic strategy for addressing both colitis and CRC.

## 2 Results

### 2.1 CircHIPK2 is a Bona Fide circRNA Upregulated in both IBD and CRC

**Figure 1:**
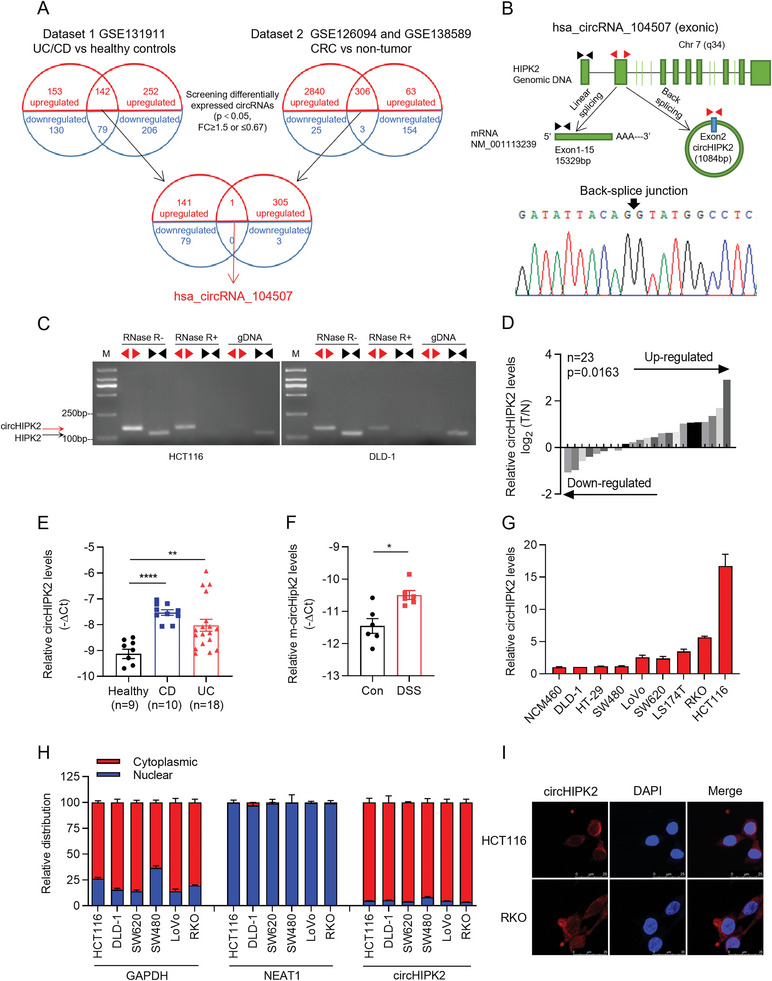
Identification of circHIPK2 as a circular RNA upregulated in both IBD and CRC. A) Schematic representation of the analysis strategy used to identify key circRNAs involved in colitis (IBD) and colorectal cancer (CRC) development. B) Genomic location of hsa_circRNA_104507 (circHIPK2) on chromosome 7, derived from Exon2 of the HIPK2 gene. RT‐qPCR followed by Sanger sequencing verified the cyclization site. C) Validation of circHIPK2's circular nature through RNase R digestion assay, highlighting its resistance to degradation compared to linear HIPK2. D,E) RT‐qPCR analysis of circHIPK2 expression in CRC (D) and IBD (E) colon tissues, showing significant upregulation compared to healthy controls. F) Elevation of circHipk2 levels in DSS‐induced acute colitis mice model (*n* = 6). G) circHIPK2 expression in a series of CRC cell lines and the normal colon mucosal epithelial cell line NCM460. H) Subcellular localization analysis of circHIPK2 using biochemical fractionation. GAPDH mRNA was used as a control for cytoplasmic transcripts, NEAT1 was used as a control for nuclear transcripts. I) FISH assays indicated that nearly 90% of circHIPK2 is localized in the cytoplasm of human CRC cells. Scale bar: 25 µm. (G) and (H) are presented as the mean ± SD; (E) and (F) are presented as the mean ± SEM. *p < 0.05, **p < 0.01, ****p < 0.0001 by Wilcoxon signed‐rank test in (D), or Mann‐Whitney *U* test in (E) and (F). DSS: dextran sulfate sodium.

To identify key circRNAs required for both colitis and CRC development and progression, we comprehensively analyzed the expression of circRNAs in two GEO datasets (**Figure** **1**A) obtained from: 1) Colon tissues of the patients with IBD (including UC and CD) and healthy controls (GSE131911: 221 dysregulated circRNAs, including 142 upregulated and 79 downregulated circRNAs; fold change ≥1.5 or ≤0.67, *P* < 0.05), and 2) CRC biopsies and normal colorectal tissues (GSE126094 and GSE138589: 309 dysregulated circRNAs, including 306 upregulated and 3 downregulated circRNAs; fold change ≥1.5 or ≤ 0.67, *P* < 0.05).^[^ ^14^ ^]^ We observed that a greater number of circRNAs were upregulated in pathological tissues (Table S1, Supporting Information). Among them, only hsa_circRNA_104507 was upregulated in both IBD and CRC samples, therefore, it was focused for further in‐depth study. hsa_circRNA_104507 (thereinafter referred to as circHIPK2) was located on chromosome 7 (139415730‐139416814) and derived from the Exon2 of HIPK2 gene (Figure 1B). The presence of a head‐to‐tail junction in the circHIPK2 was corroborated by Sanger sequencing (Figure 1B), consistent with the hsa_circ_0001756 annotation according to circbase.^[^ ^15^ ^]^ Further, RNase R digestion assay showed that circHIPK2 was more resistant to degradation than linear HIPK2, providing further evidence of its circular nature (Figure 1C). Utilizing our local cohort, RT‐qPCR assays also revealed a significant elevation in circHIPK2 levels within both CRC and IBD colon tissues, as compared to their respective healthy samples (Figure 1D,E). Furthermore, we constructed the DSS‐induced acute colitis mice model (Figure S1A–D, Supporting Information) and observed a significant increase in circHipk2 levels in DSS‐treated colonic tissues (Figure 1F). Additionally, RT‐qPCR survey of human CRC cell lines revealed that the circHIPK2 levels were substantially higher in the CRC cell lines as compared to the normal human colon mucosal epithelial cell line NCM460 (Figure 1G). Finally, subcellular localization analysis by biochemical fractionation and FISH assays revealed that almost 90% of circHIPK2 appeared in the cytoplasm of human CRC cells (Figure 1H,I). Altogether, these findings above suggest that circHIPK2 may be key roles involved in both colitis and CRC progression.

### 2.2 Decreased circHipk2 Potentiates DSS‐Induced Colitis but Alleviates Colitis‐Associated Tumorigenesis

**Figure 2:**
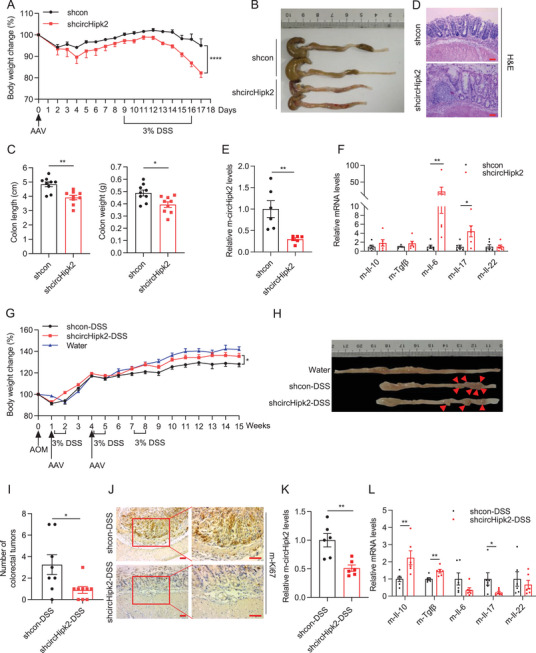
Effects of circHipk2 knockdown on colitis and colitis‐associated tumorigenesis. A–D) Schematic of experimental design: Seven‐week‐old male C57BL/6J mice received intrarectal administration of AAVs expressing shcircHipk2 (*n* = 9) or shcon (*n* = 9). One week later, acute colitis was induced by oral administration of 3% DSS in drinking water for 7 days, followed by 2 days of normal drinking water. All mice were euthanized on day 18. Relative body weight curves (A), Representative gross images of the colon of mice (B), measurement of colon length and weight (C), and representative HE‐stained colon sections showing inflammatory infiltration (D). E) Verification of circHipk2 knockdown efficiency using RT‐qPCR (*n* = 6). F) Cytokines expression (Il‐10, Tgfβ, Il‐17, IL‐6, and Il‐22) were measured by RT‐qPCR (*n* = 6). G–J) Schematic of the experimental setup for the colitis‐associated tumorigenesis model: Seven‐week‐old male C57BL/6J mice were intraperitoneally injected with 12.5 mg kg^−1^ AOM, followed by three cycles of 3% DSS treatment. AAVs containing shcircHipk2 (*n* = 9) or shcon (*n* = 8) were intrarectally administered to mice twice. All mice were euthanized 105 days after AOM injection. Control mice received normal drinking water and were not injected with adenovirus (*n* = 6). Relative body weight curves (G), representative images of observed colitis‐associated tumor in shcircHipk2 and shcon group (H), reduced development of colonic tumors in circHipk2 knockdown mice (I), and Ki67‐stained tissue sections in colon tissues (J). K) Quantification of circHipk2 knockdown efficiency (*n* = 6). L) Increased Il‐10 and Tgfβ levels and decreased Il‐17 levels in circHipk2 knockdown groups (*n* = 6). Scale bar: 50 µm. (A), (C), (E‐G), (I), and (K‐L) are presented as the mean ± SEM. *p < 0.05, **p < 0.01, ****p < 0.0001 by two‐way ANOVA in (A), or Mann‐Whitney *U* test in (C), (E‐G), (I), and (K‐L). The statistical significance of in (G) was based on the weights recorded at week 15 between shcircHipk2 and shcon group. AOM: azoxymethane; HE: hematoxylin and eosin.

To delve deeper into the biological function of circHipk2 in colitis, we conducted AAV‐mediated knockdown of circHipk2 in DSS‐induced acute colitis mice model (**Figure** **2**A). Upon DSS challenge, the mice with circHipk2 knockdown in colon exhibited more body weight loss and shorter colon length than that with controls (Figure 2A–C). Consistent with this, colonic inflammation was promoted, as evidenced by the more intestinal crypts damage as along with extensive inflammatory of cell infiltration in the colon tissues (Figure 2D; Figure S1E, Supporting Information). Knockdown efficiency of circHipk2 was checked by RT‐qPCR (Figure 2E). Since cytokines are important in inflammation and repair, we also measured their expression. As expected, the pro‐inflammatory cytokines Il‐6 and Il‐17 showed higher levels in the circHipk2 knockdown groups upon DSS challenge, whereas the anti‐inflammatory cytokines Il‐10, Tgfβ, and Il‐22 were similar levels (Figure 2F). In humans, patients with IBD develop colitis‐associated tumor by poorly understood mechanisms.^[^ ^4b^ ^]^ Subsequently, we investigated the effect of circHipk2 knockdown in a colitis‐associated tumor mice model induced by azoxymethane (AOM) plus DSS. Interestingly, we found that the mice with circHipk2 knockdown exhibited less body weight loss (Figure 2G) and developed fewer colonic tumors (Figure 2H,I) than control groups during the course of the challenge. Ki67 staining revealed that colon tissues from circHipk2 knockdown groups had fewer proliferative cells than those in the control group (Figure 2J). Furthermore, the knockdown efficiency of circHipk2 was determined (Figure 2K), and the levels of Il‐10 and Tgfβ increased, whereas the levels of Il‐17 decreased in circHipk2 knockdown groups (Figure 2L). Therefore, these findings suggest that decreased circHipk2 exerts bidirectional effects, in term of enhanced colitis and decreased colitis‐associated tumorigenesis.

### 2.3 Silencing of circHIPK2 Suppresses the Growth of CRC Cells In Vitro and In Vivo

**Figure 3:**
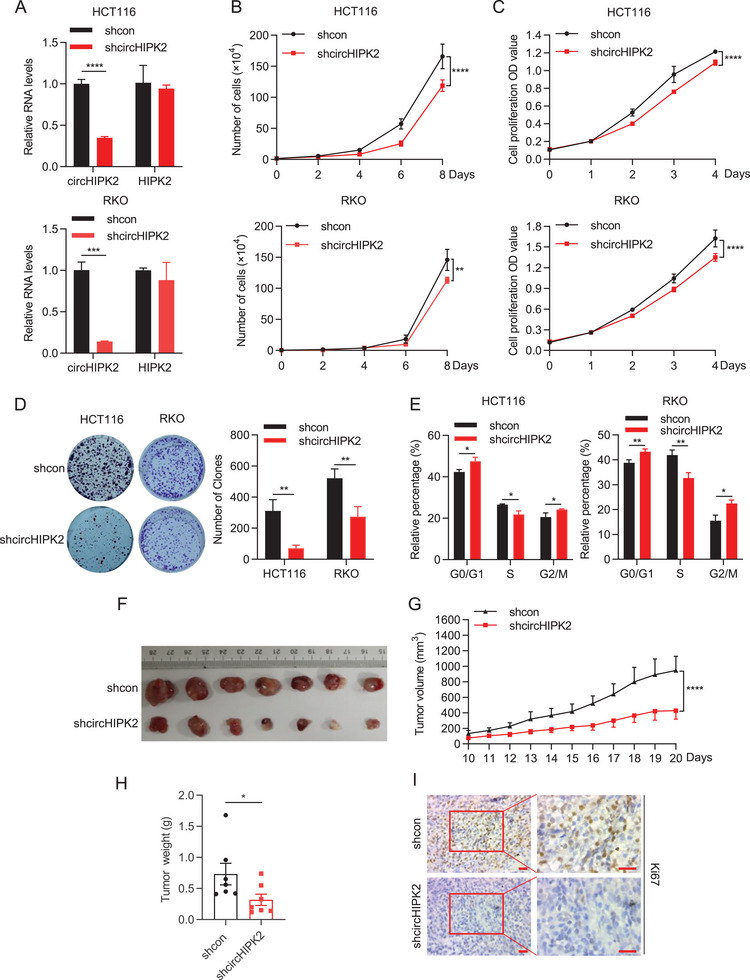
Silencing of circHIPK2 inhibits CRC cell growth in vitro and in vivo. A) Knockdown efficiency of circHIPK2 in stable shcircHIPK2‐HCT116 and ‐RKO CRC cell lines. B–D) Decreased cell proliferation observed in circHIPK2 knockdown cells compared to control, as demonstrated by cell counting (B), MTS assay (C), and colony formation assay (Left: representative images; right: quantification of colony numbers, D). E) Cell cycle was determined by flow cytometry. F–I) In vivo tumorigenesis study: shcircHIPK2 and shcon‐RKO cells injected subcutaneously into nude mice (*n* = 7). F) Autopsy images of tumors from xenografted nude mice. G) Measurement of tumor volumes at indicated time points. H) Assessment of the average weight of xenografted tumors. I) Representative images of Ki67‐stained tissue sections in xenografted tumors. Scale bar: 20 µm. (A‐E) are presented as the mean ± SD; (G‐H) are presented as the mean ± SEM. *p < 0.05, **p < 0.01, ***p < 0.001, ****p < 0.0001 by Student's *t*‐test in (A), (D) and (E), two‐way ANOVA in (B‐C) and (G), or Mann‐Whitney *U* test in (H).

To investigate the effects of circHIPK2 on the proliferation of CRC cells, two strategies were employed including transient transfection and generation of stable CRC cell lines (HCT116 and RKO), and knockdown efficiency was determined by RT‐qPCR (**Figure** **3**A; Figure S2A, Supporting Information). As anticipated, knockdown of circHIPK2 had notable inhibitory effects on cell proliferation, as assessed by cell numbers, MTS and colony formation assays in both cell lines (Figure 3B–D; Figure S2B,C, Supporting Information). Additionally, in a cell cycle assay, we observed that knockdown of circHIPK2 significantly arrested the cell cycle at the G1‐S transition (Figure 3E; Figure S2D, Supporting Information). To further explore the influence of circHIPK2 on tumor formation in vivo, we conducted an in‐vivo tumorigenesis study by injecting shcircHIPK2‐RKO cells subcutaneously into nude mice. After a period of 20 days post‐inoculation, the mice from both the shcircHIPK2‐RKO and control shcon groups were euthanized. Our observations revealed a notable reduction in both average tumor volumes and weights within the shcircHIPK2‐RKO groups (Figure 3F–H). Immunohistochemical analysis of tumors from the shcircHIPK2 group demonstrated a significant decrease in Ki67 expression (Figure 3I). Collectively, these findings provide compelling evidence that the suppression of circHIPK2 exerts a substantial inhibitory effect on CRC cells in vitro and in vivo context.

### 2.4 TAZ is One of Core Downstream Targets of circHIPK2

**Figure 4:**
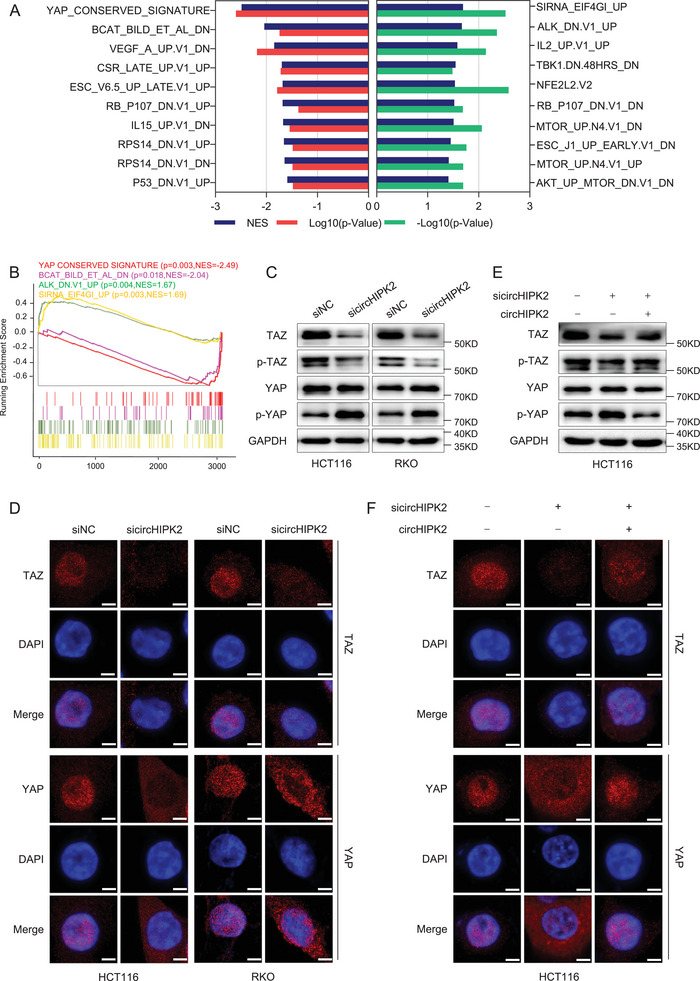
TAZ as a central downstream target of circHIPK2. A) The 20 most enriched oncogenic signature gene sets (ten positive and ten negative each) in circHIPK2 knockdown cells according to GSEA analysis were ranked based on normalized enrichment score (NES). B) Negative enrichment score curves for the Hippo/YAP pathway, indicating downregulation following circHIPK2 knockdown. C) The protein levels of TAZ, phosphorylated TAZ (Ser89), YAP, and phosphorylated YAP (Ser127) in circHIPK2 knockdown cells were determined by western blotting. D) Immunofluorescence staining of TAZ and YAP proteins in CRC cells upon circHIPK2 knockdown. E) Rescue of circHIPK2 expression restored the levels of TAZ, phosphorylated TAZ (Ser89), and phosphorylated YAP (Ser127). F) Subcellular localization of TAZ/YAP is restored following the rescue of circHIPK2 expression. Scale bar: 10 µm.

To delve into the molecular mechanisms governing circHIPK2's impact on cell proliferation, we conducted transcriptomic RNA sequencing on HCT116 cells subjected to si‐circHIPK2 treatment. A total of 492 genes showed significant upregulation, while 303 genes exhibited significant downregulation (fold change ≥ 2 or ≤ 0.5, *P* < 0.05) in circHIPK2 knockdown HCT116 cells compared to the control cells (Table S2, Supporting Information). Subsequently, gene set enrichment analysis (GSEA) based on 189 oncogenic signature gene sets (C6 collection) was performed. Interestingly, the YAP conserved signature representing Hippo/YAP pathway was the most enriched among the genes dysregulated by circHIPK2 knockdown (**Figure** **4**A; Table S3, Supporting Information), and negative enrichment score curves for Hippo/YAP pathway were depicted in Figure 4B. These findings may have contributed to the significant changes observed in biological processes as mentioned earlier. The Hippo pathway has been shown to influence a broad spectrum of biological processes, particularly in cell growth.^[^ ^6^ ^]^ And YAP/TAZ, as core components of the Hippo signaling pathway, play crucial roles in both intestinal regeneration and the proliferation of cancer cells.^[^ ^7b^ ^]^ To this end, we assessed both mRNA and protein levels of YAP and TAZ in the circHIPK2 knockdown cells. We found that circHIPK2 knockdown decreased the levels of total TAZ protein and phosphorylated TAZ (Ser89), and increased the level of phosphorylated YAP (Ser127), but had almost no effect on the mRNA levels of both TAZ and YAP (Figure 4C; Figure S3A–C, Supporting Information). Consistent with human cell lines, the protein levels of m‐Taz were significantly suppressed in circHipk2‐knockdown MC38 cells (Figure S4A–D, Supporting Information). Moreover, the results of TAZ changes induced by circHIPK2 were validated in circHIPK2‐knockdown xenografted tumors from nude mice and circHipk2‐knockdown colon tissues from AOM/DSS induced mice model (Figure S4E,F, Supporting Information). Similarly, immunofluorescence staining demonstrated a decrease in TAZ levels in both the nucleus and cytoplasm, whereas YAP translocation from nucleus to cytoplasm (Figure 4D). Furthermore, rescuing circHIPK2 expression completely restored the levels of total TAZ protein and phosphorylated TAZ (Ser89), and phosphorylated YAP (Ser127) (Figure 4E; Figure S3D, Supporting Information). Likewise, the subcellular localization of TAZ/YAP also was reinstated (Figure 4F; Figure S3E, Supporting Information). To further explore the specific role of circHIPK2, CHX or MG132 were used to inhibit the translation or degradation process of TAZ. We found that TAZ levels were similar between circHIPK2 knockdown cells and control cells after treatment with CHX. However, TAZ levels still decreased in circHIPK2 knockdown cells after treatment with MG132 (Figure S3F, Supporting Information). Collectively, these findings unveil circHIPK2's role in regulating the translational process of TAZ, emphasizing its significant involvement in the modulation of Hippo signaling.

### 2.5 CircHIPK2 Binds to EIF4A3 and Promotes the Translation of TAZ

Several previous studies have shown that circRNA exerts biological functions through its interactions with partner proteins.^[^ ^10^, ^16^ ^]^ To validate the direct binding protein of circHIPK2, we conducted a circRNA pull‐down assay using the MS2‐binding protein (MS2bp), which is known to specifically interact with RNAs containing MS2‐binding sequences (MS2bs). This approach allowed us to capture endogenous proteins associated with circHIPK2 (Figure S5A, Supporting Information). First, the plasmids containing the circHIPK2 transcript fused with or without MS2bs elements (referred to as pMS2bs‐circHIPK2 and pcircHIPK2) were separately co‐transfected into HCT116 cells along with a construct encoding MS2bp‐Flag, respectively. Subsequently, immunoprecipitation was conducted using an anti‐Flag antibody, with IgG utilized as a negative control. As depicted in Figure S5B,C (Supporting Information), circHIPK2 was validated as being specifically enriched in pMS2bs‐circHIPK2 pull‐down samples. Then, the label‐free mass spectrometric analysis was performed, and 59 proteins exhibited significant enrichment in pull‐down samples from pMS2bs‐circHIPK2 group (Table S4, Supporting Information).

**Figure 5:**
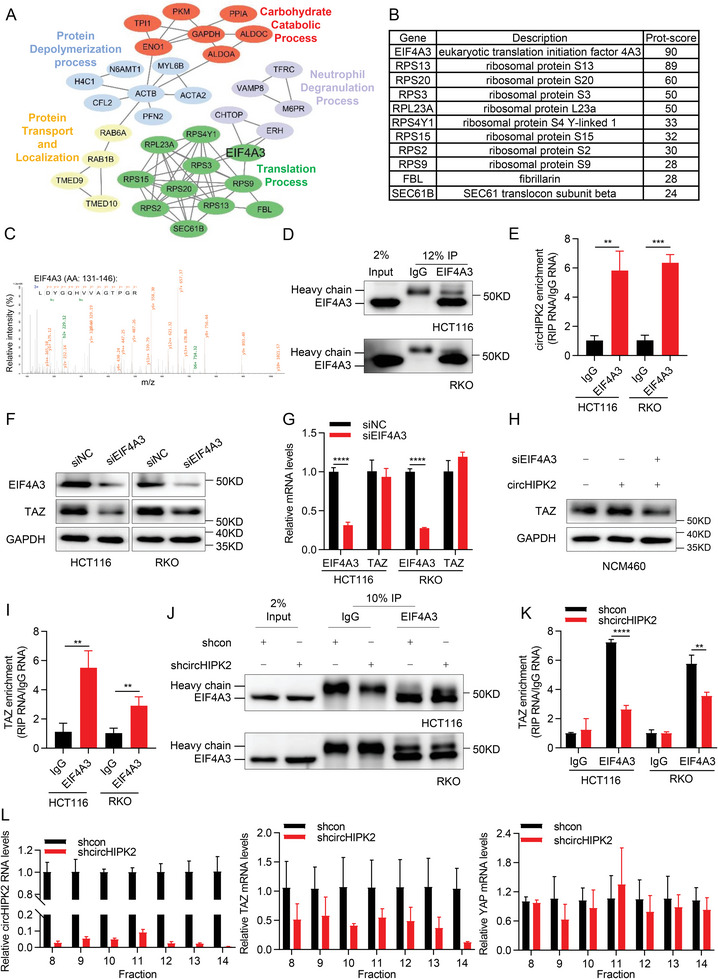
CircHIPK2 interacts with EIF4A3 and promotes TAZ translation. A) Reactome analysis conducted using STRING PPI plugin revealed that the circHIPK2 pull‐down proteins were involved in a multitude of vital biological processes. B) EIF4A3 was highlighted as the most enriched protein in the cluster of translation processes. C) Detection of EIF4A3 in the precipitate obtained from RNA pull‐down by LC‐MS/MS. D) Validation of EIF4A3‐immunoprecipitation (IP) efficiency by western blotting. E) Verification of endogenous interactions between circHIPK2 and EIF4A3 through RNA immunoprecipitation (RIP) assays. F) EIF4A3 depletion leads to a reduction in TAZ protein expression. G) The mRNA levels of TAZ were measured following EIF4A3 knockdown. H) EIF4A3 knockdown inhibited circHIPK2‐induced increases in the TAZ protein level in NCM460 cells. I) RIP analysis demonstrated efficient enrichment of TAZ by EIF4A3. J–K) The effect of circHIPK2 knockdown on the binding of EIF4A3 to TAZ was assessed by RIP assay. EIF4A3‐IP efficiency (J) and TAZ enrichment efficiency (K) were shown. L) Analysis of TAZ and YAP enrichment in polyribosomes after circHIPK2 knockdown using a sucrose gradient ribosome separation assay. Fourteen polysome fractions were collected from top to bottom of 5 to 50% sucrose gradient by ultracentrifuge. circHIPK2 (left), TAZ (middle), and YAP (right) RNA levels in eight to fourteen fraction were determined by RT‐qPCR. (E), (G), (I), (K), and (L) are presented as the mean ± SD. **p < 0.01, ***p < 0.001, ****p < 0.0001 by Student's *t*‐test in (E), (G), (I), and (K).

Gene ontology (GO) analysis revealed that the top enriched biological processes included co‐translational protein processes and nuclear‐transcribed mRNA catabolic processes (Figure S5D, Supporting Information). The Reactome analysis integrated into STRING PPI also indicated that one of the top five enriched pathways included the translation process, with the maximum number of physical interactional events involved (**Figure** **5**A). In the cluster of translation processes, eukaryotic translation initiation factor 4A3 (EIF4A3) stood out as the most significantly enriched protein (Figure 5B,C; Figure S5E, Supporting Information). This observation is particularly noteworthy because EIF4A3 is a core component of the Exon Junction Complex (EJC), actively participating in mRNA post‐transcriptional regulation.^[^ ^17^ ^]^ Consequently, it is plausible to consider EIF4A3 as a potential binding partner for circHIPK2. Endogenous interactions between circHIPK2 and EIF4A3 were confirmed through RNA immunoprecipitation (RIP) assays (Figure 5D,E). Therefore, these data demonstrate that circHIPK2 and EIF4A3 can indeed form a complex. Previous studies have shown that EIF4A3 can induce the biogenesis of circRNAs.^[^ ^18^ ^]^ Therefore, we investigated the expression of circHIPK2 after knocking down EIF4A3 in HCT116 and RKO cells. We found that depletion of EIF4A3 had no discernible effect on circHIPK2 expression (Figure S5F, Supporting Information). These findings led us to hypothesize that suppression of EIF4A3 would induce regulatory changes similar to those observed with circHIPK2 silencing. Consistent with the effects of circHIPK2 silencing, depletion of EIF4A3 resulted in the inhibition of TAZ protein expression, while no discernible effect on TAZ mRNA levels (Figure 5F,G). Moreover, EIF4A3 knockdown reduced circHIPK2‐induced increases in the TAZ protein levels in NCM460 cells (Figure 5H), which suggesting that the regulation of TAZ translation by circHIPK2 is partially dependent on EIF4A3. Furthermore, the RIP analysis substantiated the efficient enrichment of TAZ by EIF4A3 (Figure 5I), and the enrichment of TAZ mRNA with EIF4A3 protein diminished upon circHIPK2 knockdown (Figure 5J,K). These findings proved that circHIPK2 enhanced TAZ protein levels through partially dependent on EIF4A3. Combining our observation that a multitude of ribosomal proteins were also pulled down by circHIPK2, as illustrated in Figure 5A,B, we investigated the impact of circHIPK2 on TAZ translation process. Therefore, a sucrose gradient fractionation assay was conducted, followed by RT‐qPCR analysis of TAZ mRNA levels in eight to fourteen isolated polysome fraction. As anticipated, the knockdown of circHIPK2 led to a significant decrease in TAZ mRNA within polysome association, while it had no impact on the polysome association of YAP mRNA (Figure 5L). Thus, circHIPK2 appears to specifically enhance the translational efficiency of TAZ mRNA without affecting the translation of other mRNA molecules.

### 2.6 CircHIPK2 Modulates the Hippo Signaling Pathway During Colitis and CRC

**Figure 6:**
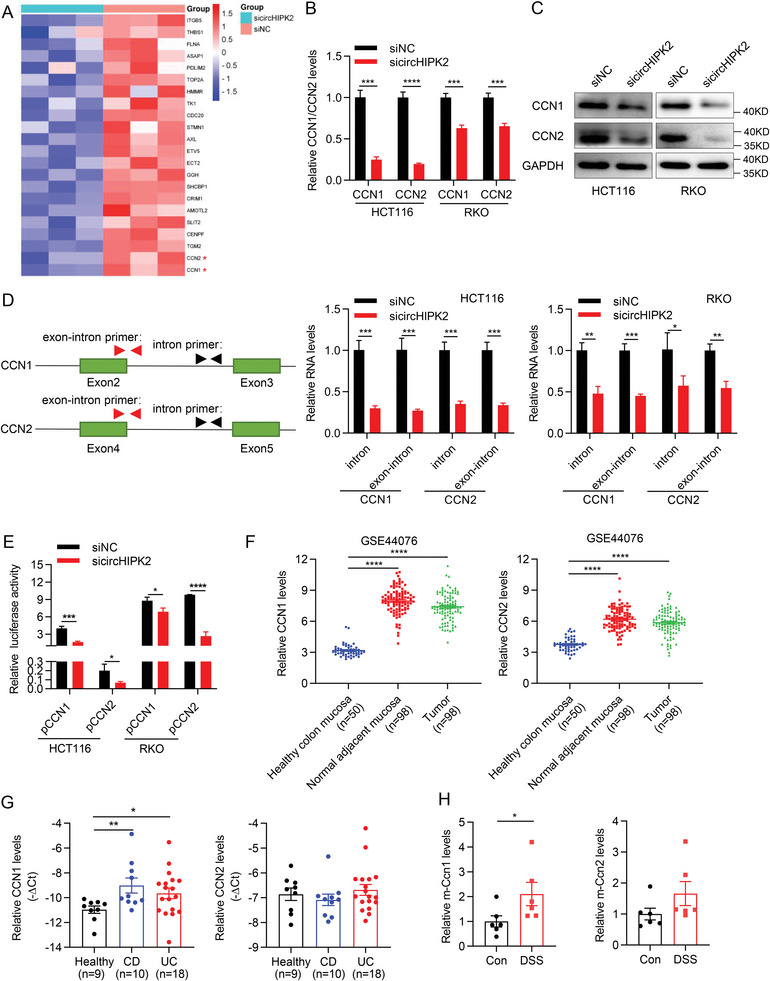
CircHIPK2 modulates the Hippo signaling pathway during colitis and CRC. A) Heatmap depicting the 22 significantly downregulated genes of YAP conserved signature upon circHIPK2 knockdown. B,C) Validation of circHIPK2 knockdown's effect on CCN1 and CCN2 mRNA (B) and protein (C) levels through RT‐qPCR and western blotting. D) RT‐qPCR analysis of CCN1 and CCN2 precursor RNA levels following circHIPK2 knockdown. Schematic diagram of primers for detecting gene precursor levels (left), and the levels of CCN1 and CCN2 precursor RNA (middle and right). E) The promoter regions (about 2‐kb region upstream of CCN1 and CCN2) were inserted into luciferase reporter constructs (named pCCN1 and pCCN2). Luciferase reporter activity was shown in CRC cells upon circHIPK2 knockdown. F) Scatter plot showing that the expression of CCN1 and CCN2 was significantly upregulated in tumor tissues and normal adjacent mucosa compared to healthy colon mucosa tissues (GSE44076). G) RT‐qPCR analysis of CCN1 (left) and CCN2 (right) expression in IBD colon tissues. H) Elevation of Ccn1 and Ccn2 levels in DSS‐induced acute colitis mice model (*n* = 6). (B), (D) and (E) are presented as the mean ± SD; (F‐H) are presented as the mean ± SEM. *p < 0.05, **p < 0.01, ***p < 0.001, ****p < 0.0001 by Student's *t*‐test in (B), (D), and (E), or Mann‐Whitney *U* test in (F‐H).

The Hippo signaling pathway plays a pivotal role in both IBD and CRC.^[^ ^6^ ^]^ The findings that circHIPK2 enhances the translation levels of TAZ as mentioned above indicate that circHIPK2 may function on colitis and colitis‐associated tumorigenesis through modulate Hippo signaling. To this end, we conducted an analysis of the gene expression of YAP conserved signature. Our findings revealed that 22 of 57 genes in Hippo pathway exhibited a significant downregulation upon circHIPK2 knockdown. Among them, CCN1 and CCN2, as the classical YAP/TAZ target genes, were two most significantly decreased genes (**Figure** **6**A; Table S5, Supporting Information). CCN1 and CCN2 have been implicated in both IBD and CRC, so we first examined their effects on cell proliferation. As anticipated, treatment with CCN1/2 recombinant protein or overexpression of CCN1/2 significantly promoted cell proliferation, as assessed by cell count, MTS assay, and colony formation assays in both HCT116 and RKO cell lines (Figure S6A–D, Supporting Information). Notably, the expression levels of CCN1 and CCN2 can serve as indicators of YAP/TAZ activity.^[^ ^19^ ^]^ Therefore, the mRNA and protein levels of these two representative genes following circHIPK2 knockdown were further validated through RT‐qPCR and western blotting. As expected, circHIPK2 knockdown led to a notable reduction in both CCN1 and CCN2 mRNA and protein levels (Figure 6B,C). Furthermore, we designed the primers for CCN1 and CCN2 pre‐mRNA and quantified their levels using RT‐qPCR (Figure 6D, left). Notably, the knockdown of circHIPK2 resulted in a suppression of CCN1 and CCN2 precursor RNA levels (Figure 6D, middle, right). To further investigate the influence of circHIPK2 on transcriptional activities of YAP/TAZ, the promoter regions of 2‐kb length upstream of CCN1 and CCN2 were used to construct luciferase reporter plasmids (as named pCCN1 and pCCN2), respectively. Co‐transfection experiments revealed that the depletion of circHIPK2 led to a suppression of the luciferase activities of both pCCN1 and pCCN2 (Figure 6E), suggesting that circHIPK2 enhanced transcriptional activities of YAP/TAZ. Analysis of publicly available datasets demonstrated an upregulation of CCN1 and CCN2 expression in tumor tissues and normal adjacent mucosa compared to healthy colon mucosa tissues (Figure 6F). Furthermore, we observed an increased expression of CCN1 in the colonic tissues of patients with IBD compared to healthy tissues, although there was no significant change in the expression of CCN2 (Figure 6G). Additionally, the expression of Ccn1 was also significantly upregulated in DSS‐treated colonic tissues (Figure 6H; Figure S1, Supporting Information). Collectively, these findings provide substantial evidence on the impact of circHIPK2 on Hippo signaling pathway through enhancing transcriptional activities of YAP/TAZ in colitis and CRC.

### 2.7 FUS Promotes the Expression of circHIPK2

**Figure 7:**
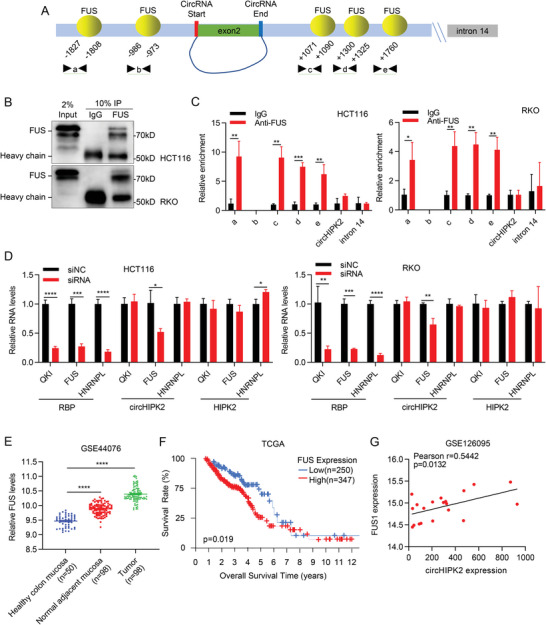
FUS Promotes circHIPK2 Expression. A) Prediction of FUS binding sites in the flanking sequences of circHIPK2 pre‐mRNA by RBPmap database. Five positions (a‐e) in circHIPK2 pre‐mRNA were selected to design qPCR primers. B,C) Validation of FUS binding to circHIPK2 pre‐mRNA through a RIP assay using FUS antibody. FUS‐IP efficiency by western blotting was shown (B), and significant enrichment of RNA fragments in specific binding sites (a, c, d, and e) was observed in FUS‐RIP samples (C). D) The relative levels of circHIPK2 and HIPK2 were determined upon RBPs (including QKI, FUS, and HNRNPL) knockdown by RT‐qPCR. E) Scatter plot showing that the expression of FUS was significantly upregulated in tumor tissues and normal adjacent mucosa (GSE44076). F) Kaplan‐Meier survival curve analysis revealing that CRC patients with high FUS expression have a significantly shorter overall survival time than those with low FUS expression. G) A significant positive correlation between FUS and circHIPK2 expression in CRC tissues from GSE126095 was shown. (C) and (D) are presented as the mean ± SD; (E) are presented as the mean ± SEM. *p < 0.05, **p < 0.01, ***p < 0.001, ****p < 0.0001 by Student's *t*‐test in (C) and (D), Mann‐Whitney *U* test in (E) or Pearson's correlation test (G).

To unravel the mechanism responsible for the upregulation of circHIPK2 in IBD and CRC cells, we performed a comprehensive bioinformatics analysis using online databases, including circAtlas (http://circatlas.biols.ac.cn/), RBPmap (http://rbpmap.technion.ac.il/), and RBPs in ref. [16] Our investigation identified several RBPs capable of binding to flanking regions of the circHIPK2 mRNA transcript (circHIPK2 pre‐mRNA). Remarkably, among these RBPs, FUS was consistently identified in all three databases (Figure S7A and Table S6, Supporting Information). Further analysis revealed twelve putative FUS binding sites in the upstream and downstream regions of the circHIPK2 pre‐mRNA. Due to the overlap of the several binding sites, we named the putative binding sequences in the upstream region separately as a and b, and the putative binding sequences in the downstream region separately as c, d, and e (**Figure** **7**A; Figure S7B, Supporting Information). To validate the binding interaction between FUS and circHIPK2 pre‐mRNA, we performed a RIP assay employing an anti‐FUS antibody. Our findings demonstrated a significant enrichment of RNA fragments of a, c, d, and e (with no PCR product observed in b) in FUS‐RIP samples compared to IgG‐RIP samples, but circHIPK2 and a random sequence within intron 14 did not exhibit this enrichment (Figure 7B,C). These data indicated that FUS does indeed bind to circHIPK2 pre‐mRNA through the putative binding sites. Furthermore, RT‐qPCR assay showed that the expression of circHIPK2 was notably reduced only by knockdown of FUS, not other‐ RBPs, such as QKI and HNRNPL, while the mRNA level of HIPK2 showed no significant change (Figure 7D; Figure S7C‐E, Supporting Information). Moreover, we found that there was no difference in the circHIPK2 decay ratio between the FUS‐knockdown group and the control group upon actinomycin D treatment, indicating that FUS does not affect the stability of circHIPK2 (Figure S7F, Supporting Information). Based on gene expression levels in GEO dataset (GSE44076), FUS levels were significantly upregulated in CRC and adjacent colon tissues as compared to non‐adjacent normal colon tissues (Figure 7E). Moreover, we performed Kaplan‐Meier survival curve analysis and observed that CRC patients with high expression of FUS had a significantly shorter overall survival time compared to those with low expression of FUS (Figure 7F). Notably, we also found that there was a significant positive correlation between FUS and circHIPK2 (Figure 7G). Taken together, these results indicate that FUS serves as a potential mediator that promotes the biogenesis of circHIPK2.

## 3 Discussion

Profiling the expression of circRNAs is crucial for the identification and characterization of novel circRNAs, as well as for unraveling their underlying mechanisms and functions.^[^ ^10a,b^ ^]^ In this study, we uncovered that circHIPK2, originating from the HIPK2 gene, played a pivotal role in both IBD and CRC, marking it as a potential key factor in the shared pathophysiology of these conditions. Several lines of evidence support this notion. First, our comprehensive analysis of GEO datasets identified circHIPK2 as the only circRNA consistently upregulated in both IBD and CRC. We further validated the upregulation of circHIPK2 in clinical samples from patients with IBD, CRC, and DSS‐induced colitis. These observations align with a growing body of evidence demonstrating the influence of circHIPK2 in cancer and inflammatory conditions.^[^ ^20^ ^]^ In addition, the differential expression of circHIPK2 in colitis and CRC also suggested its potential role as a biomarker in these diseases. In this context, circHIPK2 emerges as a central player.

As one of the serine/threonine kinases, HIPK2 is wildly involved in the progression of multiple diseases including cancer, fibrosis, angiogenesis, and neurological diseases.^[^ ^21^ ^]^ CircHIPK2 was derived from the Exon2 of HIPK2 gene. Recent studies found that circHIPK2 could regulate astrocyte activation via cooperation of autophagy and ER stress.^[^ ^20f^ ^]^ Furthermore, circHIPK2 promoted lipopolysaccharide‐induced astrocytic inflammation.^[^ ^20a^ ^]^ In addition, circHIPK2 was associated with DDP‐resistant and could promote ovarian cancer.^[^ ^20e^ ^]^ In the present study, we indicated that circHIPK2 exacerbated colitis, leading to increased inflammation, extensive tissue damage, and a shift in cytokine profiles. Conversely, circHIPK2 demonstrated an oncogene role in colitis‐associated tumorigenesis and CRC, where its downregulation led to fewer colonic tumors and a reduction in cell proliferation. These findings suggested a protective role for circHIPK2 in the context of IBD, while also implicating its promotion in the progression of CRC, highlighting new avenues for therapeutic interventions in these diseases by targeting circHIPK2. However, the bidirectional effects of circHIPK2 on colitis and tumorigenesis are particularly noteworthy and may provide a basis for designing tailored therapeutic strategies based on the disease stage.

The increased risk of CRC in IBD patients has been attributed to long‐standing multiple rounds of intestinal epithelial damage and repair, often in response to excessive inflammation.^[^ ^22^ ^]^ Also, the occurrence of genetic and epigenetic aberrations in signaling networks is a frequent phenomenon in IBD and CRC.^[^ ^1^, ^5^ ^]^ Accumulating data indicated the shared common features between IBD and CRC such as abnormal DNA methylation lineage, RAC1‐dependent cytoskeleton dynamics, and Hippo signaling pathway,^[^ ^6^, ^23^ ^]^ although the mechanisms underlying them remain elusive. Intriguingly, our findings linked circHIPK2 to the Hippo signaling pathway. CircHIPK2 enhanced the translation of TAZ, a core component of the Hippo pathway, ultimately leading to the upregulation of YAP/TAZ target genes, including CCN1 and CCN2. We found that treatment with CCN1/2 recombinant protein or overexpression of CCN1/2 significantly indeed promoted cell proliferation. Several studies have also shown similar effects of CCN1/2 on cell proliferation. For example, CCN1 induced cholangiocyte proliferation and identified the CCN1/αvβ5/NF‐κB/JAG1 axis as critical for biliary injury repair.^[^ ^24^ ^]^ CCN1 stimulated the proliferation and differentiation of osteoblasts in vitro and contributed to bone remodeling in vivo in myeloma bone disease.^[^ ^25^ ^]^ In colon‐related cells, CCN1 promoted CRC progression by activating epithelial‐mesenchymal transition.^[^ ^26^ ^]^ CCN1 protein fully rescued Ccn1^dm/dm^ mice from DSS‐induced mortality and restored intestinal epithelial cell (IEC) proliferation, indicating that CCN1 is critical for intestinal mucosal healing.^[^ ^7c^ ^]^ CCN1 coordinately regulated ISC proliferation and differentiation during homeostasis. Deletion of Ccn1 in ISCs resulted in enhanced ISC proliferation and increased secretory cells at the expense of absorptive cells, suggesting that upregulated Ccn1 may inhibit ISC and secretory cell proliferation while increasing absorptive cells.^[^ ^9^ ^]^ Overall, CCN1 exerts diverse cellular activities in a cell type‐specific manner, largely through its direct binding to specific integrin receptors.^[^ ^27^ ^]^ These above studies further support the significant role of the Hippo pathway in the intestinal epithelial of IBD and CRC. Thus, targeting circHIPK2 might offer a promising approach to regulate Hippo signaling and mitigate the effects of dysregulated YAP/TAZ activation. More interestingly, we discovered that an RNA binding protein, FUS can combine with the upstream and downstream regions of the circHIPK2 pre‐mRNA and is a key mediator promoting the biogenesis of circHIPK2. The significant correlation between FUS and circHIPK2 levels indicated that targeting FUS might be a potential approach to manipulate circHIPK2 expression. Nevertheless, further research into the molecular details of this interaction is required to develop more precise therapeutic strategies.

The Hippo transducers YAP/TAZ have been shown to play both positive and negative roles in Wnt signaling. Several mechanisms have been proposed for how YAP regulates Wnt signaling. YAP and TAZ are integral components of the β‐catenin destruction complex, serving as a cytoplasmic sink for YAP/TAZ. In Wnt‐OFF cells, YAP/TAZ are essential for β‐TrCP recruitment to the complex and β‐catenin inactivation. However, β‐catenin inhibition by YAP/TAZ still requires the well‐documented β‐catenin phosphorylation by GSK3. In Wnt‐ON cells, YAP/TAZ are released from the complex and accumulate in the nucleus, which is required for crypt overgrowth induced by APC loss and regeneration.^[^ ^28^ ^]^ Furthermore, YAP/TAZ act as bona fide downstream effectors of the alternative Wnt signaling pathway.^[^ ^29^ ^]^ YAP regulates Wnt signaling through the expression of Wnt antagonist Dkk1, a YAP target gene.^[^ ^29^ ^]^ Additionally, transcriptionally active YAP in normal crypts and organoids, regulated by CCN1‐integrins α_v_β_3_/α_v_β_5_ signaling, controls Wnt signaling and restricts ISC proliferation.^[^ ^9^ ^]^ Upon tissue injury, YAP reprograms Lgr5(+) ISCs by suppressing Wnt signaling and excessive Paneth cell differentiation, while promoting cell survival and inducing a regenerative program that includes EGF pathway activation.^[^ ^30^ ^]^ Moreover, CCN1 and YAP positively regulate each other in a regulatory loop in the regulation of ISCs.^[^ ^9^ ^]^ Together, the relationships among YAP/TAZ, CCN, and Wnt signaling are complex, exerting their biological functions in context‐dependent manners.

EIF4A3 is a core component of the EJC, which is involved in post‐ transcriptional regulation processes including nonsense‐mediated mRNA decay, mRNA splicing, transport, and translation.^[^ ^31^ ^]^ Also, EIF4A3 could be recruited by long non‐coding RNAs to regulate the expression of certain proteins in tumors.^[^ ^31^, ^32^ ^]^ Notably, circHIPK2 displayed a predominantly cytoplasmic localization in CRC cells, suggesting its potential involvement in post‐transcriptional processes. More importantly, our study also revealed the mechanistic basis for circHIPK2's impact on the Hippo pathway. We identified the interaction between circHIPK2 and EIF4A3, which forms a complex to specifically enhance the translational efficiency of TAZ mRNA. Although previous studies showed that EIF4A3 promoted the biogenesis of circRNAs,^[^ ^18^, ^33^ ^]^ this is the first to demonstrate that EIF4A3 could be recruited by circRNAs to exert biological functions in gastrointestinal disorders. Nevertheless, our study still leaves some questions unanswered, such as the specific EIF4A3 binding motifs on circHIPK2, other factors involved in EIF4A3‐circHIPK2 complex, the function of circHIPK2 in other cell types, such as immune cells apart from epithelial cells, and the precise mechanism by which circHIPK2 exerts its bidirectional effects on colitis and tumorigenesis.

In conclusion, as summarized in Figure S8 (Supporting Information), our study unveils circHIPK2 as a pivotal player in the shared mechanisms of CRC and IBD. By modulating the Hippo signaling pathway and downstream target genes, circHIPK2 offers a promising avenue for the development of innovative therapeutic strategies to combat these gastrointestinal disorders. The bidirectional effects of circHIPK2 in colitis and colitis‐associated tumorigenesis emphasize the importance of context‐dependent approaches to harness its therapeutic potential. Therefore, further investigations and clinical studies will be essential to fully realize the translational potential of circHIPK2‐based therapies.

## 4 Experimental Section

### Cell Culture and Clinical Specimens

The cell lines HCT116, RKO, DLD‐1, SW620, SW480, LoVo, HT‐29, LS174T, MC38 and HEK293T were obtained from ATCC (Manassas, VA, USA). The NCM460 cell line was procured from BNCC (Beijing, China). HCT116, SW620, SW480, and HT‐29 cells were maintained in McCoy's 5A medium (Biological Industries (BI), Israel). RKO, DLD‐1, LS174T, NCM460, and HEK293T cells were cultured in DMEM (BI). LoVo and MC38 cells were cultured in RPMI 1640 medium (BI). The media were supplemented with 10% fetal bovine serum (Gibco) and 1% penicillin‐streptomycin mixture, and cells were incubated at 37 °C in a 5% CO_2_ incubator. All cell lines were authenticated by genetic profiling using polymorphic short tandem repeat loci. Twenty‐three paired fresh primary colorectal tumors and matched adjacent normal tissues as previously described,^[^ ^34^ ^]^ and tissues from nine healthy controls and patients with inflammatory bowel disease (10 with CD and 18 with UC) (Table S8, Supporting Information), were obtained with informed patient consent and approval from the Institutional Ethics Committee of the Second Affiliated Hospital of Wenzhou Medical University (2023‐K‐44‐01).

### Microarray Data Analysis

Microarray datasets (GEO accession numbers: GSE126094, GSE138589, and GSE131911) were retrieved from the Gene Expression Omnibus. Dysregulated circRNAs in colorectal samples were identified based on significant fold changes (upregulated by ≥1.5‐fold or downregulated by ≤0.67‐fold, with *P* < 0.05).

### siRNA, Plasmid, and Transfection

siRNAs were acquired from GenePharma (Shanghai, China) and transfected at final concentrations of 20 nm or 50 nm using Lipofectamine 6000 Reagent (Beyotime, Shanghai, China). CCN1 and CCN2 overexpression plasmids (Genecopoeia, Rockville, MD, USA) were transiently transfected using Lipofectamine 6000 Reagent. Cells were collected 48 h post‐transfection for subsequent analysis. The siRNA sequences are listed in Table S7 (Supporting Information).

### Establishment of Stable CRC Cell Lines

To generate stable circHIPK2‐knockdown cell lines, lentiviral particles were produced in HEK293T cells co‐transfected with shcircHIPK2, shcircHipk2, or corresponding empty vectors, along with psPAX2 and pMD2.G plasmids. HCT116, RKO, and MC38 cells were infected with lentiviral particles in the presence of 8 µg mL^−1^ polybrene (Genechem, Shanghai, China) and selected using 0.5 µg mL^−1^ puromycin. After 1 week, puromycin‐resistant cell pools were collected and verified by reverse‐transcription quantitative PCR (RT‐qPCR).

### RT‐qPCR

Total RNA was extracted using TRIzol (Beyotime). To quantify mRNA levels, cDNA was synthesized using Hiscript II Q RT SuperMix for qPCR (+gDNA wiper) (Vazyme Biotech, Nanjing, China), and PCR was conducted using ChamQ Universal SYBR qPCR Master Mix (Vazyme). β‐actin mRNA levels served as a control. The qPCR primer sequences are listed in Table S7 (Supporting Information).

### Cytoplasmic and Nuclear RNA Isolation

The Nuclear and Cytoplasmic Protein Extraction Kit (Beyotime) was employed following the manufacturer's instructions. CircHIPK2 expression was detected by RT‐qPCR.

### Fluorescence In Situ Hybridization (FISH)

FISH was performed to visualize the subcellular location of circHIPK2 using the FISH kit (GenePharma) according to the manufacturer's protocol. Briefly, Cells were fixed with 4% paraformaldehyde and penetrated with 0.1% Triton X‐100. After incubation with blocking solution, cells were incubated with digoxigenin‐labeled circHIPK2‐specific probes at 37 °C overnight. After washing with Tween 20 and SSC buffer, cells were stained with DAPI to counterstain the nuclei. Cells were imaged and analyzed using a confocal microscope.

### Cell Proliferation, Colony Formation Assay, and Cell‐Cycle Analysis

Cell proliferation and colony formation assay were performed as described previously.^[^ ^34^ ^]^ For CCN1/2 treatment, CCN1/2 recombinant protein (MedChemExpress, Shanghai, China) at final concentration of 100 ng mL^−1^ was added into cell culture medium for subsequent analysis. For cell‐cycle analysis, treated cells were harvested and fixed with pre‐cooled 70% ethanol at 4 °C overnight. Cells were analyzed for DNA content by flow cytometry as described previously.^[^ ^34^ ^]^

### Immunohistochemistry and Hematoxylin and Eosin (H&E) Staining Assay

Immunohistochemistry analysis was performed as described previously,^[^ ^34^ ^]^ and the anti‐Ki67 (sc‐23900, Santa Cruz) antibody was used. Hematoxylin and eosin (H&E) staining Kit (Solarbio, Beijing, China) was employed following the manufacturer's instructions.

### Immunofluorescence

Immunofluorescence assays were performed as described previously.^[^ ^35^ ^]^ The primary antibody, anti‐WWTR1/TAZ (23306‐1‐AP), was obtained from Proteintech. Anti‐YAP (#14 074) was obtained from Cell Signaling Technology. The secondary antibody Cy3‐conjugated donkey anti‐rabbit (D110052‐010) was obtained from Sangon Biotech.

### Western Blotting

Western blot analysis was performed as described previously.^[^ ^34^ ^]^ The primary antibodies were as follows: anti‐WWTR1/TAZ (23306‐1‐AP, Proteintech), anti‐phosphorylated TAZ (Ser89) (#59 971, Cell Signaling Technology), anti‐YAP (#14 074, Cell Signaling Technology), anti‐phosphorylated YAP (Ser127) (#13 008, Cell Signaling Technology), anti‐EIF4A3 (17504‐1‐AP, Proteintech), anti‐CTGF/CCN2 (23936‐1‐AP, Proteintech), anti‐CYR61/CCN1 (26689‐1‐AP, Proteintech), anti‐FUS/TLS (11570‐1‐AP, Proteintech), and anti‐GAPDH (#2118, Cell Signaling Technology).

### Animal Assays

All animal studies were reviewed and approved by the Institutional Ethics Committee of Wenzhou Medical University (wydw2021‐0131). For DSS‐AAV induced mice Model: Seven‐week‐old male C57BL/6J mice received intrarectal administration of AAVs expressing shcircHipk2 or shcon. One week later, acute colitis was induced by oral administration of 3% DSS (Yeasen Biotechnology, Shanghai, China) in drinking water for 7 days and followed by 2 days of normal drinking water. All mice were euthanized on day 18 and the intestines were dissected for subsequent analysis. For AOM/DSS‐AAV induced mice model: Seven‐week‐old male C57BL/6J mice were intraperitoneally injected with 12.5 mg kg^−1^ AOM (Sigma, Saint Louis, MO, USA), followed by a week of regular diet and water. Mice were then administered with AAVs containing shcircHipk2 twice and three cycles of 3% DSS for a week and drinking water for two weeks. Mice were fed with a regular diet for another five weeks, after which they were euthanized and the intestines were dissected for subsequent analysis. For xenograft studies: a total of 5×10^6^ shcircHIPK2‐RKO or control cells in 100 µL phosphate‐buffered saline (PBS) were subcutaneously injected into the rear flanks of the mice. Tumors were measured using a vernier caliper, and tumor volume was calculated using the formula: V = L×W^2^×0.5236 (where L = long axis and W = short axis). After a period of 20 days post‐inoculation, the mice were euthanized, and the tumors were collected and weighed.

### RNA Immunoprecipitation (RIP) Assay

RIP assay was performed as described previously.^[^ ^34^ ^]^ The primary antibodies anti‐EIF4A3 (17504‐1‐AP, Proteintech), anti‐FUS/TLS (11570‐1‐AP, Proteintech), or IgG control were used.

### RNA Pull‐Down and Mass Spectrometry Analysis

To validate the direct binding protein of circHIPK2, circRNA pull‐down assay was conducted using the MS2‐binding protein (MS2bp). First, the plasmids containing the circHIPK2 transcript fused with or without MS2bs elements (referred to as pMS2bs‐circHIPK2 and pcircHIPK2) were separately co‐transfected into HCT116 cells along with a construct encoding MS2bp‐Flag, respectively. Subsequently, immunoprecipitation was conducted using an anti‐Flag antibody, with IgG utilized as a negative control. The RNA‐bound protein complex was eluted and analyzed by following Mass spectrometry analysis.

### Polysome Fractionation Assay

The polysome fractionation assay was conducted to assess mRNA translation following previously established protocols.^[^ ^36^ ^]^ Briefly, sucrose solutions at 5%, 10%, 20%, 30%, 40%, and 50% were prepared and layered in ultracentrifuge tubes based on density. Polysomes were prepared in 425 µL of hypotonic buffer (5 mm Tris‐HCl pH 7.5, 2.5 mm MgCl_2_, 1.5 mm KCl, 1× protease inhibitor cocktail (EDTA‐free), 0.5% Triton X‐100, 10 mg mL^−1^ cycloheximide (CHX, Selleck, Houston, TX, USA), 1 M DTT, 100 units of RNase inhibitor, and 0.5% sodium deoxycholate). Following centrifugation at 16 000 ×g for 7 min at 4 °C, the supernatant containing polysomes was collected. Subsequently, the polysome supernatant was carefully loaded atop the sucrose gradient and subjected to ultracentrifugation at 36 000 rpm for 2 h at 4 °C. Fourteen polysome fractions were collected sequentially from the top to bottom of the resulting sucrose gradient. Total RNA was isolated from eight to fourteen fraction, and RT‐qPCR was performed to determine the expression of circHIPK2, TAZ, and YAP in each fraction.

### Dual‐Luciferase Reporter Assay

The promoter regions of CCN1 and CCN2 were amplified using specific PCR primers and subsequently cloned into pGL6‐TA vector utilizing the In‐Fusion HD Cloning Plus kit (TaKaRa, Dalian, China). For the reporter assays, HCT116 and RKO cells were co‐transfected with sicircHIPK2 along with the firefly luciferase reporter vector and the *Renilla* luciferase control vector (pRL‐CMV). 48 h post‐transfection, luciferase activities were measured using the Dual‐Glo luciferase assay system (Promega). The primer sequences for plasmid construction are listed in Table S7 (Supporting Information).

### CHX Assay, MG132 Assay, and RNA Decay Assay

For CHX assay and MG132 (Selleck) assay, cells were treated with 20 µM CHX or 10 µM MG132 for 6 h to inhibit the translation or degradation process, following by subsequent western blot analysis. For RNA decay assay, cells were treated with 2 µg mL^−1^ actinomycin D (ActD, Selleck) for the indicated time points, following by subsequent RT‐qPCR analysis. The decay rates were calculated by setting the RNA level at 0 h as 100% for both the treated and negative control groups. Exponential fitting curves were shown.

### Statistical Analysis

Data are presented as mean ± SD or ± SEM and were analyzed in GraphPad Prism 9 (GraphPad Software, La Jolla, CA, USA). Unless noted otherwise, each experiment was carried out in triplicate. Statistical significance was determined using two‐tailed Student's *t*‐test, Wilcoxon signed‐rank test, or Mann‐Whitney *U* test. Differences between groups were determined by two‐way analysis of variance (ANOVA). The correlation was determined using Pearson's correlation test. Differences with *P*‐value less than 0.05 were considered statistically significant.

## Conflict of Interest

The authors declare no conflict of interest.

## Author Contributions

X.Z., J.T., Q.Z., and C.W. contributed equally to this work. X.Z., J.T., and Q.Z. performed the main experiments and analyzed the data. J.Q., Y.W., J.X., K.Y., Z.Z., H.W., J.L., and Z.S. partially contributed to the experiments presented in this manuscript. C.W. and Y.J. collected the clinical samples and analyzed the data. J.W., J.Y.W., Q.Z., X.Z., and J.T. conceived the study, designed the experiments, and wrote the manuscript. All authors read and approved the final manuscript.

## Supporting information

Supporting Information

## Data Availability

The data that support the findings of this study are available from the corresponding author upon reasonable request.

## References

[1] a) H. Sung , J. Ferlay , R. L. Siegel , M. Laversanne , I. Soerjomataram , A. Jemal , F. Bray , Ca‐Cancer J. Clin. 2021, 71, 209; 33538338 10.3322/caac.21660

[2] a) G. G. Kaplan , Nature reviews. Gastroenterol. & hepatol. 2015, 12, 720; 10.1038/nrgastro.2015.150 26323879

[3] F. Bray , M. Laversanne , H. Sung , J. Ferlay , R. L. Siegel , I. Soerjomataram , A. Jemal , Ca‐Cancer J. Clin. 2024, 74, 229. 38572751 10.3322/caac.21834

[4] a) A. G. Long , E. T. Lundsmith , K. E. Hamilton , Current colorectal cancer reports 2017, 13, 341; 29129972 10.1007/s11888-017-0373-6 PMC5678998

[5] S. C. Shah , S. H. Itzkowitz , Gastroenterol. 2022, 162, 715. 10.1053/j.gastro.2021.10.035 PMC9003896 34757143

[6] a) Z. Xie , Y. Wang , G. Yang , J. Han , L. Zhu , L. Li , S. Zhang , Cell Death Dis. 2021, 12, 79; 33436549 10.1038/s41419-021-03395-3 PMC7804279

[7] a) H. Yeger , B. Perbal , J. cell commun. and signal. 2021, 15, 491; 33877533 10.1007/s12079-021-00618-2 PMC8642525

[8] H. W. Koon , D. Zhao , H. Xu , C. Bowe , A. Moss , M. P. Moyer , C. Pothoulakis , Am. J. Pathol. 2008, 173, 400. 18599605 10.2353/ajpath.2008.080222 PMC2475777

[9] J. H. Won , J. S. Choi , J. I. Jun , Nat. Commun. 2022, 13, 3117. 35660741 10.1038/s41467-022-30851-1 PMC9166801

[10] a) C. X. Liu , L. L. Chen , Cell 2022, 185, 2016; 35584701 10.1016/j.cell.2022.04.021

[11] L. Xiao , X. X. Ma , J. Luo , H. K. Chung , M. S. Kwon , T. X. Yu , J. N. Rao , R. Kozar , M. Gorospe , J. Y. Wang , Gastroenterol. 2021, 161, 1303. 10.1053/j.gastro.2021.05.060 PMC8463477 34116030

[12] D. Wan , S. Wang , Z. Xu , X. Zan , F. Liu , Y. Han , M. Jiang , A. Wu , Q. Zhi , Clin. Transl. Med. 2022, 12, e683. 35184406 10.1002/ctm2.683 PMC8858608

[13] a) L. Zheng , H. Liang , Q. Zhang , Z. Shen , Y. Sun , X. Zhao , J. Gong , Z. Hou , K. Jiang , Q. Wang , Y. Jin , Y. Yin , Molecular cancer 2022, 21, 41; 35135542 10.1186/s12943-022-01495-y PMC8822707

[14] a) Z. Chen , R. Ren , D. Wan , Y. Wang , X. Xue , M. Jiang , J. Shen , Y. Han , F. Liu , J. Shi , Y. Kuang , W. Li , Q. Zhi , Oncogene 2019, 38, 6017; 31300733 10.1038/s41388-019-0857-8

[15] P. Glažar , P. Papavasileiou , N. Rajewsky , RNA 2014, 20, 1666. 25234927 10.1261/rna.043687.113 PMC4201819

[16] L. L. Chen , Nat. Rev. Mol. Cell Biol. 2020, 21, 475. 32366901 10.1038/s41580-020-0243-y

[17] a) N. H. Gehring , S. Lamprinaki , A. E. Kulozik , M. W. Hentze , Cell 2009, 137, 536; 19410547 10.1016/j.cell.2009.02.042

[18] a) X. Jiang , S. Guo , S. Wang , Y. Zhang , H. Chen , Y. Wang , R. Liu , Y. Niu , Y. Xu , Cancer Res. 2022, 82, 831; 34965937 10.1158/0008-5472.CAN-21-2988

[19] X. Cao , B. Zhao , Methods in molecular biol. 2019, 1893, 137. 10.1007/978-1-4939-8910-2_12 30565132

[20] a) R. Huang , L. Cai , X. Ma , K. Shen , Int. Immunopharmacol. 2023, 117, 109907; 36827915 10.1016/j.intimp.2023.109907

[21] a) A. Garufi , V. D'Orazi , G. Pistritto , M. Cirone , G. D'Orazi , Cancers 2023, 15, 2678; 37345014 10.3390/cancers15102678 PMC10216817

[22] a) M. Lucafò , D. Curci , M. Franzin , G. Decorti , G. Stocco , Frontiers in pharmacol. 2021, 12, 772101; 10.3389/fphar.2021.772101 PMC8563785 34744751

[23] a) L. D. C. Martínez‐Sánchez , P. A. Ngo , R. Pradhan , L. S. Becker , D. Boehringer , D. Soteriou , M. Kubankova , C. Schweitzer , T. Koch , V. Thonn , L. Erkert , I. Stolzer , C. Günther , C. Becker , B. Weigmann , M. Klewer , C. Daniel , K. Amann , S. Tenzer , R. Atreya , M. Bergo , C. Brakebusch , A. J. M. Watson , J. Guck , B. Fabry , I. Atreya , M. F. Neurath , R. López‐Posadas , Gut 2023, 72, 275; 35241625 10.1136/gutjnl-2021-325520 PMC9872254

[24] K. H. Kim , C. C. Chen , G. Alpini , L. F. Lau , J. Clin. Invest. 2015, 125, 1886. 25822023 10.1172/JCI79327 PMC4463205

[25] H. Liu , F. Peng , Z. Liu , F. Jiang , L. Li , S. Gao , G. Wang , J. Song , E. Ruan , Z. Shao , R. Fu , Int. J. oncol. 2017, 50, 631. 28035364 10.3892/ijo.2016.3815

[26] S. Rasool , Q. A. Ismaeel , S. H. Arif , Am. J. Cancer Res. 2023, 13, 4872. 37970355 PMC10636662

[27] a) L. F. Lau , J. cell commun. and signal. 2016, 10, 121; 27098435 10.1007/s12079-016-0324-z PMC4882306

[28] L. Azzolin , T. Panciera , S. Soligo , E. Enzo , S. Bicciato , S. Dupont , S. Bresolin , C. Frasson , G. Basso , V. Guzzardo , A. Fassina , M. Cordenonsi , S. Piccolo , Cell 2014, 158, 157. 24976009 10.1016/j.cell.2014.06.013

[29] H. W. Park , Y. C. Kim , B. Yu , T. Moroishi , J. S. Mo , S. W. Plouffe , Z. Meng , K. C. Lin , F. X. Yu , C. M. Alexander , C. Y. Wang , K. L. Guan , Cell 2015, 162, 780. 26276632 10.1016/j.cell.2015.07.013 PMC4538707

[30] A. Gregorieff , Y. Liu , M. R. Inanlou , Y. Khomchuk , J. L. Wrana , Nature 2015, 526, 715. 26503053 10.1038/nature15382

[31] a) J. Ye , X. She , Z. Liu , Z. He , X. Gao , L. Lu , R. Liang , Y. Lin , Frontiers in oncol. 2021, 11, 712045; 10.3389/fonc.2021.712045 PMC8386015 34458150

[32] X. Wang , M. Chen , L. Fang , Mol Ther Nucleic Acids 2021, 26, 122. 34513299 10.1016/j.omtn.2021.07.003 PMC8413675

[33] Y. Liu , J. Song , H. Zhang , Z. Liao , F. Liu , C. Su , W. Wang , M. Han , L. Zhang , H. Zhu , Z. Zhang , H. Liang , L. Zhang , B. Zhang , X. Chen , J. exp. & clinical cancer res.: CR 2022, 41, 164. 35509064 10.1186/s13046-022-02378-2 PMC9069765

[34] K. Luo , J. Geng , Q. Zhang , Y. Xu , X. Zhou , Z. Huang , K. Q. Shi , C. Pan , J. Wu , J. exp. & clinical cancer res. 2019, 38, 249. 31186036 10.1186/s13046-019-1263-3 PMC6560732

[35] Z. Huang , Q. Li , K. Luo , Q. Zhang , J. Geng , X. Zhou , Y. Xu , M. Qian , J. A. Zhang , L. Ji , J. Wu , Cell Death Dis. 2019, 10, 372. 31068580 10.1038/s41419-019-1604-3 PMC6506554

[36] a) N. Wu , Z. Yuan , K. Y. Du , L. Fang , J. Lyu , C. Zhang , A. He , E. Eshaghi , K. Zeng , J. Ma , W. W. Du , B. B. Yang , Cell Death Differ. 2019, 26, 2758; 31092884 10.1038/s41418-019-0337-2 PMC7224378

