# Supplementary material for: CircHIPK2 Contributes Cell Growth in Intestinal Epithelial of Colitis and Colorectal Cancer through Promoting TAZ Translation
Source: Adv Sci (Weinh). 2024 Jul 9;11(34):2401588. doi: 10.1002/advs.202401588 (PMC11425914; doi:10.1002/advs.202401588)
Supplement: Supplementary file 1 — Supporting Information [file ADVS-11-2401588-s001.pdf]

ADVANCED SCIENCE
Open Access

Supporting Information

for *Adv. Sci.*, DOI 10.1002/advs.202401588

CircHIPK2 Contributes Cell Growth in Intestinal Epithelial of Colitis and Colorectal Cancer through Promoting TAZ Translation

Xixi Zeng, Jielin Tang, Qian Zhang, Chenxing Wang, Ji Qi, Yusi Wei, Jiali Xu, Kaiyuan Yang, Zuolin Zhou, Hao Wu, Jiarong Luo, Yi Jiang, Zengqiang Song, Jinyu Wu and Jianmin Wu**

CircHIPK2 contributes cell growth in intestinal epithelial of colitis and colorectal cancer through promoting TAZ translation

Xixi Zeng[#], Jielin Tang[#], Qian Zhang[#], Chenxing Wang[#], Ji Qi, Yusi Wei, Jiali Xu, Kaiyuan Yang, Zuolin Zhou, Hao Wu, Jiarong Luo, Yi Jiang, Zengqiang Song, Jinyu Wu*, Jianmin Wu*

Figure S1

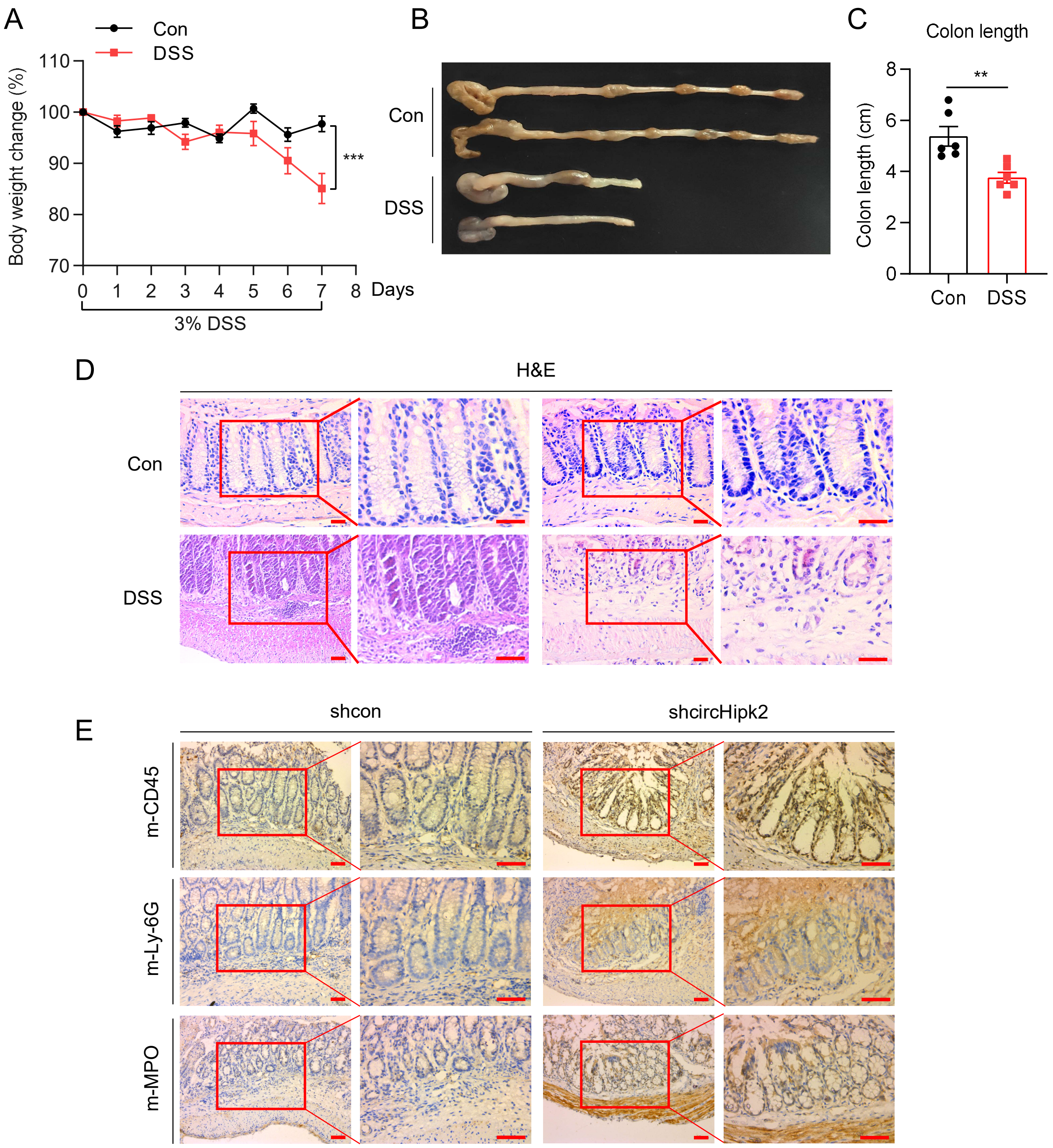

Figure S1 Setup of DSS-induced acute colitis mice model. Seven-week-old male C57BL/6 mice were administered 3% DSS in drinking water for a period of 7 days (n = 6). **A** Relative body weight curves. **B** Representative gross images of the colon of mice. **C** Measurement of colon length. **D** Representative HE-stained colon sections showing inflammatory infiltration (Scale bar: 50 μm)**. E** Representative images of CD45, Ly-6G and MPO-stained colon sections in shcircHipk2 and shcon group (Scale bar: 20 μm). (A) and (C) are presented as the mean ± SEM. **P < 0.01, *** P < 0.001 by two-way ANOVA in (A), or Mann-Whitney U test in (C).

Figure S2

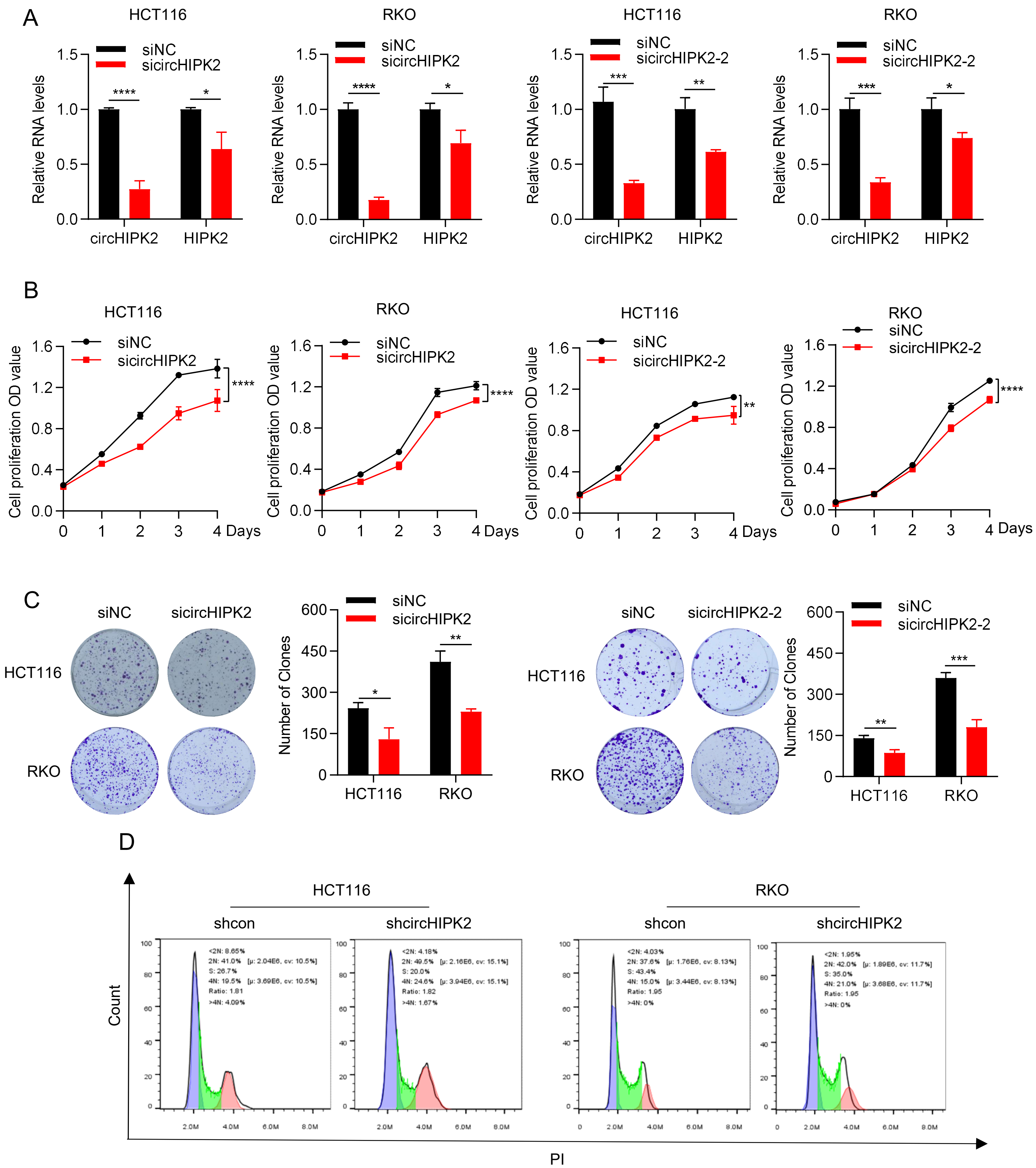

Figure S2 Knockdown of circHIPK2 inhibits cell proliferation of CRC. **A** Knockdown efficiency of circHIPK2 in CRC cells transfected with sicircHIPK2. **B-C** Decreased cell proliferation observed in circHIPK2 knockdown cells compared to control, as demonstrated by MTS assay (**B**) and colony formation assay (**C**). **D** Representative graphs of raw data for the cell cycle determined by flow cytometry (related to Fig. 3E). (A-C) are presented as the mean ± SD. *P < 0.05, **P < 0.01, *** P < 0.001, ****P < 0.0001 by Student's t-test in (A) and (C), or two-way ANOVA in (B).

Figure S3

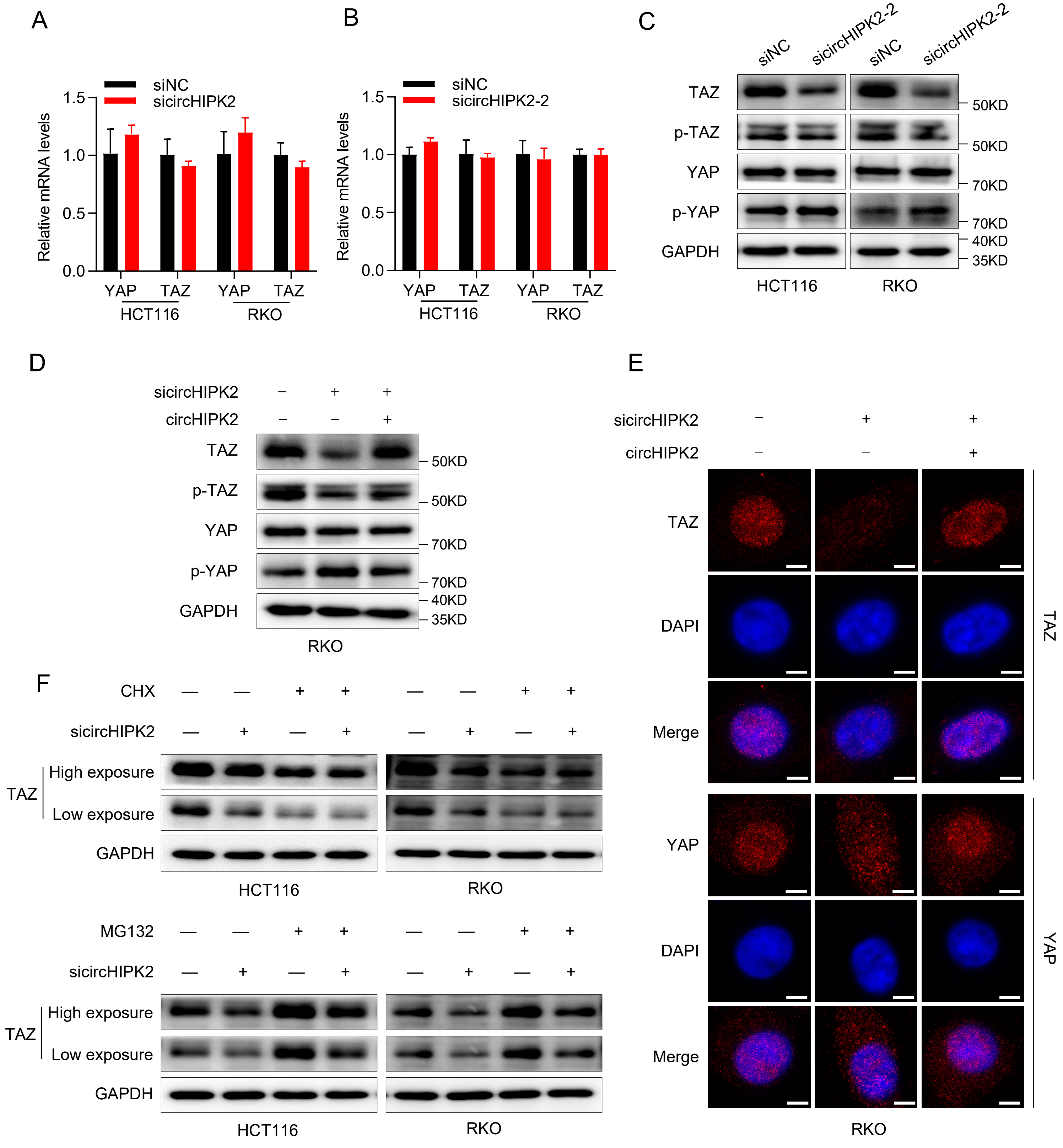

Figure S3 The effects of circHIPK2 on the expression of TAZ and YAP in CRC cells. **A-B** The mRNA levels of TAZ and YAP in circHIPK2 knockdown cells using two different siRNA (sicircHIPK2, **A**; sicircHIPK2-2, **B**) were determined by RT-qPCR. **C** The protein levels of TAZ, phosphorylated TAZ (Ser89), YAP and phosphorylated YAP (Ser127) in sicircHIPK2-2 transfected cells were determined by western blotting. **D** Rescue of circHIPK2 expression restored the levels of TAZ, phosphorylated TAZ (Ser89), and phosphorylated YAP (Ser127) in RKO cells. **E** Subcellular localization of TAZ/YAP is restored following the rescue of circHIPK2 expression in RKO cells. **F** The protein levels of TAZ in circHIPK2 knockdown cells under CHX (20 μM) or MG132 (10 μM) treatments. Scale bar: 10 μm. (A) and (B) are presented as the mean ± SD.

Figure S4

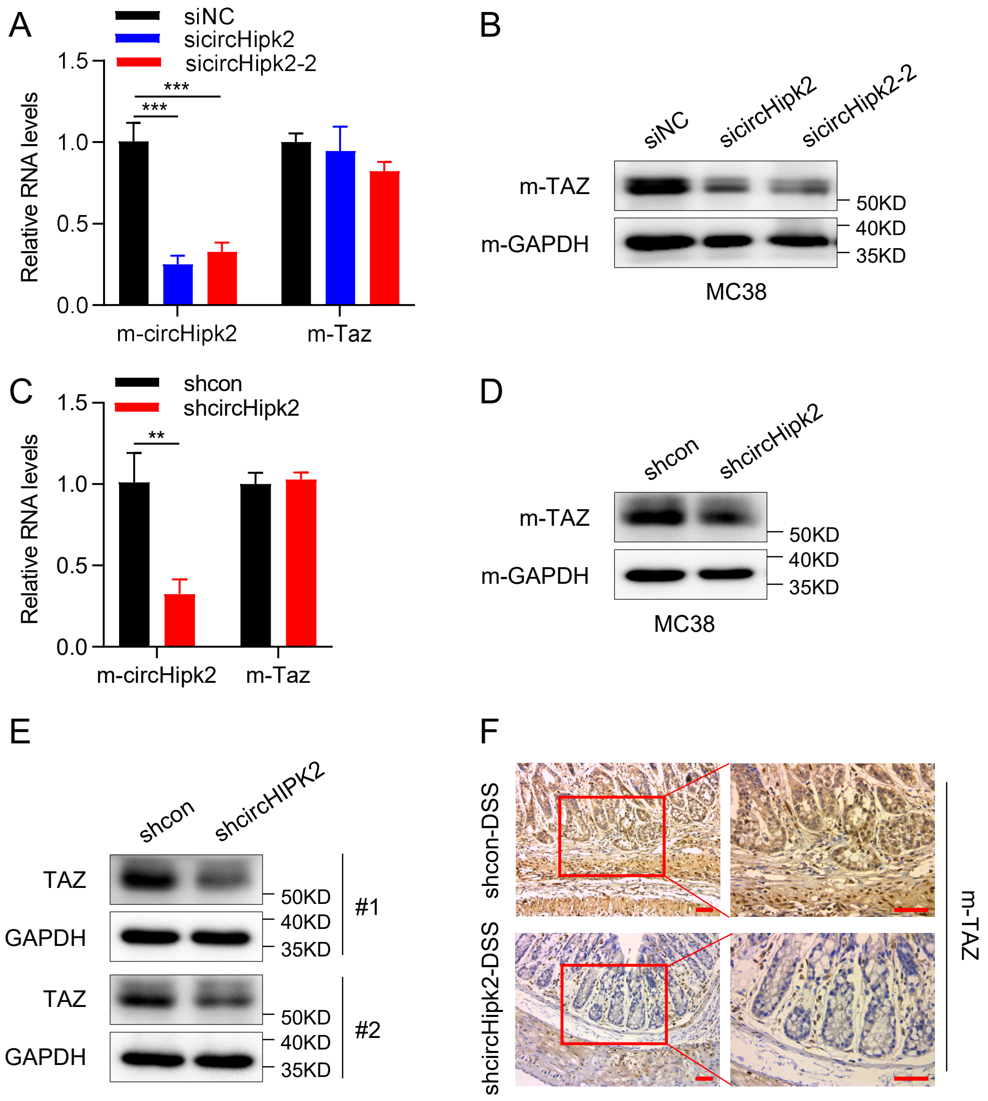

Figure S4 The effects of circHIPK2 on the expression of TAZ in mouse CRC cells and tumors. **A** The levels of m-circHipk2 and m-Taz in circHipk2 knockdown MC38 cells. **B** The protein levels of m-TAZ in circHipk2 knockdown MC38 cells were determined by western blotting. **C** The levels of m-circHipk2 and m-Taz in stable shcircHipk2-MC38 cells. **D** The protein levels of m-TAZ in stable shcircHipk2-MC38 cells. **E** The protein levels of TAZ in circHIPK2-knockdown xenografted tumors from nude mice. **F** IHC staining showed that the levels of m-TAZ were reduced in circHipk2-knockdown colon tissues from AOM/DSS induced mice model. Scale bar: 20 μm. (A) and (C) are presented as the mean ± SD. $^{**}P < 0.01$, $^{***}P < 0.001$ by Student's t-test in (A) and (C).

Figure S5

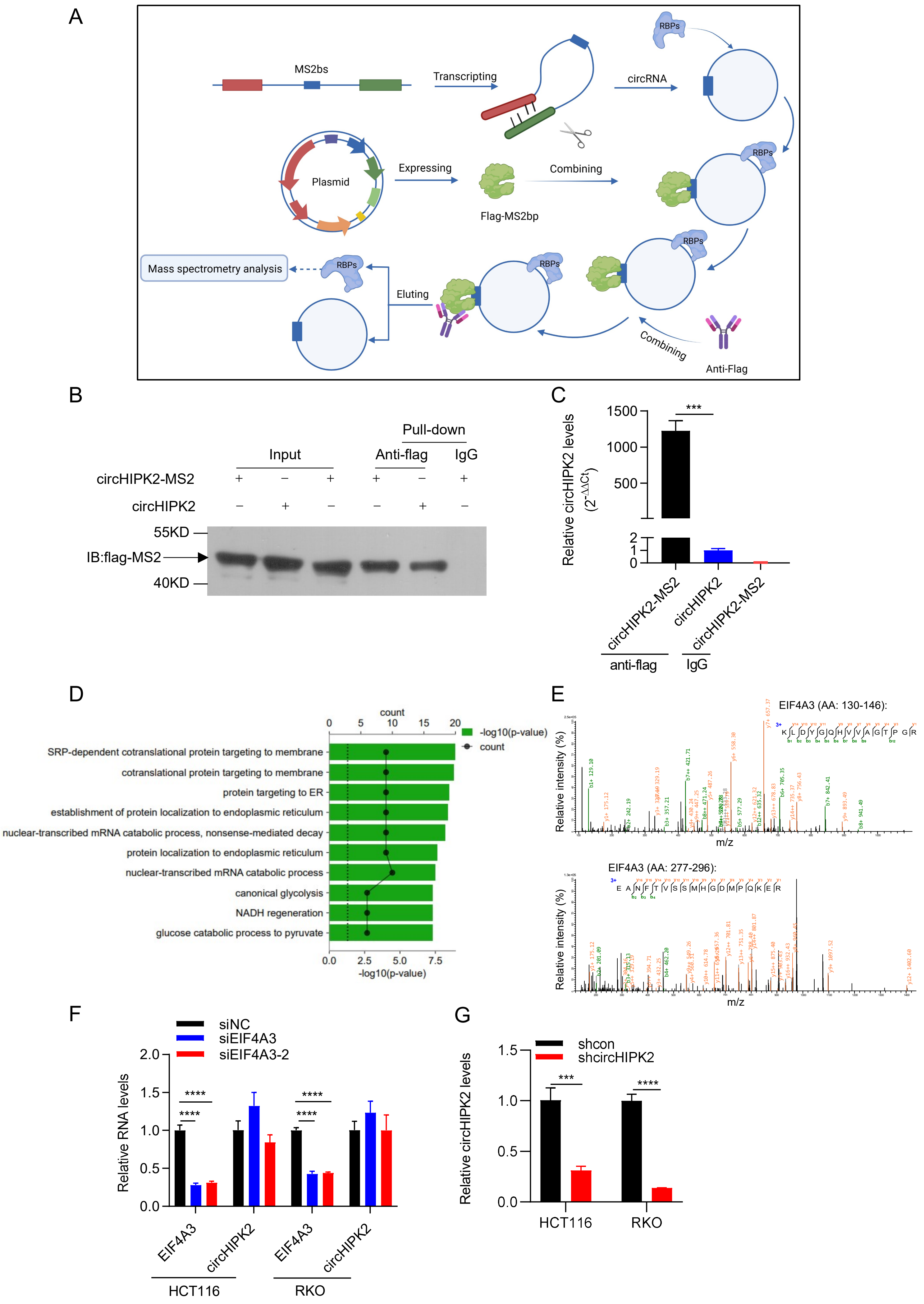
A
MS2bs
Transcripting
circRNA
RBPs
Plasmid
Expressing
Flag-MS2bp
Combining
Mass spectrometry analysis
Eluting
Combining
Anti-Flag
B
Pull-down
Input
Anti-flag
IgG
circHIPK2-MS2
circHIPK2
55KD
IB:flag-MS2
40KD
C
Relative circHIPK2 levels
(2-ΔΔCt)
***
circHIPK2-MS2
circHIPK2
circHIPK2-MS2
anti-flag
IgG
D
count
-log10(p-value)
SRP-dependent cotranslational protein targeting to membrane
cotranslational protein targeting to membrane
protein targeting to ER
establishment of protein localization to endoplasmic reticulum
nuclear-transcribed mRNA catabolic process, nonsense-mediated decay
protein localization to endoplasmic reticulum
nuclear-transcribed mRNA catabolic process
canonical glycolysis
NADH regeneration
glucose catabolic process to pyruvate
E
EIF4A3 (AA: 130-146):
EIF4A3 (AA: 277-296):
Relative intensity (%)
m/z
F
siNC
siEIF4A3
siEIF4A3-2
Relative RNA levels
****
EIF4A3
circHIPK2
HCT116
RKO
G
shcon
shcircHIPK2
Relative circHIPK2 levels
***
****
HCT116
RKO

Figure S5 Identification of circHIPK2-interacting proteins. **A** Schematic of experimental design for the circRNA pull-down assay to identify proteins binding to circHIPK2. MS2-binding protein (MS2bp) was employed to capture circHIPK2-associated proteins. **B** Validation of Flag-immunoprecipitation (IP) efficiency by western blotting. **C** Validation of specific enrichment of circHIPK2 in the pull-down samples containing MS2bs (pMS2bs-circHIPK2) by RT-qPCR. **D** Gene Ontology (GO) enrichment analysis of proteins bound to circHIPK2. **E** Representative images of detection of EIF4A3 in the precipitate obtained from RNA pull-down by LC-MS/MS. **F** The levels of circHIPK2 were measured following EIF4A3 knockdown. **G** Knockdown efficiency of circHIPK2 in stable shcircHIPK2-HCT116 and -RKO CRC cell lines. (C), (F) and (G) are presented as the mean ± SD. ***$P < 0.001$, ****$P < 0.0001$ by Student's t-test in (C), (F) and (G).

Figure S6

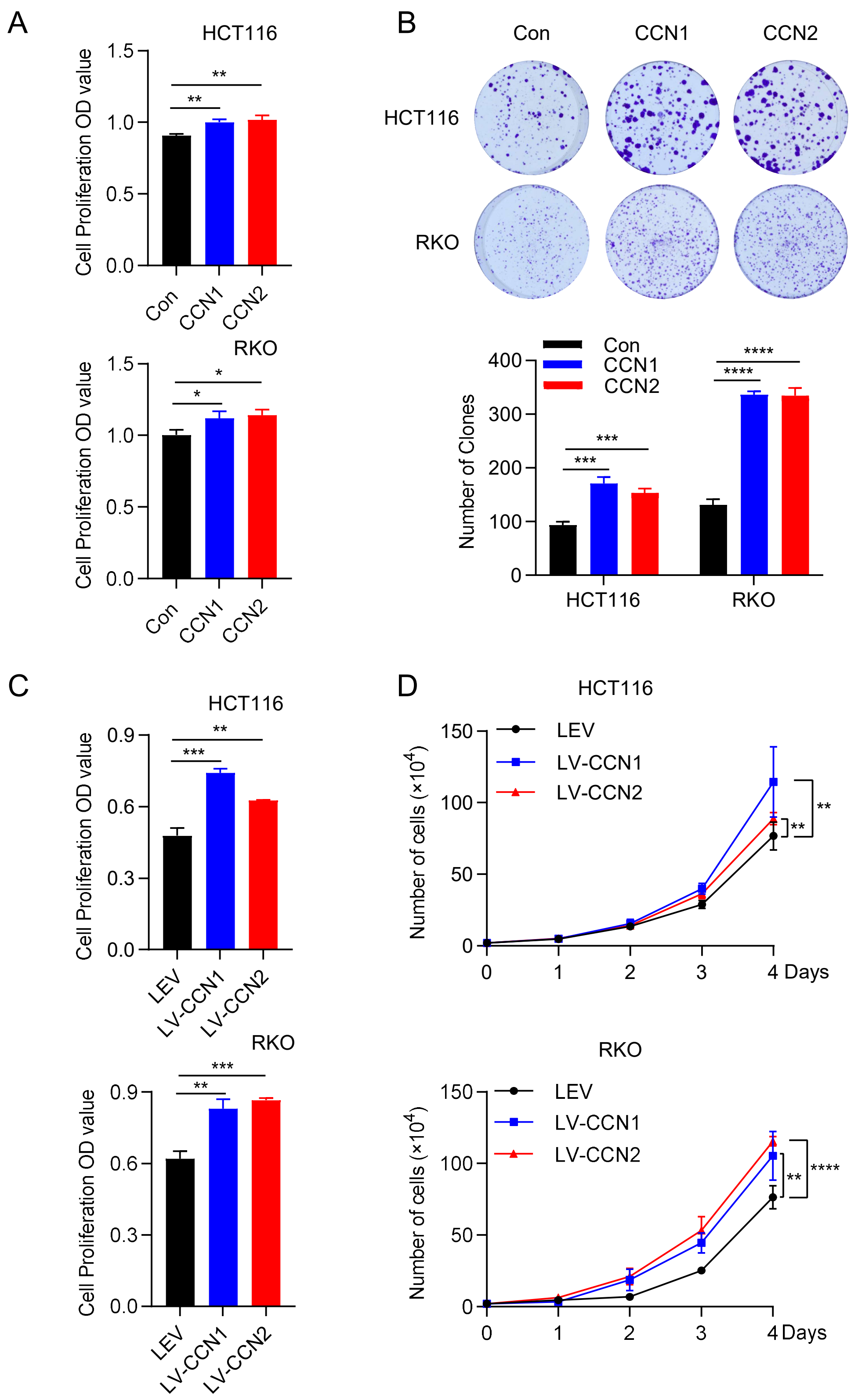

Figure S6 The effects of CCN1 and CCN2 on cell proliferation of CRC. **A-B** treatment with CCN1/2 recombinant protein significantly promoted cell proliferation, as assessed by MTS assay (**A**), and colony formation assays (**B**) in both HCT116 and RKO cell lines. **C-D** Increased cell proliferation observed in CCN1/2 overexpression CRC cells compared to control, as demonstrated by MTS assay (**C**) and cell count (**D**). (A-D) are presented as the mean ± SD. *P < 0.05, **P < 0.01, ***P < 0.001, ****P < 0.0001 by Student's t-test in (A-C) or two-way ANOVA in (D).

Figure S7

A

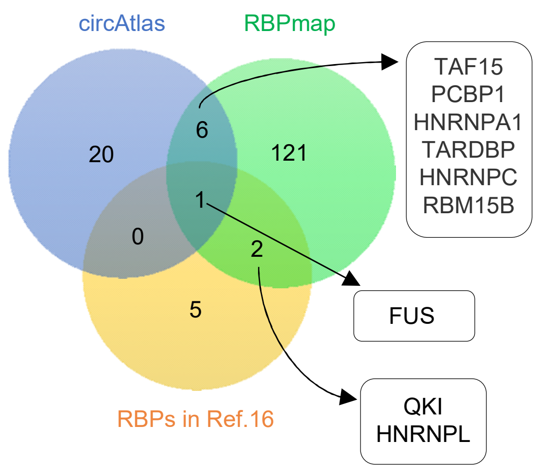

B

Tag Name	Sequence Position	Z-score	P-value	Upstream/Downstream
a	-1827	2.081	1.87E-02	Upstream
	-1809	2.387	8.49E-03	
	-1808	2.387	8.49E-03	
b	-986	1.96	2.50E-02	Upstream
	-977	1.96	2.50E-02	
	-973	2.153	1.57E-02	
c	1071	1.766	3.87E-02	Downstream
	1087	1.766	3.87E-02	
	1090	1.766	3.87E-02	
d	1300	2.446	7.22E-03	Downstream
	1325	1.892	2.92E-02	
e	1760	1.892	2.92E-02	Downstream

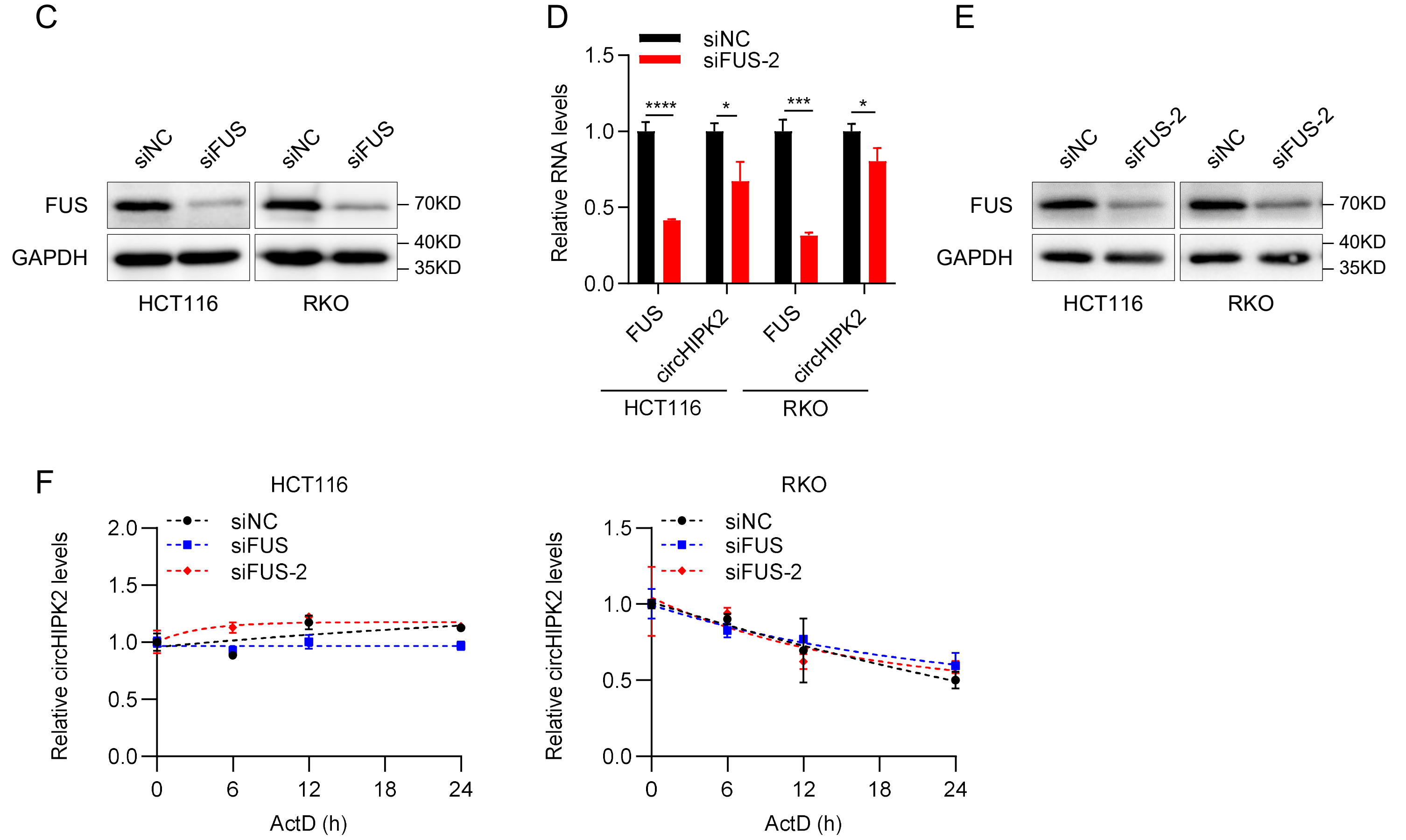

Figure S7 Screening of RBPs involved in circHIPK2 biogenesis. **A** Venn diagram depicting the number of predicted RBPs binding to flanking regions of circHIPK2 pre-mRNA. **B** Prediction of the putative FUS binding sites in the flanking sequences of circHIPK2 pre-mRNA by RBPmap database. **C** The protein levels of FUS were assessed by western blotting following FUS knockdown. **D** The relative levels of circHIPK2 were determined upon FUS knockdown using another siFUS by RT-qPCR. **E** The protein levels of FUS were assessed by western blotting in CRC cells transfected with siFUS-2. **F** The decay of circHIPK2 was monitored by RT-qPCR in FUS-knockdown CRC cells treated with actinomycin D (ActD, 2 μg/mL) for the indicated time points upon normalization to RNA input levels. (D) and (F) are presented as the mean ± SD. $^*P < 0.05$, $^{***}P < 0.001$, $^{****}P < 0.0001$ by Student's t-test in (D).

Figure S8

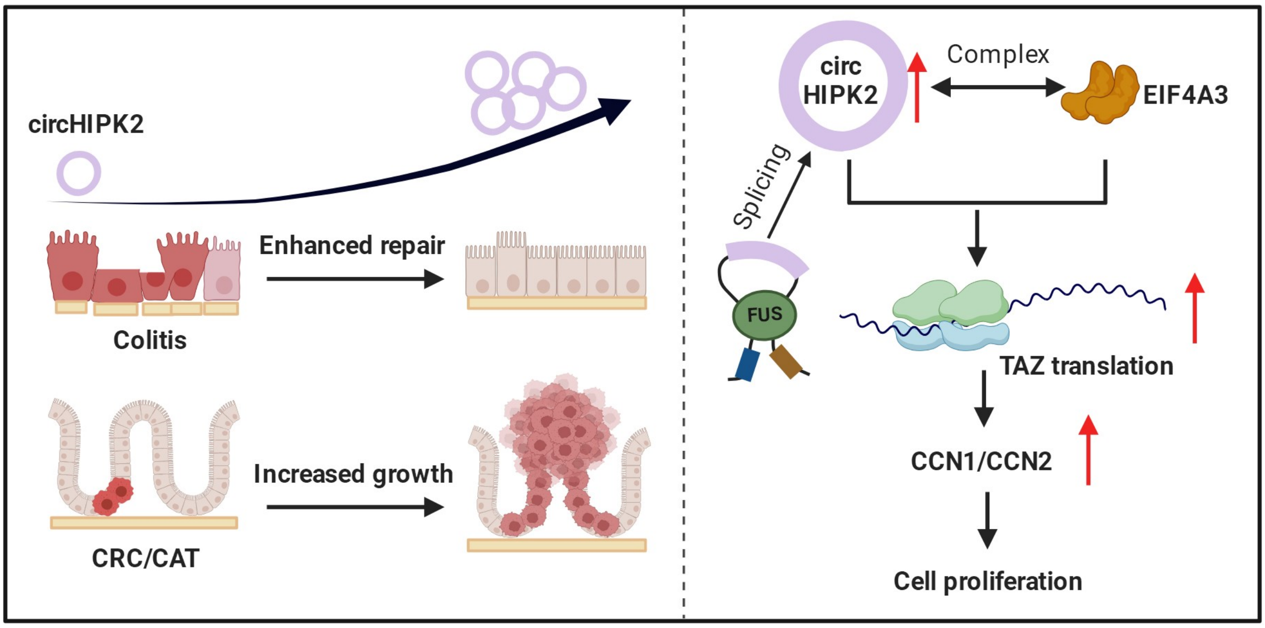

Figure S8 Schematic summary of the role of circHIPK2 in IBD and CRC. Circular RNA circHIPK2 emerges as a key player in the shared mechanisms of IBD and CRC. circHIPK2 mediated by FUS, interacted with EIF4A3 to promote the translation of TAZ, ultimately increase the transcription of downstream target genes CCN1 and CCN2, to contribute cell growth in intestinal epithelial of colitis and CRC.

Table S1 The expression of 221 dysregulated circRNAs in GSE131911 dataset and 309 dysregulated circRNAs in GSE126094 and GSE138589 datasets.

circRNA ID	GSE131911			
	Fold change (UC/Healthy)	*P* value	Fold change (CD/Healthy)	*P* value
**Down-regulated circRNAs**				
hsa_circRNA_100634	0.346277367	0.0083974	0.396019348	0.0144354
hsa_circRNA_009139	0.164938489	0.00004721	0.157702799	0.0003644
hsa_circRNA_104705	0.585605091	0.0134234	0.617945102	0.0067368
hsa_circRNA_102690	0.616426163	0.00702937	0.634184851	0.0101949
hsa_circRNA_092392	0.285190929	0.00111256	0.229179196	0.0003496
hsa_circRNA_400620	0.601234624	0.01404771	0.639989548	0.0197124
hsa_circRNA_102709	0.450625231	0.02235485	0.224732916	0.0026596
hsa_circRNA_000963	0.38958229	0.01314442	0.247556954	0.0120392
hsa_circRNA_406503	0.604997045	0.00638376	0.592663083	0.0050193
hsa_circRNA_101456	0.194791145	0.00090845	0.113993582	0.0016875
hsa_circRNA_103611	0.568408487	0.01716316	0.624853397	0.0367971
hsa_circRNA_103889	0.186856156	0.00001431	0.206635621	0.0029277
hsa_circRNA_103137	0.184283652	0.00443057	0.152697739	0.0028306
hsa_circRNA_104050	0.540737382	0.0207256	0.588100809	0.0473434
hsa_circRNA_405520	0.460093825	0.00006237	0.561413978	0.0021434
hsa_circRNA_102704	0.341510064	0.00041038	0.278284328	0.0005003
hsa_circRNA_102446	0.06886907	0.00407262	0.077185607	0.0042074
hsa_circRNA_406237	0.125	0.01642552	0.111311219	0.0051343
hsa_circRNA_006603	0.214641359	0.00003923	0.228648232	0.0006238
hsa_circRNA_102442	0.299369676	0.02325209	0.266310416	0.0103772
hsa_circRNA_102286	0.463294031	0.01529891	0.523185731	0.0426082
hsa_circRNA_101765	0.307786103	0.0002204	0.353403913	0.0009688
hsa_circRNA_001130	0.243163737	0.01293887	0.284910388	0.0465789
hsa_circRNA_100332	0.635075491	0.03360401	0.618926415	0.0332074
hsa_circRNA_100650	0.583579051	0.01708101	0.593900993	0.0276381
hsa_circRNA_101958	0.378929142	0.0011426	0.277462736	0.0000661
hsa_circRNA_102807	0.496546248	0.00314495	0.665646203	0.043519
hsa_circRNA_036906	0.200267469	0.00007668	0.179630684	0.0002859
hsa_circRNA_400564	0.109575715	0.00002312	0.112502627	0.0002199
hsa_circRNA_100008	0.561749952	0.00604938	0.519807599	0.006815
hsa_circRNA_104652	0.140632311	0.00064403	0.252674987	0.0147215
hsa_circRNA_002904	0.138696184	0.00011344	0.12435873	0.0008191
hsa_circRNA_100498	0.386891248	0.01183162	0.350881673	0.0085702
hsa_circRNA_102408	0.142595464	0.00944036	0.124686644	0.0041545
hsa_circRNA_007809	0.537002236	0.03952728	0.477208568	0.0045068
hsa_circRNA_102616	0.666187413	0.04525521	0.621000063	0.0247384
hsa_circRNA_101762	0.406126198	0.00057916	0.450166979	0.0093307
hsa_circRNA_102305	0.236514412	0.00001162	0.239957468	0.0017827
hsa_circRNA_100395	0.188155843	0.01731158	0.211263058	0.0078279
hsa_circRNA_101206	0.135841858	0.0000802	0.147257191	0.0011411
hsa_circRNA_038011	0.153893052	0.00144604	0.119695949	0.0003772
hsa_circRNA_103269	0.346277367	0.00020549	0.492532749	0.0196132
hsa_circRNA_403959	0.630688704	0.00271437	0.643871738	0.0018733
hsa_circRNA_101489	0.339151082	0.00260085	0.335541585	0.0015306
hsa_circRNA_101458	0.194791145	0.00070018	0.11198214	0.0013226
hsa_circRNA_102059	0.538120062	0.00064185	0.655074334	0.0436884
hsa_circRNA_074491	0.089003137	0.0001188	0.101666793	0.0037683
hsa_circRNA_406283	0.139660892	0.00000259	0.150369722	0.001152
hsa_circRNA_024524	0.663882579	0.01382599	0.638300277	0.0225046
hsa_circRNA_400019	0.561749952	0.01534278	0.504251638	0.0001016
hsa_circRNA_405690	0.652477474	0.01172388	0.667605798	0.0076373
hsa_circRNA_008410	0.13490353	0.0009984	0.147132613	0.0037521
hsa_circRNA_104671	0.25348987	0.00258095	0.423292995	0.0285257
hsa_circRNA_405443	0.084202099	0.00557504	0.088658778	0.0170895
hsa_circRNA_104803	0.378929142	0.00066409	0.374048432	0.0001806
hsa_circRNA_102312	0.087777805	0.00016449	0.100975828	0.0032338
hsa_circRNA_401351	0.376311687	0.04088374	0.292693065	0.0416501
hsa_circRNA_404827	0.230046913	0.00040329	0.260059647	0.0032031
hsa_circRNA_403556	0.111105335	0.00087906	0.090460527	0.0028746
hsa_circRNA_005255	0.216134308	0.01095017	0.119083935	0.0004405
hsa_circRNA_400101	0.175555609	0.00027864	0.096320141	0.0010453
hsa_circRNA_102886	0.341510064	0.02934599	0.358434823	0.0333572
hsa_circRNA_085287	0.196146024	0.00535818	0.248857924	0.0404397
hsa_circRNA_103982	0.508387337	0.02048189	0.513471885	0.0288448
hsa_circRNA_101783	0.114228931	0.00002686	0.143138251	0.001294
hsa_circRNA_405485	0.38958229	0.00188877	0.392131371	0.0033766
hsa_circRNA_066781	0.615145672	0.04116792	0.524315231	0.012742
hsa_circRNA_074559	0.25	0.00171119	0.180686953	0.001522
hsa_circRNA_023461	0.10438599	0.00391132	0.10575374	0.0025018
hsa_circRNA_001264	0.23981603	0.00020396	0.1769691	0.0006668
hsa_circRNA_404879	0.314253344	0.01015153	0.360555466	0.0127268
hsa_circRNA_064335	0.615145672	0.00806721	0.611195379	0.002307
hsa_circRNA_007743	0.255253031	0.00009018	0.236324613	0.0002626
hsa_circRNA_103082	0.356012549	0.00004635	0.293735099	0.0001854
hsa_circRNA_000424	0.358488812	0.02062282	0.360790401	0.0429948

circRNA ID	GSE126094		GSE138589	
	Fold change (Tumor/Normal)	*P* value	Fold change (Tumor/Normal)	*P* value
**Down-regulated circRNAs**				
hsa_circRNA_000950	0.609289591	0.00019	0.518037015	0.0004447
hsa_circRNA_101004	0.198046348	1.95E-07	0.538605718	0.0006325
hsa_circRNA_100719	0.313715779	0.00922	0.366474482	0.0053591
**Up-regulated circRNAs**				
hsa_circRNA_104045	3.27267423	4.7E-08	1.737062821	0.0027893
hsa_circRNA_101900	3.075391333	0.00000276	2.051448433	0.0250169
hsa_circRNA_102025	1.734379969	0.0000238	2.094121879	0.0005755
hsa_circRNA_104651	2.870376532	0.0000132	2.373585634	0.01935
hsa_circRNA_104394	3.509997493	0.0000568	1.862880976	0.0244941
hsa_circRNA_101231	5.906854191	0.00000263	3.450875343	0.0007266
hsa_circRNA_104597	4.786496319	0.0000146	1.972414536	0.0095335
hsa_circRNA_101505	3.664864858	5.48E-08	3.64443927	0.0097215
hsa_circRNA_103341	1.774373839	0.000365	1.541230253	0.0291527
hsa_circRNA_103809	48.03419817	0.00000137	3.34035397	0.002639
hsa_circRNA_104810	2.269996385	0.000538	3.378761272	0.0045502
hsa_circRNA_103736	2.247467105	0.00032	1.976989502	0.0003269
hsa_circRNA_103038	3.509170389	0.00000384	2.476910981	0.0103231
hsa_circRNA_101056	2.331728799	0.000167	2.806696174	0.0146616
hsa_circRNA_103512	2.852347984	0.00000557	1.96175344	0.0122766
hsa_circRNA_100486	2.585117758	0.00000115	2.494421884	0.0002104
hsa_circRNA_102361	2.575316952	0.00000879	2.537930971	0.018249
hsa_circRNA_103113	2.010661192	0.0000759	1.77881016	0.0252425
hsa_circRNA_103140	4.230563971	2.78E-09	1.660864446	0.0185742
hsa_circRNA_102329	2.938826115	0.0000515	2.344955979	0.0025377
hsa_circRNA_100791	2.80243323	0.0000144	1.791222903	0.0067061
hsa_circRNA_104820	4.001969022	0.000134	2.939562964	0.0011917
hsa_circRNA_103489	2.084815912	0.00000687	3.173193107	0.0053496
hsa_circRNA_001914	2.307131123	4.3E-08	2.190115052	0.0032291
hsa_circRNA_104016	5.606498396	0.000197	2.814132641	0.0152271
hsa_circRNA_102488	2.527305382	8.51E-07	1.685203818	0.0123641
hsa_circRNA_103822	1.799929001	0.000732	1.711880801	0.0135738
hsa_circRNA_104170	3.491095929	0.0000234	2.360568536	0.0064046
hsa_circRNA_103680	2.282049365	0.0000219	2.434859017	0.0065424
hsa_circRNA_103737	2.191942571	0.000659	1.946850415	0.0003167
hsa_circRNA_101017	3.024527965	1.19E-09	1.612142118	0.0046811
hsa_circRNA_103450	2.0936641	8.64E-08	2.005487453	0.0028246
hsa_circRNA_102355	1.701963385	0.000113	2.057014638	0.001415
hsa_circRNA_100684	2.643794424	0.000193	1.755952209	0.0013998
hsa_circRNA_103127	2.176424539	0.000216	1.730718139	0.0074204
hsa_circRNA_101303	7.58752834	5.41E-09	3.602447686	0.0005346
hsa_circRNA_103012	2.651061245	0.000149	1.587984862	0.0191472
hsa_circRNA_101103	2.689130931	0.00000662	1.639394076	0.0031363
hsa_circRNA_101119	2.092546961	0.000741	2.186920431	0.0131847
hsa_circRNA_102960	3.139598336	0.0000272	2.189309939	0.0063588
hsa_circRNA_104522	2.449674391	0.0000672	2.280675909	0.0276998
hsa_circRNA_104477	2.60012323	0.00000608	1.904988264	0.0010627
hsa_circRNA_104869	2.517444548	0.000028	2.044203552	0.0477856
hsa_circRNA_101874	2.701498723	0.000222	2.381145072	0.0315029
hsa_circRNA_102539	2.843149576	9.23E-07	1.696454022	0.0103088
hsa_circRNA_104634	2.915230584	0.00000367	3.797550184	0.0113954
hsa_circRNA_104545	2.771448117	0.00000291	1.996775517	0.0150861
hsa_circRNA_104720	3.991801551	8.12E-09	1.611332962	0.0021968
hsa_circRNA_104575	3.515134762	0.00000291	3.027182954	0.0033511
hsa_circRNA_103493	2.461657418	0.0000281	4.428741158	0.0049588
hsa_circRNA_104999	2.110200379	0.0000276	2.192845153	0.0228426
hsa_circRNA_103987	4.171655432	2.67E-07	1.731456425	0.0247505
hsa_circRNA_101836	6.668397014	0.000518	2.394374202	0.0296765
hsa_circRNA_101835	7.542328567	0.000217	2.079166912	0.0115612
hsa_circRNA_103513	2.959882694	0.00000776	1.558658554	0.0327577
hsa_circRNA_001886	3.212819654	3.81E-07	1.910305334	0.0086314
hsa_circRNA_001826	3.024989218	0.000607	3.174068184	0.0298306
hsa_circRNA_104761	3.172477649	0.000435	2.992980377	0.0061982
hsa_circRNA_102746	2.866459708	0.000114	1.627180776	0.0148893
hsa_circRNA_103045	2.718479596	0.00171	2.818879212	0.0465875
hsa_circRNA_102829	2.971476559	0.0000616	1.96478241	0.0031787
hsa_circRNA_103827	4.147983053	0.00000514	2.089187591	0.0137216
hsa_circRNA_000581	2.018746838	0.000481	1.701983063	0.0288082
hsa_circRNA_101592	5.833129082	0.00000191	1.832261069	0.0166192
hsa_circRNA_104330	1.726535502	0.00144	1.789090913	0.0492776
hsa_circRNA_104692	3.722185614	0.0000044	3.603822711	0.0046842
hsa_circRNA_103762	1.950832619	0.0000074	2.055815265	0.0167386
hsa_circRNA_103292	3.08837941	0.00000332	1.861819754	0.0251055
hsa_circRNA_101905	2.83988006	0.000569	1.603585447	0.0383989
hsa_circRNA_100213	5.124921832	1.03E-08	2.03355841	0.0033834
hsa_circRNA_104851	2.686988227	0.0000063	2.591655611	0.0004865

hsa_circRNA_100891	0.149684838	0.00227195	0.129195572	0.0008596
hsa_circRNA_103399	0.577943353	0.01878111	0.406202093	0.0001433
hsa_circRNA_104121	0.135841858	0.00272636	0.122492597	0.0004195
hsa_circRNA_007494	0.154963462	0.00001033	0.163921766	0.0003581
**Up-regulated circRNAs**				
hsa_circRNA_067574	1.880348405	0.00023911	1.570641672	0.010169
hsa_circRNA_102723	1.705269784	0.00166815	1.814315733	0.0011762
hsa_circRNA_104497	1.586667686	0.00346737	1.562215121	0.0006084
hsa_circRNA_017969	1.663244197	0.00302044	1.632678998	0.002689
hsa_circRNA_004262	2.445280555	0.00007839	2.504454463	0.0010874
hsa_circRNA_102061	1.71356391	0.02641065	1.712510294	0.0093559
hsa_circRNA_104004	1.584469622	0.00791685	1.713261	0.0452996
hsa_circRNA_101767	1.515716567	0.00920494	1.577269612	0.0374742
hsa_circRNA_102053	1.549711862	0.04521385	2.032260173	0.0010779
hsa_circRNA_070294	1.731473131	0.00171188	1.72873061	0.0081435
hsa_circRNA_090069	2.29739671	0.00016443	1.919199371	0.0004092
hsa_circRNA_009541	2.099433367	0.00007222	1.678504634	0.0015672
hsa_circRNA_104871	1.815038311	0.01051624	1.811904868	0.0013637
hsa_circRNA_100853	1.6724928	0.00133375	1.50100506	0.0101315
hsa_circRNA_407325	2.265767771	0.00027227	2.010815451	0.0037345
hsa_circRNA_104974	1.699369998	0.00092078	2.117687718	0.0025619
hsa_circRNA_405011	1.584469622	0.00169265	1.518263755	0.0186342
hsa_circRNA_000595	1.884262548	0.000348	1.948729573	0.0014834
hsa_circRNA_400296	1.585568273	0.00898578	1.607426075	0.0318269
hsa_circRNA_008584	1.669018562	0.00448781	1.803385929	0.0098102
hsa_circRNA_062556	1.698192493	0.00306876	1.603853424	0.0126572
hsa_circRNA_400455	1.62113024	0.00376561	1.592987482	0.0130046
hsa_circRNA_074657	1.542210825	0.00534013	1.57794092	0.0117664
hsa_circRNA_007293	1.571345033	0.01039163	1.72937065	0.0127209
hsa_circRNA_405294	2.143546925	0.00048469	1.892011831	0.0068911
hsa_circRNA_407339	2.219138944	0.00005922	2.373145012	0.0001075
hsa_circRNA_038032	1.684125907	0.00881161	1.779920963	0.0079898
hsa_circRNA_100944	1.854461093	0.00019938	1.855037691	0.0008023
hsa_circRNA_105037	1.925188886	0.00117926	1.86286224	0.006826
hsa_circRNA_002361	1.525201653	0.01240161	1.528000907	0.0311864
hsa_circRNA_023216	2.203810232	0.00494132	1.923572185	0.003626
hsa_circRNA_059476	1.511519928	0.00625746	1.611791466	0.0021437
hsa_circRNA_000735	2.114036081	0.01295069	1.604347565	0.0147761
hsa_circRNA_103188	1.766630103	0.0389224	1.638314555	0.022291
hsa_circRNA_101110	1.771535038	0.00020889	1.546981281	0.0013871
hsa_circRNA_405010	1.876442393	0.00084523	1.603354545	0.0152448
**hsa_circRNA_104507**	**1.775222675**	**0.00402267**	**2.130825809**	**0.0038097**
hsa_circRNA_025249	1.795020101	0.0055942	1.637525792	0.0182662
hsa_circRNA_026634	1.93053405	0.00075055	2.090786298	0.0047628
hsa_circRNA_011164	1.6724928	0.00696949	1.54742747	0.0169097
hsa_circRNA_402103	1.5888688	0.00515061	1.556348158	0.0157438
hsa_circRNA_085323	2.808889751	0.00000521	2.589327393	0.0000953
hsa_circRNA_104839	1.543280175	0.00215429	1.50311129	0.0101547
hsa_circRNA_082275	2.056227653	0.00006493	1.684653257	0.0045042
hsa_circRNA_049237	1.528376521	0.00748072	1.699590341	0.0036956
hsa_circRNA_405080	1.64832417	0.00333104	1.504381833	0.043048
hsa_circRNA_400629	1.818816504	0.00107066	1.806297767	0.0150639
hsa_circRNA_102100	2.158456473	0.00010978	2.502767665	0.0007362
hsa_circRNA_003237	1.536875181	0.0003954	1.721018851	0.000096
hsa_circRNA_074530	1.937236378	0.00064917	1.662808226	0.0003449
hsa_circRNA_400856	1.758079122	0.00019082	1.651023643	0.000204
hsa_circRNA_100164	1.729074463	0.00146227	1.880407683	0.0063509
hsa_circRNA_039187	1.686462221	0.01077548	1.51749079	0.0024857
hsa_circRNA_104270	1.721898377	0.00685618	4.99060095	0.004629
hsa_circRNA_101076	1.823866331	0.00073297	2.014643095	0.0061479
hsa_circRNA_405551	1.573524891	0.00660324	2.007640479	0.0022818
hsa_circRNA_103047	1.889494082	0.01587987	1.740417466	0.0105223
hsa_circRNA_056734	1.568080908	0.0072312	1.520853691	0.0342437
hsa_circRNA_102219	1.890804234	0.00170144	1.976633847	0.0000799
hsa_circRNA_009024	3.363585661	0.01384074	4.803740806	0.0096864
hsa_circRNA_401091	1.961558008	0.00635383	1.628433934	0.0014381
hsa_circRNA_102999	1.888184838	0.0002937	1.63427301	0.0081491
hsa_circRNA_032154	1.581178233	0.03926527	1.682344171	0.0258413
hsa_circRNA_001498	1.62113024	0.00282626	1.607262466	0.0018792
hsa_circRNA_102303	1.688801775	0.00305053	1.615794528	0.0053537
hsa_circRNA_104873	1.505246747	0.00216428	1.658906253	0.0020906
hsa_circRNA_101707	1.825130977	0.00158912	1.978036736	0.0032933
hsa_circRNA_021120	1.692317193	0.0011368	1.529521217	0.0149849
hsa_circRNA_101768	1.667862088	0.00086256	1.582030309	0.0027518
hsa_circRNA_051036	2.084931522	0.00036536	1.930060458	0.0020452
hsa_circRNA_092417	1.840375301	0.00049623	1.959382676	0.0013093
hsa_circRNA_101887	1.689972769	0.00467928	1.502608127	0.0445277
hsa_circRNA_009000	1.858321349	0.00069341	1.635337716	0.0111408
hsa_circRNA_000850	1.810012926	0.00058453	1.684141351	0.0014728
hsa_circRNA_083182	2.128740365	0.0001644	2.414292041	0.0011633
hsa_circRNA_104852	13.42648726	0.0000124	7.266638968	0.001053
hsa_circRNA_102933	1.966281666	4.84E-07	1.687699651	0.0406483
hsa_circRNA_100487	2.176122844	0.00000123	2.071002249	0.000104
hsa_circRNA_103968	2.539562535	0.0000473	2.070059664	0.0365819
hsa_circRNA_103860	1.742960651	0.000861	1.635781429	0.0194922
hsa_circRNA_104411	4.766366867	0.000145	2.378013706	0.0075015
hsa_circRNA_104205	4.574360286	5.35E-08	1.802738464	0.0102851
hsa_circRNA_103978	2.805095717	0.00000669	2.504597076	0.0221118
hsa_circRNA_102447	3.201748528	3.36E-07	2.129445313	0.042728
hsa_circRNA_104804	2.491146731	0.000757	3.132685564	0.0391076
hsa_circRNA_102525	2.130541273	0.0000931	1.687650356	0.038326
hsa_circRNA_103112	2.127235856	0.00307	1.694496339	0.0447151
hsa_circRNA_104765	1.965954592	0.000769	2.178789938	0.0298006
hsa_circRNA_104700	15.80752943	2.96E-07	2.601393328	0.0136702
hsa_circRNA_103244	3.446519075	0.00000011	1.789918675	0.0248368
hsa_circRNA_102751	4.709976952	0.00000237	1.535835542	0.0253591
hsa_circRNA_104914	1.887896926	0.0000189	1.959535066	0.0202248
hsa_circRNA_100485	1.69313851	0.0000798	1.610128656	0.0009652
hsa_circRNA_101436	4.462678884	0.00003	2.670463323	0.0071519
hsa_circRNA_104758	4.194677802	0.00000411	2.002399797	0.0037859
hsa_circRNA_100380	2.186510081	0.000145	3.120376342	0.0087686
hsa_circRNA_101232	2.318111563	0.000235	2.371387043	0.0032797
hsa_circRNA_104774	2.254894601	0.00000693	1.686074203	0.0046376
hsa_circRNA_102742	2.530583362	0.0000794	1.726248858	0.0223164
hsa_circRNA_101444	1.889834634	0.00132	1.550732141	0.0324031
hsa_circRNA_102713	2.320683857	0.0000631	2.113932851	0.0074937
hsa_circRNA_104234	1.878212112	0.00117	1.620101341	0.0311914
hsa_circRNA_101554	2.677784757	0.00284	1.538224141	0.0483981
hsa_circRNA_100017	3.424278399	0.0000151	1.69531547	0.0046184
hsa_circRNA_104253	2.397980621	0.00000941	1.552039903	0.0406293
hsa_circRNA_102034	5.883277148	3.63E-07	1.903599407	0.0197927
hsa_circRNA_100933	2.208229319	0.000148	2.881341619	0.0106142
hsa_circRNA_101102	3.133532595	7.12E-07	4.493431689	0.015516
hsa_circRNA_102285	1.778018087	0.0000658	2.028337998	0.027116
hsa_circRNA_102737	1.687748574	0.0155	2.718458455	0.0303129
hsa_circRNA_104300	2.922574903	0.00000827	2.543216339	0.0024572
hsa_circRNA_100013	4.20186555	0.00000488	1.763407193	0.006021
hsa_circRNA_104834	2.201596375	0.00000977	4.696462826	0.0152198
hsa_circRNA_103552	1.733550663	0.0000888	4.773028994	0.0126622
hsa_circRNA_104329	2.162080141	0.0000659	1.675183979	0.0027507
hsa_circRNA_104779	1.796389258	0.000685	1.551859944	0.0232064
hsa_circRNA_103106	2.862905401	0.00000588	1.752107131	0.0260084
hsa_circRNA_104320	1.660445073	0.000419	1.655529215	0.0122067
hsa_circRNA_103681	2.518736149	0.0000644	1.84939256	0.0039493
hsa_circRNA_103654	2.19244401	0.00112	1.670907686	0.0235606
hsa_circRNA_103653	2.611104143	0.000638	1.682812629	0.0329549
hsa_circRNA_103462	2.142551675	0.00000189	1.662191288	0.003345
hsa_circRNA_100919	3.439860359	1.06E-08	1.647823931	0.0038775
hsa_circRNA_102476	3.094700941	0.00108	1.788926396	0.0329898
hsa_circRNA_101163	1.605163215	0.000204	1.551439071	0.0048655
hsa_circRNA_103836	1.835826857	0.000081	1.527663156	0.0177106
hsa_circRNA_103797	3.787799588	0.00000816	1.566873022	0.0200304
hsa_circRNA_104490	3.384001012	0.000165	3.074328587	0.0036583
hsa_circRNA_100324	1.909440546	0.0000168	1.822650834	0.0086741
hsa_circRNA_104326	4.747605303	4.58E-07	3.321852535	0.0054821
hsa_circRNA_104980	2.259134241	0.00459	1.681006951	0.0316257
hsa_circRNA_100241	2.212902672	0.000539	2.695914607	0.0255606
hsa_circRNA_102745	3.937527502	4.93E-07	1.789981975	0.0061669
hsa_circRNA_102328	2.983053806	0.0000338	3.686673362	0.0001977
hsa_circRNA_104031	2.733652765	0.0000254	1.641476914	0.0214241
hsa_circRNA_104523	1.723211759	0.000713	2.736727935	0.0034611
hsa_circRNA_103966	1.661354559	0.000184	1.667664815	0.0356168
hsa_circRNA_101854	2.487557717	0.0000116	2.381242073	0.009757
hsa_circRNA_104448	1.94054246	0.00131	2.584067525	0.0252429
hsa_circRNA_101513	3.367154687	0.0000115	1.982533151	0.0488381
hsa_circRNA_100543	2.059965249	0.0000579	2.260756059	0.0033086
hsa_circRNA_104853	2.095682264	0.00000691	1.945450537	0.0108904
hsa_circRNA_103572	4.518926212	0.000698	2.946849333	0.0156402
hsa_circRNA_102039	2.256692737	0.0000708	1.621068405	0.0464416
hsa_circRNA_103492	2.766093647	0.00000494	2.581282945	0.0032645
hsa_circRNA_102855	2.683507648	0.000187	1.901284992	0.0004274
hsa_circRNA_104821	4.166049554	0.000195	3.6116317	0.0034069
hsa_circRNA_104658	1.950697402	0.000884	2.046986844	0.0179926
hsa_circRNA_102712	2.631052434	0.0000106	2.640533674	0.0082508
hsa_circRNA_103792	1.835394259	0.00000991	2.637036555	0.0007318
hsa_circRNA_105012	1.787483593	0.000368	1.668100267	0.0094998
hsa_circRNA_104880	2.447179624	6.66E-08	1.541768321	0.0071352
hsa_circRNA_100786	1.720264018	0.000977	1.611257138	0.0268451
hsa_circRNA_101548	1.955801562	0.00243	2.003701667	0.0070387
hsa_circRNA_103888	2.59177414	0.00135	1.896333924	0.0306984

hsa_circRNA_404833	2.657371628	0.00068988	2.04728223	0.0000414
hsa_circRNA_049948	1.557249382	0.01677801	1.589497922	0.0193431
hsa_circRNA_102451	1.886876502	0.00947026	1.701416547	0.0030657
hsa_circRNA_023115	1.777685362	0.00252381	1.736199859	0.0089727
hsa_circRNA_000121	2.329467173	0.03561841	1.59491299	0.0163097
hsa_circRNA_404904	1.914542916	0.00013504	1.865327112	0.0001839
hsa_circRNA_400073	2.084931522	0.00019442	1.968642574	0.0017357
hsa_circRNA_405652	1.5888688	0.0027567	1.649522058	0.0020042
hsa_circRNA_081055	1.846764621	0.00288792	1.638033713	0.0329248
hsa_circRNA_057362	2.070529848	0.00014232	2.08010371	0.0010872
hsa_circRNA_102484	1.995845438	0.00053251	1.624722467	0.0045154
hsa_circRNA_000285	1.516767545	0.00607137	1.52781986	0.0120439
hsa_circRNA_001169	2.084931522	0.0003083	2.047547584	0.0003396
hsa_circRNA_007630	2.070529848	0.00028551	2.217091221	0.0000445
hsa_circRNA_405429	1.501079098	0.02000131	1.575235795	0.0315195
hsa_circRNA_104269	1.712376569	0.00335939	1.613584052	0.0326112
hsa_circRNA_103594	1.689972769	0.0004717	1.609693352	0.0016688
hsa_circRNA_008059	1.741101127	0.00094815	1.99059836	0.0007339
hsa_circRNA_022019	1.911890635	0.00004476	1.903792007	0.0007725
hsa_circRNA_006173	1.863480859	0.00016207	2.189213806	0.0000733
hsa_circRNA_092373	1.555092072	0.00728136	1.622072237	0.0128604
hsa_circRNA_040773	1.662091723	0.00222059	2.088515079	0.0008717
hsa_circRNA_000536	2.0139111	0.00034652	1.837550718	0.0022373
hsa_circRNA_043626	1.691144575	0.00092503	1.683602748	0.0010401
hsa_circRNA_405716	2.173469725	0.00013243	2.266050009	0.0025785
hsa_circRNA_102735	1.792533399	0.00018135	1.647108634	0.0006229
hsa_circRNA_037936	1.629015126	0.00477649	2.037679398	0.0023364
hsa_circRNA_105050	2.128740365	0.02094973	3.083193596	0.0294718
hsa_circRNA_406748	2.250116969	0.00046108	2.053692382	0.0001373
hsa_circRNA_104973	1.671333918	0.00184187	1.915468711	0.005913
hsa_circRNA_404839	1.705269784	0.00582489	1.70953346	0.0015491
hsa_circRNA_103910	1.656341323	0.00363318	1.667896967	0.0070645
hsa_circRNA_031809	1.65175533	0.0031508	1.72607806	0.0130791
hsa_circRNA_072697	1.622254311	0.00370478	1.610434797	0.0384853
hsa_circRNA_407279	1.869950396	0.0017268	1.798640999	0.0037191
hsa_circRNA_102805	1.547564994	0.00216709	1.614663584	0.0016286
hsa_circRNA_401587	1.630144665	0.00449113	1.515434524	0.0066983
hsa_circRNA_407024	1.715941061	0.0044039	1.754991776	0.0104105
hsa_circRNA_101115	1.643760375	0.00139064	2.184643268	0.0003237
hsa_circRNA_102066	1.832737289	0.00084684	1.762647502	0.0009174
hsa_circRNA_407005	1.76418273	0.01179031	1.733784608	0.0174296
hsa_circRNA_067718	1.516767545	0.0059591	1.785340622	0.0005416
hsa_circRNA_086475	1.56265576	0.00535021	1.593404716	0.0071438
hsa_circRNA_104095	1.998614186	0.00005944	1.518882218	0.0200947
hsa_circRNA_032475	1.577893682	0.00570264	1.615537826	0.0056003
hsa_circRNA_406705	2.0139111	0.00013561	1.872875306	0.001544
hsa_circRNA_000459	1.80000386	0.00163343	2.004650635	0.0023294
hsa_circRNA_008077	1.712376569	0.00094229	1.775499618	0.000217
hsa_circRNA_045871	1.554014538	0.01001283	1.729277849	0.0051981
hsa_circRNA_400862	2.114036081	0.02453067	1.525047787	0.0051434
hsa_circRNA_101132	1.686462221	0.00047351	1.555158192	0.0144604
hsa_circRNA_000100	1.548638056	0.00141216	1.657134382	0.0011979
hsa_circRNA_100027	1.656341323	0.00123936	1.658347144	0.0045593
hsa_circRNA_007586	1.630144665	0.0103144	1.531810805	0.0036401
hsa_circRNA_406934	1.605474777	0.00119476	1.75197941	0.0009287
hsa_circRNA_100445	1.886876502	0.00079552	1.916763406	0.0158496
hsa_circRNA_104341	1.733875127	0.00846572	1.568308893	0.0104278
hsa_circRNA_092516	1.701727459	0.00162184	1.740427491	0.0038858
hsa_circRNA_403515	1.5888688	0.00372635	1.626193496	0.0056305
hsa_circRNA_100621	1.835279765	0.00033343	1.727059264	0.0060299
hsa_circRNA_004092	1.601029621	0.03099225	1.541579819	0.0464746
hsa_circRNA_100073	1.615521555	0.00368028	1.580505187	0.0130885
hsa_circRNA_076039	1.511519928	0.00405066	1.775423367	0.0018485
hsa_circRNA_051348	1.868654694	0.00048088	1.588062035	0.0068833
hsa_circRNA_402266	1.574615953	0.00083321	1.554224674	0.0004263
hsa_circRNA_008068	1.946658748	0.01351834	1.989190547	0.001752
hsa_circRNA_104096	1.926523788	0.00181523	1.543924174	0.0432793

hsa_circRNA_101260	2.152062534	0.000958	1.746543978	0.047328
hsa_circRNA_100949	2.185570627	0.00038	1.816095979	0.0327488
hsa_circRNA_103668	3.552882557	0.000391	1.895233362	0.044462
hsa_circRNA_104233	2.032267061	0.000113	2.473575972	0.0078374
hsa_circRNA_104868	1.872765159	0.00114	1.973345103	0.0125764
hsa_circRNA_103775	3.009283443	0.0000136	2.412506367	0.0108173
hsa_circRNA_101348	2.188238517	0.00000688	1.622698129	0.0049861
hsa_circRNA_100355	3.969645014	0.00042	3.035689932	0.0265787
hsa_circRNA_104775	2.138693882	0.0000447	1.756411493	0.001393
hsa_circRNA_000166	22.99964997	0.00000174	2.575315078	0.0364639
hsa_circRNA_101269	1.725183712	0.00675	2.170990255	0.0471613
hsa_circRNA_103682	3.170059678	0.000085	1.966700782	0.012002
hsa_circRNA_104168	3.519693982	4.37E-07	2.474363351	0.0040884
hsa_circRNA_100479	2.077315	0.0000015	2.87414136	0.0100177
hsa_circRNA_103829	4.779600379	0.00000357	2.803678609	0.0269353
hsa_circRNA_000585	8.025937937	0.00003	3.159457699	0.0113909
hsa_circRNA_103868	1.862589822	0.000354	1.823652124	0.0312542
hsa_circRNA_101445	2.19687076	0.0000709	1.888154488	0.0090254
hsa_circRNA_103359	3.30761198	0.00000557	1.668128538	0.0107675
hsa_circRNA_104467	3.026771991	1.22E-07	1.836811131	0.0112795
hsa_circRNA_001535	2.476444004	0.0000164	2.413850857	0.038374
hsa_circRNA_100352	2.443840281	0.0205	2.050868471	0.0085862
hsa_circRNA_000368	1.536353282	0.00082	1.673693275	0.0217321
hsa_circRNA_100536	2.640882292	0.0000282	1.870013843	0.0014143
hsa_circRNA_101262	3.023312272	0.0000447	2.473940118	0.0152196
hsa_circRNA_104677	2.169661533	0.0000116	1.832696918	0.0218238
hsa_circRNA_101638	2.029437626	0.0000782	2.06018378	0.0017493
hsa_circRNA_100381	1.837507324	0.000235	2.58183145	0.0061146
hsa_circRNA_102790	1.934324715	0.000324	1.610482015	0.0058524
hsa_circRNA_104822	2.677877563	0.00000364	1.876266007	0.0125586
hsa_circRNA_100161	1.809247778	0.00311	1.568992396	0.0133264
hsa_circRNA_102995	1.87457039	0.000637	1.826356901	0.0320177
hsa_circRNA_104532	2.956499432	0.00605	2.929424322	0.0352907
hsa_circRNA_104913	1.910870487	0.0000368	2.229067709	0.0381249
hsa_circRNA_103662	1.737628896	0.0000746	1.619052357	0.0006216
hsa_circRNA_104854	7.785663543	0.0000024	5.050474998	0.0048877
hsa_circRNA_103017	5.848594637	0.000189	2.964978164	0.0377764
hsa_circRNA_104812	1.908474619	0.0000422	4.475489085	0.0007333
hsa_circRNA_102736	2.217877987	0.00135	3.070822407	0.0231554
hsa_circRNA_103343	2.764847676	0.0000608	2.07905753	0.0252532
hsa_circRNA_104419	2.555011881	0.000105	2.528833707	0.02363
hsa_circRNA_102524	2.139227624	0.0000563	1.80862436	0.0360295
hsa_circRNA_100146	15.16916744	4.15E-07	1.636360253	0.0132235
hsa_circRNA_104435	5.476218458	0.000123	2.177125524	0.0292776
hsa_circRNA_100313	1.645116782	0.0455	1.86285355	0.0459615
hsa_circRNA_102097	2.437479356	0.0000123	2.584292036	0.0487603
hsa_circRNA_104764	3.194455406	0.000391	4.469109737	0.0069607
hsa_circRNA_100359	2.49392832	0.0000433	2.017325717	0.0041327
hsa_circRNA_100019	2.029733054	0.000138	1.638832547	0.0302311
hsa_circRNA_102392	2.52369926	0.00000562	1.577450408	0.0448158
hsa_circRNA_104909	1.778239938	0.0000761	1.937074337	0.0170788
hsa_circRNA_104689	3.279668503	0.000206	1.908929536	0.0205177
hsa_circRNA_102672	1.977557989	0.000159	1.596856808	0.0110175
hsa_circRNA_101572	2.555100433	0.0000204	1.978037134	0.0311692
hsa_circRNA_103973	2.764771019	0.00000144	1.640314203	0.0100794
hsa_circRNA_102854	1.938445266	0.000406	1.593242258	0.0027024
hsa_circRNA_100261	1.707469727	0.000505	1.878362055	0.0137764
hsa_circRNA_101903	6.619902644	0.00005	1.782433015	0.0171892
hsa_circRNA_101485	2.206485092	0.0000128	1.60127545	0.0147649
hsa_circRNA_101019	2.260716366	0.000425	2.170555858	0.0139513
hsa_circRNA_103571	3.38592496	0.000223	1.876107063	0.0381241
hsa_circRNA_104142	2.150526636	0.000063	1.541546139	0.045521
hsa_circRNA_103046	1.722769873	0.000518	1.831370273	0.0245022
hsa_circRNA_100363	1.842021627	0.00234	2.098150157	0.0133472
hsa_circRNA_104699	2.152569771	0.00000625	1.549832271	0.0254381
hsa_circRNA_101314	4.489827446	0.00000182	3.004352617	0.0161521
hsa_circRNA_100597	1.912632903	0.0000951	2.020070733	0.0162699
hsa_circRNA_100490	2.296982714	0.00000366	2.050216281	0.0037608
hsa_circRNA_102962	4.644746952	0.000024	2.231372958	0.0432064
hsa_circRNA_102503	1.864139725	0.000038	2.229986227	0.0034477
hsa_circRNA_103411	2.227398977	0.000325	1.531541256	0.0431758
hsa_circRNA_104220	1.846470227	0.000128	2.659100105	0.0068983
hsa_circRNA_100318	2.15661702	0.0027	1.576381267	0.0316608
hsa_circRNA_102395	5.332898981	9.08E-09	1.568908037	0.0123767
hsa_circRNA_100546	2.540795038	0.000372	2.412440265	0.0036242
hsa_circRNA_103219	3.350438673	4.23E-08	1.94337991	0.0073747
hsa_circRNA_104733	1.82010288	0.00167	2.302146589	0.0124521
hsa_circRNA_102973	2.093272308	0.00000613	1.589345203	0.0355163
hsa_circRNA_101085	2.937054426	0.0000119	3.227538824	0.045743
hsa_circRNA_103517	2.053877305	0.00128	1.548555103	0.0215314

hsa_circRNA_103803	1.76742623	0.00000228	1.952232107	0.0018962
hsa_circRNA_101514	3.422783403	0.0000145	2.024715831	0.0381474
hsa_circRNA_104236	1.765185743	0.00088	1.702249949	0.0085707
hsa_circRNA_104533	1.72476523	0.000739	1.668069997	0.0302116
hsa_circRNA_103670	2.191745066	0.0000286	2.099601408	0.0020999
hsa_circRNA_102387	1.77348853	0.00244	1.774146199	0.0437487
hsa_circRNA_101637	2.611212738	0.000297	2.917979929	0.0058831
hsa_circRNA_100491	2.500280576	1.92E-07	1.799741532	0.0005566
hsa_circRNA_000556	1.926296789	0.000344	2.07874926	0.0168901
hsa_circRNA_100537	2.880281863	0.0000362	1.567305915	0.0036753
hsa_circRNA_101462	2.639198751	0.00151	1.997053954	0.0215698
hsa_circRNA_100415	1.946645255	0.0000611	1.518137843	0.0053393
hsa_circRNA_103586	2.935955293	0.0000458	1.800527819	0.0123105
hsa_circRNA_103821	1.691449377	0.000666	1.619522992	0.0465374
hsa_circRNA_104591	2.250054584	0.000302	1.806945634	0.0080325
**hsa_circRNA_104507**	**4.124302065**	**0.00061**	**3.008846672**	**0.0079439**
hsa_circRNA_103364	1.792235226	0.000213	1.642254297	0.0070684
hsa_circRNA_103312	2.603856603	0.000415	2.600518137	0.0067082
hsa_circRNA_104018	1.817493242	0.000332	1.533541693	0.0110353
hsa_circRNA_104949	2.066013956	0.000115	3.018883953	0.0222172
hsa_circRNA_101172	1.870883857	0.000154	1.562382776	0.0244663
hsa_circRNA_103114	7.497553959	0.0000863	2.855834667	0.0215871
hsa_circRNA_104581	1.858308468	0.000171	1.945567143	0.012199
hsa_circRNA_104645	3.853688604	0.00249	2.69397647	0.035352
hsa_circRNA_100248	2.075041227	0.00000504	1.59378958	0.0073141
hsa_circRNA_104776	1.958962798	0.00249	2.067655324	0.0035542
hsa_circRNA_101965	3.831422171	0.00000063	1.916139278	0.0237626
hsa_circRNA_103511	2.880042298	0.0000161	1.710128211	0.0143813
hsa_circRNA_101981	2.866201425	0.000105	1.75149806	0.0013412
hsa_circRNA_103340	2.405822158	0.000378	1.736684155	0.0374551
hsa_circRNA_101484	2.190575592	0.0000176	1.574105067	0.0237026
hsa_circRNA_103344	2.459678916	0.000795	2.000250935	0.0086199
hsa_circRNA_102474	3.202858362	3.05E-07	1.557484159	0.0005119
hsa_circRNA_102521	2.249430823	0.0000297	1.836369811	0.0322805
hsa_circRNA_101241	2.657997965	0.000115	2.079711356	0.035856
hsa_circRNA_100526	2.30336014	0.0000127	1.976279462	0.0012426
hsa_circRNA_102826	2.302147068	0.000062	1.571625128	0.0310657
hsa_circRNA_104141	2.518037904	0.0000518	2.041520961	0.0367775
hsa_circRNA_101101	1.889546471	0.0000618	3.720969435	0.0109561
hsa_circRNA_101016	1.846598219	0.000097	1.573708115	0.0459014
hsa_circRNA_100638	2.421129085	0.000431	1.650129912	0.030871
hsa_circRNA_101648	2.166610774	0.000113	2.109906708	0.0089991
hsa_circRNA_103570	3.315347281	0.000427	1.935364092	0.0391108
hsa_circRNA_101736	2.278161462	0.000123	1.793720407	0.0065363
hsa_circRNA_101068	3.659356571	0.000122	2.002929623	0.0264031
hsa_circRNA_104417	2.114724902	0.0000682	2.576865701	0.0013471
hsa_circRNA_103124	3.463594412	0.00025	1.763857375	0.0230828
hsa_circRNA_103757	4.266934605	0.0000495	2.111472343	0.0043375
hsa_circRNA_101573	2.055614879	0.0000461	1.592882688	0.0319711
hsa_circRNA_101367	4.2594584	0.00000153	2.049752329	0.0201427
hsa_circRNA_104392	1.718381063	0.0000399	1.801235579	0.0279667
hsa_circRNA_100915	2.294595735	0.00000256	1.656263151	0.0219692
hsa_circRNA_102517	2.609222546	0.0000649	2.740222573	0.0189654
hsa_circRNA_102974	2.486712981	0.000317	3.134602552	0.0142313
hsa_circRNA_102856	3.331126173	0.000109	2.413991423	0.0014149
hsa_circRNA_102209	2.871530728	0.00298	1.837444826	0.0373243
hsa_circRNA_104517	1.912460565	0.0000014	1.561808836	0.020707
hsa_circRNA_104850	2.441876099	0.0000808	2.34321998	0.0013959
hsa_circRNA_100243	2.093533495	0.00125	2.04323855	0.043959
hsa_circRNA_001671	3.54847711	0.0000174	1.79702642	0.0141825
hsa_circRNA_104809	1.898671081	0.000504	1.685967002	0.0407394
hsa_circRNA_100977	2.294214048	0.00264	1.908401863	0.0198754
hsa_circRNA_101053	2.332569391	0.00000349	1.882047787	0.0029674
hsa_circRNA_103830	3.907754948	1.1E-08	1.787386509	0.0008951
hsa_circRNA_102794	1.866040114	0.0000818	1.529577747	0.0059723
hsa_circRNA_100364	1.766826039	0.00715	2.388641264	0.0122557
hsa_circRNA_103488	2.226087035	0.00000612	1.649857074	0.0473924
hsa_circRNA_102893	1.740208294	0.000285	2.134259883	0.008548
hsa_circRNA_103595	2.357013464	6.52E-07	1.843904248	0.0013233
hsa_circRNA_103412	2.099928199	0.000773	1.874906628	0.0176906
hsa_circRNA_101974	2.278114089	5.22E-08	1.860341089	0.0048315
hsa_circRNA_100794	1.895699117	0.000281	1.521559267	0.0271932
hsa_circRNA_104911	2.862925245	0.0000115	2.807081887	0.0317581
hsa_circRNA_101837	5.729026456	0.000168	1.959289442	0.0116672
hsa_circRNA_102927	3.131925727	0.000196	1.691380921	0.0229555

Table S2 Differentially expressed genes in circHIPK2 knockdown HCT116 cells and control cells.

Gene name	Description	Relative count (FPKM)		Fold-change (sicircHIPK2/siNC)	*P* value	*q* value	Regulation
		siNC	sicircHIPK2				
TRIB3	tribbles pseudokinase 3 [Source:HGNC Symbol;Acc:HGNC:16228]	5.40	25.45	4.71	0.00	0.00	up
LONRF2	LON peptidase N-terminal domain and ring finger 2 [Source:HGNC Symbol;Acc:HGNC:24788]	1.02	4.09	3.99	0.00	0.00	up
C19orf71	chromosome 19 open reading frame 71 [Source:HGNC Symbol;Acc:HGNC:34496]	0.00	2.34	23355.89	0.00	0.00	up
SCG2	secretogranin II [Source:HGNC Symbol;Acc:HGNC:10575]	9.45	29.96	3.17	0.00	0.00	up
ARRDC4	arrestin domain containing 4 [Source:HGNC Symbol;Acc:HGNC:28087]	18.24	51.77	2.84	0.00	0.00	up
CHAC1	ChaC glutathione specific gamma-glutamylcyclotransferase 1 [Source:HGNC Symbol;Acc:HGNC:2	3.13	13.82	4.41	0.00	0.00	up
TSC22D1	TSC22 domain family member 1 [Source:HGNC Symbol;Acc:HGNC:16826]	21.86	57.63	2.64	0.00	0.00	up
PCK2	phosphoenolpyruvate carboxykinase 2, mitochondrial [Source:HGNC Symbol;Acc:HGNC:8725]	3.98	13.04	3.28	0.00	0.00	up
MAPK14	mitogen-activated protein kinase 14 [Source:HGNC Symbol;Acc:HGNC:6876]	2.18	6.50	2.98	0.00	0.00	up
HABP4	hyaluronan binding protein 4 [Source:HGNC Symbol;Acc:HGNC:17062]	10.80	27.66	2.56	0.00	0.00	up
PSAT1	phosphoserine aminotransferase 1 [Source:HGNC Symbol;Acc:HGNC:19129]	29.47	69.92	2.37	0.00	0.00	up
ASNS	asparagine synthetase (glutamine-hydrolyzing) [Source:HGNC Symbol;Acc:HGNC:753]	19.16	46.41	2.42	0.00	0.00	up
ANKRD11	ankyrin repeat domain 11 [Source:HGNC Symbol;Acc:HGNC:21316]	2.75	6.47	2.35	0.00	0.00	up
CRCP	CGRP receptor component [Source:HGNC Symbol;Acc:HGNC:17888]	1.04	4.89	4.68	0.00	0.00	up
NES	nestin [Source:HGNC Symbol;Acc:HGNC:7756]	5.22	11.83	2.27	0.00	0.00	up
GAS6-AS1	GAS6 antisense RNA 1 [Source:HGNC Symbol;Acc:HGNC:39826]	3.30	8.23	2.49	0.00	0.00	up
TARID	TCF21 antisense RNA inducing promoter demethylation [Source:HGNC Symbol;Acc:HGNC:50506	2.19	5.80	2.65	0.00	0.00	up
ZNF629	zinc finger protein 629 [Source:HGNC Symbol;Acc:HGNC:29008]	1.80	4.82	2.67	0.00	0.00	up
JADE2	jade family PHD finger 2 [Source:HGNC Symbol;Acc:HGNC:22984]	0.61	1.94	3.18	0.00	0.00	up
DLGAP1-AS2	DLGAP1 antisense RNA 2 [Source:HGNC Symbol;Acc:HGNC:28146]	6.33	14.69	2.32	0.00	0.00	up
KLHDC7B	kelch domain containing 7B [Source:HGNC Symbol;Acc:HGNC:25145]	0.01	0.23	20.67	0.00	0.00	up
LINC01963	long intergenic non-protein coding RNA 1963 [Source:HGNC Symbol;Acc:HGNC:25283]	1.76	5.61	3.19	0.00	0.00	up
AC011473	novel transcript	0.02	0.62	27.86	0.00	0.00	up
ZNF48	zinc finger protein 48 [Source:HGNC Symbol;Acc:HGNC:13114]	0.71	3.38	4.74	0.00	0.00	up
SLC7A11	solute carrier family 7 member 11 [Source:HGNC Symbol;Acc:HGNC:11059]	2.24	5.52	2.46	0.00	0.00	up
KLF11	Kruppel like factor 11 [Source:HGNC Symbol;Acc:HGNC:11811]	2.67	6.91	2.59	0.00	0.00	up
TPM4	tropomyosin 4 [Source:HGNC Symbol;Acc:HGNC:12013]	19.04	41.92	2.20	0.00	0.00	up
APOBEC3H	apolipoprotein B mRNA editing enzyme catalytic subunit 3H [Source:HGNC Symbol;Acc:HGNC:2	2.29	9.38	4.09	0.00	0.00	up
ULBP1	UL16 binding protein 1 [Source:HGNC Symbol;Acc:HGNC:14893]	4.58	10.88	2.37	0.00	0.00	up
SESN2	sestrin 2 [Source:HGNC Symbol;Acc:HGNC:20746]	8.79	18.20	2.07	0.00	0.00	up
ZNF204P	zinc finger protein 204, pseudogene [Source:HGNC Symbol;Acc:HGNC:12995]	5.61	13.15	2.34	0.00	0.00	up
MRPL49	mitochondrial ribosomal protein L49 [Source:HGNC Symbol;Acc:HGNC:1176]	5.11	19.93	3.90	0.00	0.00	up
HMOX1	heme oxygenase 1 [Source:HGNC Symbol;Acc:HGNC:5013]	11.73	25.69	2.19	0.00	0.00	up
ZNF827	zinc finger protein 827 [Source:HGNC Symbol;Acc:HGNC:27193]	1.13	3.25	2.87	0.00	0.00	up
AC112229	novel transcript	0.00	0.23	180.19	0.00	0.00	up
TMEM198B	transmembrane protein 198B (pseudogene) [Source:HGNC Symbol;Acc:HGNC:43629]	2.24	5.84	2.60	0.00	0.00	up
SLC16A13	solute carrier family 16 member 13 [Source:HGNC Symbol;Acc:HGNC:31037]	1.11	3.97	3.58	0.00	0.00	up
STX12	syntaxin 12 [Source:HGNC Symbol;Acc:HGNC:11430]	7.52	15.25	2.03	0.00	0.00	up
MT-CYB	mitochondrially encoded cytochrome b [Source:HGNC Symbol;Acc:HGNC:7427]	2093.03	4478.69	2.14	0.00	0.00	up
ZCCHC3	zinc finger CCHC-type containing 3 [Source:HGNC Symbol;Acc:HGNC:16230]	5.52	11.82	2.14	0.00	0.00	up
C22orf46	chromosome 22 open reading frame 46 [Source:HGNC Symbol;Acc:HGNC:26294]	2.83	6.13	2.17	0.00	0.00	up
UBLCP1	ubiquitin like domain containing CTD phosphatase 1 [Source:HGNC Symbol;Acc:HGNC:28110]	6.43	14.18	2.21	0.00	0.00	up
LINC00886	long intergenic non-protein coding RNA 886 [Source:HGNC Symbol;Acc:HGNC:48572]	0.76	2.90	3.83	0.00	0.00	up
FGF19	fibroblast growth factor 19 [Source:HGNC Symbol;Acc:HGNC:3675]	13.33	27.14	2.04	0.00	0.00	up
COL17A1	collagen type XVII alpha 1 chain [Source:HGNC Symbol;Acc:HGNC:2194]	0.55	1.66	3.00	0.00	0.00	up
CLEC18B	C-type lectin domain family 18 member B [Source:HGNC Symbol;Acc:HGNC:33849]	0.16	0.98	6.21	0.00	0.00	up
LINC00326	long intergenic non-protein coding RNA 326 [Source:HGNC Symbol;Acc:HGNC:41926]	4.89	11.53	2.36	0.00	0.00	up
ZBED3	zinc finger BED-type containing 3 [Source:HGNC Symbol;Acc:HGNC:20711]	2.07	4.68	2.25	0.00	0.00	up
CCDC18-AS1	CCDC18 antisense RNA 1 [Source:HGNC Symbol;Acc:HGNC:52262]	1.32	3.73	2.83	0.00	0.00	up
STX16-NPEPL1	STX16-NPEPL1 readthrough (NMD candidate) [Source:HGNC Symbol;Acc:HGNC:41993]	0.03	0.89	27.89	0.00	0.00	up
ZNF425	zinc finger protein 425 [Source:HGNC Symbol;Acc:HGNC:20690]	0.66	2.27	3.45	0.00	0.00	up
RPPH1	ribonuclease P RNA component H1 [Source:HGNC Symbol;Acc:HGNC:19273]	1.45	17.22	11.89	0.00	0.00	up
DSE	dermatan sulfate epimerase [Source:HGNC Symbol;Acc:HGNC:21144]	1.11	2.23	2.01	0.00	0.00	up
VAMP7	vesicle associated membrane protein 7 [Source:HGNC Symbol;Acc:HGNC:11486]	5.36	14.50	2.71	0.00	0.00	up
SLC1A4	solute carrier family 1 member 4 [Source:HGNC Symbol;Acc:HGNC:10942]	0.42	1.30	3.13	0.00	0.00	up
HIST1H2BD	histone cluster 1 H2B family member d [Source:HGNC Symbol;Acc:HGNC:4747]	24.72	51.12	2.07	0.00	0.00	up
TOMM6	translocase of outer mitochondrial membrane 6 [Source:HGNC Symbol;Acc:HGNC:34528]	2.26	78.13	34.57	0.00	0.00	up
FAM102A	family with sequence similarity 102 member A [Source:HGNC Symbol;Acc:HGNC:31419]	6.65	13.98	2.10	0.00	0.00	up
KRCC1	lysine rich coiled-coil 1 [Source:HGNC Symbol;Acc:HGNC:28039]	1.36	4.48	3.29	0.00	0.00	up
AC011448	readthrough between NDUFA13 and YJEFN3	1.01	10.83	10.67	0.00	0.00	up
AL512306	novel transcript	0.28	1.42	5.10	0.00	0.00	up
AL136982	ArfGAP with GTPase domain, ankyrin repeat and PH domain family (AGAP) pseudogene	0.00	0.77	7735.61	0.00	0.00	up
MESD	mesoderm development LRP chaperone [Source:HGNC Symbol;Acc:HGNC:13520]	2.93	6.09	2.08	0.00	0.00	up
RESF1	retroelement silencing factor 1 [Source:HGNC Symbol;Acc:HGNC:25559]	2.63	6.40	2.44	0.00	0.00	up
ANKRD13B	ankyrin repeat domain 13B [Source:HGNC Symbol;Acc:HGNC:26363]	3.65	7.74	2.12	0.00	0.00	up
KLF2	Kruppel like factor 2 [Source:HGNC Symbol;Acc:HGNC:6347]	26.35	53.44	2.03	0.00	0.00	up
FCGBP	Fc fragment of IgG binding protein [Source:HGNC Symbol;Acc:HGNC:13572]	0.47	1.23	2.60	0.00	0.00	up
TCTA	T cell leukemia translocation altered [Source:HGNC Symbol;Acc:HGNC:11692]	2.87	8.82	3.07	0.00	0.00	up
DDX11L2	DEAD/H-box helicase 11 like 2 [Source:HGNC Symbol;Acc:HGNC:37103]	1.62	5.53	3.41	0.00	0.00	up
FGF9	fibroblast growth factor 9 [Source:HGNC Symbol;Acc:HGNC:3687]	1.82	4.16	2.29	0.00	0.00	up
PSKH1	protein serine kinase H1 [Source:HGNC Symbol;Acc:HGNC:9529]	1.59	4.13	2.59	0.00	0.00	up
TYMP	thymidine phosphorylase [Source:HGNC Symbol;Acc:HGNC:3148]	4.44	9.79	2.20	0.00	0.00	up
ZNF616	zinc finger protein 616 [Source:HGNC Symbol;Acc:HGNC:28062]	0.71	2.05	2.87	0.00	0.00	up
AP002478	novel transcript, antisense to DLGAP1	3.97	10.11	2.55	0.00	0.00	up
RAB43	RAB43, member RAS oncogene family [Source:HGNC Symbol;Acc:HGNC:19983]	0.25	1.37	5.43	0.00	0.00	up
C10orf91	chromosome 10 open reading frame 91 (putative) [Source:HGNC Symbol;Acc:HGNC:27275]	0.16	0.90	5.68	0.00	0.00	up
C6orf141	chromosome 6 open reading frame 141 [Source:HGNC Symbol;Acc:HGNC:21351]	0.97	2.83	2.91	0.00	0.00	up
NCOA5	nuclear receptor coactivator 5 [Source:HGNC Symbol;Acc:HGNC:15909]	3.12	6.83	2.19	0.00	0.00	up
MCRIP2P1	MAPK regulated corepressor interacting protein 2 pseudogene 1 [Source:HGNC Symbol;Acc:HGN	0.07	1.31	17.63	0.00	0.00	up
ZNF587B	zinc finger protein 587B [Source:HGNC Symbol;Acc:HGNC:37142]	0.55	1.63	2.94	0.00	0.00	up
AC068888	novel transcript, antisense to TENC1 & EIF4B	0.88	2.33	2.66	0.00	0.00	up
LINC01106	long intergenic non-protein coding RNA 1106 [Source:HGNC Symbol;Acc:HGNC:26769]	5.35	11.59	2.17	0.00	0.00	up
RMRP	RNA component of mitochondrial RNA processing endoribonuclease [Source:HGNC Symbol;Acc:H	1.29	17.39	13.51	0.00	0.00	up
C1RL	complement C1r subcomponent like [Source:HGNC Symbol;Acc:HGNC:21265]	2.03	4.30	2.12	0.00	0.00	up
AL024497	novel transcript, antisense to EYA4	5.31	14.16	2.67	0.00	0.00	up
WNT9A	Wnt family member 9A [Source:HGNC Symbol;Acc:HGNC:12778]	2.44	5.20	2.13	0.00	0.00	up
RNF170	ring finger protein 170 [Source:HGNC Symbol;Acc:HGNC:25358]	1.65	3.43	2.08	0.00	0.00	up
CYP4V2	cytochrome P450 family 4 subfamily V member 2 [Source:HGNC Symbol;Acc:HGNC:23198]	0.83	1.84	2.22	0.00	0.00	up
ADPRHL1	ADP-ribosylhydrolase like 1 [Source:HGNC Symbol;Acc:HGNC:21303]	1.02	2.06	2.03	0.00	0.00	up
TSC22D3	TSC22 domain family member 3 [Source:HGNC Symbol;Acc:HGNC:3051]	3.25	7.91	2.44	0.00	0.00	up
ABCF2	ATP binding cassette subfamily F member 2 [Source:HGNC Symbol;Acc:HGNC:71]	0.47	2.94	6.21	0.00	0.00	up
HIST1H3E	histone cluster 1 H3 family member e [Source:HGNC Symbol;Acc:HGNC:4769]	1.27	3.51	2.76	0.00	0.00	up
IFI35	interferon induced protein 35 [Source:HGNC Symbol;Acc:HGNC:5399]	3.01	7.43	2.46	0.00	0.00	up
ZFP90	ZFP90 zinc finger protein [Source:HGNC Symbol;Acc:HGNC:23329]	1.46	4.39	3.01	0.00	0.00	up
AC104452	novel protein	0.74	1.78	2.40	0.00	0.00	up
OTX1	orthodenticle homeobox 1 [Source:HGNC Symbol;Acc:HGNC:8521]	4.05	8.36	2.06	0.00	0.00	up
RERG	RAS like estrogen regulated growth inhibitor [Source:HGNC Symbol;Acc:HGNC:15980]	0.20	0.74	3.78	0.00	0.00	up
ZNF658B	zinc finger protein 658B (pseudogene) [Source:HGNC Symbol;Acc:HGNC:32033]	0.44	1.43	3.22	0.00	0.00	up
ZW10	zw10 kinetochore protein [Source:HGNC Symbol;Acc:HGNC:13194]	3.27	6.82	2.09	0.00	0.00	up
AC010834	novel transcript	0.77	3.83	4.97	0.00	0.00	up
ZNF252P	zinc finger protein 252, pseudogene [Source:HGNC Symbol;Acc:HGNC:13046]	1.11	2.25	2.03	0.00	0.00	up
AC023157	interferon induced transmembrane protein 3 (1-8U) (IFITM3) pseudogene	0.53	2.90	5.48	0.00	0.00	up
IDNK	IDNK, gluconokinase [Source:HGNC Symbol;Acc:HGNC:31367]	0.74	2.28	3.08	0.00	0.00	up
PRKAR1B	protein kinase cAMP-dependent type I regulatory subunit beta [Source:HGNC Symbol;Acc:HGNC:	0.80	2.02	2.54	0.00	0.00	up
BCL2L15	BCL2 like 15 [Source:HGNC Symbol;Acc:HGNC:33624]	0.39	1.11	2.83	0.00	0.00	up

AC016394	novel transcript, antisense to TTC18	1.42	4.53	3.20	0.00	0.00	up
CLEC18A	C-type lectin domain family 18 member A [Source:HGNC Symbol;Acc:HGNC:30388]	0.04	0.20	4.85	0.00	0.00	up
HOXC10	homeobox C10 [Source:HGNC Symbol;Acc:HGNC:5122]	1.44	3.50	2.43	0.00	0.00	up
ENO3	enolase 3 [Source:HGNC Symbol;Acc:HGNC:3354]	2.10	4.25	2.03	0.00	0.00	up
SEPT7-AS1	SEPT7 antisense RNA 1 (head to head) [Source:HGNC Symbol;Acc:HGNC:51153]	0.24	0.77	3.22	0.00	0.00	up
LINC00659	long intergenic non-protein coding RNA 659 [Source:HGNC Symbol;Acc:HGNC:44316]	13.88	29.79	2.15	0.00	0.00	up
C19orf38	chromosome 19 open reading frame 38 [Source:HGNC Symbol;Acc:HGNC:34073]	0.13	0.80	6.00	0.00	0.00	up
THAP2	THAP domain containing 2 [Source:HGNC Symbol;Acc:HGNC:20854]	0.48	1.29	2.67	0.00	0.00	up
AC024560	novel transcript	0.10	0.56	5.90	0.00	0.00	up
HFE	homeostatic iron regulator [Source:HGNC Symbol;Acc:HGNC:4886]	0.25	0.82	3.34	0.00	0.00	up
TNC	tenascin C [Source:HGNC Symbol;Acc:HGNC:5318]	0.57	1.33	2.35	0.00	0.00	up
AC016705	novel transcript	0.07	0.26	3.69	0.00	0.00	up
RAMACL	RNA guanine-7 methyltransferase activating subunit like (pseudogene) [Source:HGNC Symbol;Acc:	0.15	1.27	8.24	0.00	0.00	up
FAM86HP	family with sequence similarity 86 member H, pseudogene [Source:HGNC Symbol;Acc:HGNC:423	0.41	1.55	3.75	0.00	0.00	up
TMC1	transmembrane channel like 1 [Source:HGNC Symbol;Acc:HGNC:16513]	0.21	0.57	2.75	0.00	0.00	up
EGR3	early growth response 3 [Source:HGNC Symbol;Acc:HGNC:3240]	0.79	1.85	2.34	0.00	0.00	up
RGS19	regulator of G protein signaling 19 [Source:HGNC Symbol;Acc:HGNC:13735]	3.15	6.87	2.18	0.00	0.00	up
NEK11	NIMA related kinase 11 [Source:HGNC Symbol;Acc:HGNC:18593]	0.20	0.62	3.16	0.00	0.00	up
CLDN2	claudin 2 [Source:HGNC Symbol;Acc:HGNC:2041]	0.79	2.05	2.60	0.00	0.01	up
ZBTB45P2	zinc finger and BTB domain containing 45 pseudogene 2 [Source:HGNC Symbol;Acc:HGNC:4922	4.31	8.96	2.08	0.00	0.01	up
HRK	harakiri, BCL2 interacting protein [Source:HGNC Symbol;Acc:HGNC:5185]	0.80	1.87	2.36	0.00	0.01	up
CALML6	calmodulin like 6 [Source:HGNC Symbol;Acc:HGNC:24193]	0.74	2.05	2.77	0.00	0.01	up
DNAJC30	DnaJ heat shock protein family (Hsp40) member C30 [Source:HGNC Symbol;Acc:HGNC:16410]	1.30	3.17	2.43	0.00	0.01	up
FOXM1	forkhead box M1 [Source:HGNC Symbol;Acc:HGNC:3818]	3.06	6.39	2.09	0.00	0.01	up
AC084346	novel transcript	7.20	16.28	2.26	0.00	0.01	up
AL645608	novel transcript	0.21	0.70	3.33	0.00	0.01	up
COL20A1	collagen type XX alpha 1 chain [Source:HGNC Symbol;Acc:HGNC:14670]	0.02	0.08	4.75	0.00	0.01	up
PANX2	pannexin 2 [Source:HGNC Symbol;Acc:HGNC:8600]	1.77	4.19	2.36	0.00	0.01	up
TMEM44-AS1	TMEM44 antisense RNA 1 [Source:HGNC Symbol;Acc:HGNC:44272]	1.53	4.09	2.68	0.00	0.01	up
FER	FER tyrosine kinase [Source:HGNC Symbol;Acc:HGNC:3655]	0.62	2.14	3.45	0.00	0.01	up
NECAP1	NECAP endocytosis associated 1 [Source:HGNC Symbol;Acc:HGNC:24539]	1.49	3.36	2.25	0.00	0.01	up
PSMG3-AS1	PSMG3 antisense RNA 1 (head to head) [Source:HGNC Symbol;Acc:HGNC:22230]	0.24	0.65	2.75	0.00	0.01	up
STPG3-AS1	STPG3 antisense RNA 1 [Source:HGNC Symbol;Acc:HGNC:51176]	0.12	0.58	4.65	0.00	0.01	up
LINC01719	long intergenic non-protein coding RNA 1719 [Source:HGNC Symbol;Acc:HGNC:52506]	0.46	1.37	2.96	0.00	0.01	up
SDCBP2-AS1	SDCBP2 antisense RNA 1 [Source:HGNC Symbol;Acc:HGNC:44314]	0.50	1.35	2.71	0.00	0.01	up
HSPA1A	heat shock protein family A (Hsp70) member 1A [Source:HGNC Symbol;Acc:HGNC:5232]	0.78	15.19	19.48	0.00	0.01	up
WIPF2	WAS/WASL interacting protein family member 2 [Source:HGNC Symbol;Acc:HGNC:30923]	2.46	5.78	2.35	0.00	0.01	up
CLDN4	claudin 4 [Source:HGNC Symbol;Acc:HGNC:2046]	3.14	33.73	10.75	0.00	0.01	up
DDN	dendrin [Source:HGNC Symbol;Acc:HGNC:24458]	1.75	3.56	2.03	0.00	0.01	up
FOXA3	forkhead box A3 [Source:HGNC Symbol;Acc:HGNC:5023]	1.70	3.85	2.27	0.00	0.01	up
HEXIM2	HEXIM P-TEFb complex subunit 2 [Source:HGNC Symbol;Acc:HGNC:28591]	3.22	6.54	2.03	0.00	0.01	up
SUMO2P17	SUMO2 pseudogene 17 [Source:HGNC Symbol;Acc:HGNC:49358]	2.34	5.92	2.53	0.00	0.01	up
LINC01431	long intergenic non-protein coding RNA 1431 [Source:HGNC Symbol;Acc:HGNC:50744]	0.37	1.57	4.21	0.00	0.01	up
MSX1	msh homeobox 1 [Source:HGNC Symbol;Acc:HGNC:7391]	57.42	116.30	2.03	0.00	0.01	up
AC010547	novel transcript	0.32	1.89	5.83	0.00	0.01	up
ATF5	activating transcription factor 5 [Source:HGNC Symbol;Acc:HGNC:790]	0.94	2.49	2.66	0.00	0.01	up
NBR2	neighbor of BRCA1 lncRNA 2 [Source:HGNC Symbol;Acc:HGNC:20691]	0.96	2.43	2.53	0.00	0.01	up
NFIC	nuclear factor I C [Source:HGNC Symbol;Acc:HGNC:7786]	1.65	3.93	2.38	0.00	0.01	up
AC093884	novel protein, WDFY1-AP1S3 readthrough	0.01	0.43	56.98	0.00	0.01	up
LINC00909	long intergenic non-protein coding RNA 909 [Source:HGNC Symbol;Acc:HGNC:44331]	0.78	1.88	2.41	0.00	0.01	up
LINC00342	long intergenic non-protein coding RNA 342 [Source:HGNC Symbol;Acc:HGNC:42470]	2.14	4.59	2.14	0.00	0.01	up
AC026191	novel transcript, antisense to THUMPD3	0.15	0.75	4.99	0.00	0.01	up
C9orf43	chromosome 9 open reading frame 43 [Source:HGNC Symbol;Acc:HGNC:23570]	0.16	0.75	4.61	0.00	0.01	up
AC080112	novel transcript, antisense to GJD3	0.91	2.29	2.51	0.00	0.01	up
AC159540	novel transcript	2.29	5.39	2.35	0.00	0.01	up
AC244033	novel transcript	0.21	0.75	3.52	0.00	0.01	up
AC074143	novel transcript	0.19	1.20	6.42	0.00	0.01	up
LINC02361	long intergenic non-protein coding RNA 2361 [Source:HGNC Symbol;Acc:HGNC:53283]	1.76	4.20	2.39	0.00	0.01	up
CENPBD1	CENPB DNA-binding domain containing 1 [Source:HGNC Symbol;Acc:HGNC:28272]	0.62	1.61	2.60	0.00	0.01	up
ACTBP1	actin, beta pseudogene 1 [Source:HGNC Symbol;Acc:HGNC:134]	0.33	1.15	3.51	0.00	0.01	up
DDIT4L	DNA damage inducible transcript 4 like [Source:HGNC Symbol;Acc:HGNC:30555]	1.82	3.85	2.12	0.00	0.01	up
AC062037	novel transcript	0.17	0.58	3.43	0.00	0.01	up
HOXD3	homeobox D3 [Source:HGNC Symbol;Acc:HGNC:5137]	0.06	0.34	5.37	0.00	0.01	up
ISY1	ISY1 splicing factor homolog [Source:HGNC Symbol;Acc:HGNC:29201]	2.45	4.96	2.02	0.00	0.01	up
CRB1	crumbs cell polarity complex component 1 [Source:HGNC Symbol;Acc:HGNC:2343]	0.03	0.12	3.60	0.00	0.02	up
GABRG2	gamma-aminobutyric acid type A receptor gamma2 subunit [Source:HGNC Symbol;Acc:HGNC:40	0.06	0.15	2.73	0.00	0.02	up
PCBP1-AS1	PCBP1 antisense RNA 1 [Source:HGNC Symbol;Acc:HGNC:42948]	0.39	0.79	2.03	0.00	0.02	up
LPXN	leupaxin [Source:HGNC Symbol;Acc:HGNC:14061]	1.05	2.40	2.28	0.00	0.02	up
CDPF1	cysteine rich DPF motif domain containing 1 [Source:HGNC Symbol;Acc:HGNC:33710]	3.63	7.26	2.00	0.00	0.02	up
ABCC11	ATP binding cassette subfamily C member 11 [Source:HGNC Symbol;Acc:HGNC:14639]	0.03	0.16	5.16	0.00	0.02	up
TEX19	testis expressed 19 [Source:HGNC Symbol;Acc:HGNC:33802]	0.69	1.78	2.59	0.00	0.02	up
AL355581	novel transcript, antisense to L3MBTL3	0.06	0.26	4.26	0.00	0.02	up
AC104825	novel transcript	0.53	1.33	2.49	0.00	0.02	up
MOGS	mannosyl-oligosaccharide glucosidase [Source:HGNC Symbol;Acc:HGNC:24862]	8.11	16.44	2.03	0.00	0.02	up
XIAP	X-linked inhibitor of apoptosis [Source:HGNC Symbol;Acc:HGNC:592]	0.64	2.04	3.21	0.00	0.02	up
AC131160	presenilin associated rhomboid like [Source:NCBI gene;Acc:55486]	0.16	2.60	16.30	0.00	0.02	up
SP5	Sp5 transcription factor [Source:HGNC Symbol;Acc:HGNC:14529]	1.95	4.63	2.38	0.00	0.02	up
AL353708	novel transcript, antisense to TOR1AIP1	0.33	1.13	3.44	0.00	0.02	up
CPB2-AS1	CPB2 antisense RNA 1 [Source:HGNC Symbol;Acc:HGNC:39898]	0.10	0.30	3.15	0.00	0.02	up
IMPG2	interphotoreceptor matrix proteoglycan 2 [Source:HGNC Symbol;Acc:HGNC:18362]	0.12	0.32	2.72	0.00	0.02	up
AC006504	novel transcript	0.16	0.52	3.33	0.00	0.02	up
PDE7B	phosphodiesterase 7B [Source:HGNC Symbol;Acc:HGNC:8792]	0.11	0.33	3.00	0.00	0.02	up
AP001099	novel transcript	0.41	1.39	3.40	0.00	0.02	up
DLEC1	DLEC1, cilia and flagella associated protein [Source:HGNC Symbol;Acc:HGNC:2899]	0.05	0.15	3.36	0.00	0.02	up
ITIH4	inter-alpha-trypsin inhibitor heavy chain family member 4 [Source:HGNC Symbol;Acc:HGNC:6169	0.16	0.53	3.23	0.00	0.02	up
NICN1	nicolin 1 [Source:HGNC Symbol;Acc:HGNC:18317]	0.13	0.56	4.31	0.00	0.02	up
ZCWPW1	zinc finger CW-type and PWWP domain containing 1 [Source:HGNC Symbol;Acc:HGNC:23486]	0.16	0.52	3.17	0.00	0.02	up
AL117336	novel transcript, antisense to CCNY	0.45	1.33	2.99	0.00	0.02	up
ZNF503-AS1	ZNF503 antisense RNA 1 [Source:HGNC Symbol;Acc:HGNC:27370]	0.27	0.78	2.92	0.00	0.02	up
SAMD14	sterile alpha motif domain containing 14 [Source:HGNC Symbol;Acc:HGNC:27312]	0.16	0.43	2.60	0.00	0.02	up
CIITA	class II major histocompatibility complex transactivator [Source:HGNC Symbol;Acc:HGNC:7067]	0.06	0.18	3.15	0.00	0.02	up
AIFM3	apoptosis inducing factor, mitochondria associated 3 [Source:HGNC Symbol;Acc:HGNC:26398]	0.30	0.74	2.45	0.00	0.02	up
C13orf46	chromosome 13 open reading frame 46 [Source:HGNC Symbol;Acc:HGNC:53786]	0.08	0.29	3.79	0.00	0.03	up
AC008966	novel transcript, antisense to MOCS2	1.06	2.26	2.13	0.00	0.03	up
AC027307	novel transcript, antisense to APC2	3.00	6.56	2.19	0.00	0.03	up
AC138904	novel transcript LOC100506705	0.12	0.42	3.45	0.00	0.03	up
CCDC122	coiled-coil domain containing 122 [Source:HGNC Symbol;Acc:HGNC:26478]	0.38	0.89	2.34	0.00	0.03	up
AP002490	novel transcript	0.15	0.43	2.91	0.00	0.03	up
SMIM2-AS1	SMIM2 antisense RNA 1 [Source:HGNC Symbol;Acc:HGNC:42674]	0.07	0.44	6.43	0.00	0.03	up
FLRT3	fibronectin leucine rich transmembrane protein 3 [Source:HGNC Symbol;Acc:HGNC:3762]	0.06	0.20	3.41	0.00	0.03	up
CCDC17	coiled-coil domain containing 17 [Source:HGNC Symbol;Acc:HGNC:26574]	0.15	0.45	3.02	0.00	0.03	up
FOXH1	forkhead box H1 [Source:HGNC Symbol;Acc:HGNC:3814]	0.18	0.63	3.45	0.00	0.03	up
CD72	CD72 molecule [Source:HGNC Symbol;Acc:HGNC:1696]	0.96	2.17	2.25	0.00	0.03	up
AL355388	novel transcript, antisense to ARHGEF2	0.37	1.11	3.01	0.00	0.03	up
AC018809	novel transcript, antisense to TMEM111	0.16	0.45	2.92	0.00	0.03	up
AC012512	MAX-like protein X (MLX) pseudogene	1.61	4.39	2.72	0.00	0.03	up
TSSK3	testis specific serine kinase 3 [Source:HGNC Symbol;Acc:HGNC:15473]	0.08	0.57	6.81	0.00	0.03	up
C6orf163	chromosome 6 open reading frame 163 [Source:HGNC Symbol;Acc:HGNC:21403]	0.18	0.60	3.30	0.00	0.03	up
CCDC92	coiled-coil domain containing 92 [Source:HGNC Symbol;Acc:HGNC:29563]	0.47	1.00	2.12	0.00	0.03	up
LINC00622	long intergenic non-protein coding RNA 622 [Source:HGNC Symbol;Acc:HGNC:44251]	0.22	0.71	3.17	0.00	0.03	up
TRIM45	tripartite motif containing 45 [Source:HGNC Symbol;Acc:HGNC:19018]	0.87	1.80	2.07	0.00	0.03	up

LBX2-AS1	LBX2 antisense RNA 1 [Source:HGNC Symbol;Acc:HGNC:25136]	1.04	2.30	2.22	0.00	0.03	up
LDB3	LIM domain binding 3 [Source:HGNC Symbol;Acc:HGNC:15710]	0.04	0.13	3.55	0.00	0.03	up
AC005154	pseudogene similar golgi autoantigen, golgin subfamily a, 8A GOLGA8A	0.19	0.67	3.60	0.00	0.03	up
ZNF585B	zinc finger protein 585B [Source:HGNC Symbol;Acc:HGNC:30948]	0.62	1.26	2.04	0.00	0.03	up
AC007114	novel transcript	3.08	6.65	2.16	0.00	0.03	up
AC009802	novel transcript, antisense to SERPINB8	0.22	0.74	3.33	0.00	0.03	up
TUBB6	tubulin beta 6 class V [Source:HGNC Symbol;Acc:HGNC:20776]	4.69	10.40	2.22	0.00	0.03	up
PNPLA7	patatin like phospholipase domain containing 7 [Source:HGNC Symbol;Acc:HGNC:24768]	0.11	0.32	2.98	0.00	0.03	up
SNRPGP2	small nuclear ribonucleoprotein polypeptide G pseudogene 2 [Source:HGNC Symbol;Acc:HGNC:3(	2.48	9.03	3.65	0.00	0.03	up
WDR78	WD repeat domain 78 [Source:HGNC Symbol;Acc:HGNC:26252]	0.12	0.35	2.86	0.00	0.03	up
AC108174	novel transcript	1.92	4.56	2.37	0.00	0.03	up
PPEF1	protein phosphatase with EF-hand domain 1 [Source:HGNC Symbol;Acc:HGNC:9243]	0.09	0.31	3.39	0.00	0.03	up
AC004890	zinc finger protein pseudogene	0.45	1.22	2.70	0.00	0.03	up
AC106017	novel transcript	0.15	0.55	3.71	0.00	0.03	up
TMPO-AS1	TMPO antisense RNA 1 [Source:HGNC Symbol;Acc:HGNC:44158]	0.29	0.74	2.58	0.00	0.03	up
MUC20	mucin 20, cell surface associated [Source:HGNC Symbol;Acc:HGNC:23282]	0.24	0.58	2.45	0.00	0.04	up
MMP7	matrix metallopeptidase 7 [Source:HGNC Symbol;Acc:HGNC:7174]	1.74	3.50	2.01	0.00	0.04	up
AL513165	novel transcript	0.24	1.08	4.49	0.00	0.04	up
ZNF594	zinc finger protein 594 [Source:HGNC Symbol;Acc:HGNC:29392]	0.36	0.79	2.22	0.00	0.04	up
SRRM2-AS1	SRRM2 antisense RNA 1 [Source:HGNC Symbol;Acc:HGNC:44162]	0.18	0.47	2.63	0.00	0.04	up
PRR13P5	proline rich 13 pseudogene 5 [Source:HGNC Symbol;Acc:HGNC:50618]	5.52	13.39	2.42	0.00	0.04	up
RIBC1	RIB43A domain with coiled-coils 1 [Source:HGNC Symbol;Acc:HGNC:26537]	0.13	0.44	3.34	0.00	0.04	up
AC121761	novel transcript, antisense to GLIPR1	2.12	4.31	2.03	0.00	0.04	up
MTTP	microsomal triglyceride transfer protein [Source:HGNC Symbol;Acc:HGNC:7467]	0.09	0.26	2.99	0.00	0.04	up
ELAC1	elaC ribonuclease Z 1 [Source:HGNC Symbol;Acc:HGNC:14197]	0.69	1.46	2.12	0.00	0.04	up
LINC01910	long intergenic non-protein coding RNA 1910 [Source:HGNC Symbol;Acc:HGNC:52729]	0.39	0.99	2.54	0.00	0.04	up
AC105233	novel transcript	0.43	0.88	2.06	0.00	0.04	up
AC010969	novel transcript	0.75	1.59	2.14	0.00	0.04	up
DEGS2	delta 4-desaturase, sphingolipid 2 [Source:HGNC Symbol;Acc:HGNC:20113]	0.25	0.70	2.85	0.00	0.04	up
BTC	betacellulin [Source:HGNC Symbol;Acc:HGNC:1121]	0.46	1.07	2.34	0.00	0.04	up
ELF5	E74 like ETS transcription factor 5 [Source:HGNC Symbol;Acc:HGNC:3320]	0.40	0.93	2.30	0.00	0.04	up
LURAP1L	leucine rich adaptor protein 1 like [Source:HGNC Symbol;Acc:HGNC:31452]	0.95	1.96	2.07	0.00	0.05	up
PPIL6	peptidylprolyl isomerase like 6 [Source:HGNC Symbol;Acc:HGNC:21557]	0.26	0.64	2.47	0.00	0.05	up
AC079781	novel transcript	1.05	4.54	4.33	0.00	0.05	up
AL596244	novel transcript, overlapping TTLL11	0.71	1.46	2.05	0.00	0.05	up
AC092683	novel transcript	0.98	3.41	3.48	0.00	0.05	up
WAS	Wiskott-Aldrich syndrome [Source:HGNC Symbol;Acc:HGNC:12731]	0.63	1.45	2.29	0.00	0.05	up
ZNF41	zinc finger protein 41 [Source:HGNC Symbol;Acc:HGNC:13107]	0.79	1.86	2.36	0.00	0.05	up
KCNK3	potassium two pore domain channel subfamily K member 3 [Source:HGNC Symbol;Acc:HGNC:62'	0.17	0.45	2.57	0.00	0.05	up
AC025048	novel transcript	0.75	1.81	2.39	0.00	0.05	up
AC112484	novel transcript	0.75	2.19	2.92	0.00	0.05	up
AL390039	general transcription factor IIIC, polypeptide 6, alpha 35kDa (GTF3C6) pseudogene	0.69	2.04	2.95	0.00	0.05	up
CDK3	cyclin dependent kinase 3 [Source:HGNC Symbol;Acc:HGNC:1772]	0.64	1.90	2.96	0.00	0.05	up
HOXC6	homeobox C6 [Source:HGNC Symbol;Acc:HGNC:5128]	0.43	1.03	2.43	0.00	0.06	up
LINC00205	long intergenic non-protein coding RNA 205 [Source:HGNC Symbol;Acc:HGNC:16420]	1.57	5.79	3.70	0.00	0.06	up
CLHC1	clathrin heavy chain linker domain containing 1 [Source:HGNC Symbol;Acc:HGNC:26453]	0.50	1.00	2.01	0.00	0.06	up
AKAP3	A-kinase anchoring protein 3 [Source:HGNC Symbol;Acc:HGNC:373]	0.74	1.49	2.00	0.00	0.06	up
TEX9	testis expressed 9 [Source:HGNC Symbol;Acc:HGNC:29585]	0.29	0.66	2.25	0.00	0.06	up
ETFRF1	electron transfer flavoprotein regulatory factor 1 [Source:HGNC Symbol;Acc:HGNC:27052]	1.42	3.11	2.19	0.00	0.06	up
FAM227B	family with sequence similarity 227 member B [Source:HGNC Symbol;Acc:HGNC:26543]	0.43	0.87	2.05	0.00	0.06	up
RNF4	ring finger protein 4 [Source:HGNC Symbol;Acc:HGNC:10067]	1.97	4.77	2.42	0.00	0.06	up
GDPGP1	GDP-D-glucose phosphorylase 1 [Source:HGNC Symbol;Acc:HGNC:34360]	0.21	0.53	2.51	0.00	0.06	up
AC110285	novel transcript	1.49	3.19	2.15	0.00	0.06	up
AC004593	chimerin 2 [Source:NCBI gene;Acc:1124]	0.03	0.23	9.00	0.00	0.06	up
COX20P1	COX20, cytochrome c oxidase assembly factor pseudogene 1 [Source:HGNC Symbol;Acc:HGNC:3	1.65	4.37	2.66	0.00	0.06	up
NAALADL2	N-acetylated alpha-linked acidic dipeptidase like 2 [Source:HGNC Symbol;Acc:HGNC:23219]	0.13	0.28	2.24	0.00	0.06	up
AC022239	novel transcript, antisense to BLK	0.61	1.41	2.31	0.00	0.06	up
TRIM74	tripartite motif containing 74 [Source:HGNC Symbol;Acc:HGNC:17453]	0.79	1.86	2.36	0.00	0.06	up
KRT13	keratin 13 [Source:HGNC Symbol;Acc:HGNC:6415]	0.68	1.45	2.12	0.00	0.07	up
AC090061	novel transcript	0.20	0.58	2.90	0.01	0.07	up
HSFX3	heat shock transcription factor family, X-linked member 3 [Source:HGNC Symbol;Acc:HGNC:5239	0.54	1.37	2.53	0.01	0.07	up
AC025569	novel transcript, antisense to PTPRB	0.70	1.80	2.56	0.01	0.07	up
AC140479	novel transcript	2.34	10.99	4.69	0.01	0.07	up
AC012513	TEC	0.64	1.53	2.38	0.01	0.07	up
NEB	nebulin [Source:HGNC Symbol;Acc:HGNC:7720]	0.03	0.08	2.53	0.01	0.07	up
ENTPD3-AS1	ENTPD3 antisense RNA 1 [Source:HGNC Symbol;Acc:HGNC:26710]	0.25	0.64	2.52	0.01	0.07	up
INTS6-AS1	INTS6 antisense RNA 1 [Source:HGNC Symbol;Acc:HGNC:42691]	0.25	0.50	2.02	0.01	0.07	up
FAM228B	family with sequence similarity 228 member B [Source:HGNC Symbol;Acc:HGNC:24736]	0.45	1.08	2.38	0.01	0.07	up
ASB16-AS1	ASB16 antisense RNA 1 [Source:HGNC Symbol;Acc:HGNC:25442]	1.69	3.68	2.18	0.01	0.07	up
AL162586	novel transcript	0.38	0.91	2.39	0.01	0.07	up
MTERF1	mitochondrial transcription termination factor 1 [Source:HGNC Symbol;Acc:HGNC:21463]	0.60	1.91	3.20	0.01	0.07	up
PRR22	proline rich 22 [Source:HGNC Symbol;Acc:HGNC:28354]	0.32	0.86	2.67	0.01	0.07	up
FAM166B	family with sequence similarity 166 member B [Source:HGNC Symbol;Acc:HGNC:34242]	0.44	1.16	2.67	0.01	0.07	up
CYP2E1	cytochrome P450 family 2 subfamily E member 1 [Source:HGNC Symbol;Acc:HGNC:2631]	0.09	0.22	2.46	0.01	0.07	up
AC139100	novel transcript	1.32	3.28	2.48	0.01	0.07	up
WEE2-AS1	WEE2 antisense RNA 1 [Source:HGNC Symbol;Acc:HGNC:48669]	0.11	0.31	2.91	0.01	0.07	up
ZNF572	zinc finger protein 572 [Source:HGNC Symbol;Acc:HGNC:26758]	0.36	0.85	2.37	0.01	0.07	up
AC004069	novel transcript	1.74	4.41	2.54	0.01	0.07	up
UHMK1	U2AF homology motif kinase 1 [Source:HGNC Symbol;Acc:HGNC:19683]	6.14	12.85	2.09	0.01	0.07	up
PIH1D2	PIH1 domain containing 2 [Source:HGNC Symbol;Acc:HGNC:25210]	0.12	0.35	2.82	0.01	0.08	up
AC010327	novel transcript, antisense to C19orf51 and SYT5	0.25	0.72	2.90	0.01	0.08	up
AC005534	novel transcript	0.64	1.73	2.70	0.01	0.08	up
NPIPB15	nuclear pore complex interacting protein family member B15 [Source:HGNC Symbol;Acc:HGNC:3	0.97	2.07	2.13	0.01	0.08	up
AP002851	novel transcript	0.34	0.89	2.60	0.01	0.08	up
TPH1	tryptophan hydroxylase 1 [Source:HGNC Symbol;Acc:HGNC:12008]	0.05	0.16	3.12	0.01	0.08	up
CACTIN-AS1	CACTIN antisense RNA 1 [Source:HGNC Symbol;Acc:HGNC:31391]	0.21	0.59	2.78	0.01	0.08	up
AL024497	novel transcript	1.49	3.93	2.64	0.01	0.08	up
CLIP3	CAP-Gly domain containing linker protein 3 [Source:HGNC Symbol;Acc:HGNC:24314]	0.53	1.10	2.08	0.01	0.08	up
AC004585	novel transcript	1.19	2.67	2.24	0.01	0.08	up
AL022322	novel transcript, antisense to BAIAP2L2	0.70	1.66	2.39	0.01	0.08	up
AC006254	novel MUSTN1-ITIH4 readthrough	0.33	0.70	2.14	0.01	0.08	up
AGBL3	ATP/GTP binding protein like 3 [Source:HGNC Symbol;Acc:HGNC:27981]	0.14	0.33	2.38	0.01	0.08	up
SPATA18	spermatogenesis associated 18 [Source:HGNC Symbol;Acc:HGNC:29579]	0.14	0.34	2.40	0.01	0.09	up
PRTN3	proteinase 3 [Source:HGNC Symbol;Acc:HGNC:9495]	1.55	3.25	2.09	0.01	0.09	up
NPIPB14P	nuclear pore complex interacting protein family member B14, pseudogene [Source:HGNC Symbol;A	0.74	2.09	2.81	0.01	0.09	up
DNAJC3-DT	DNAJC3 divergent transcript [Source:HGNC Symbol;Acc:HGNC:39808]	0.48	1.02	2.10	0.01	0.09	up
FAIM	Fas apoptotic inhibitory molecule [Source:HGNC Symbol;Acc:HGNC:18703]	0.45	0.91	2.04	0.01	0.09	up
DNM1P35	dynamin 1 pseudogene 35 [Source:HGNC Symbol;Acc:HGNC:35182]	0.14	0.41	2.91	0.01	0.09	up
AC010997	novel transcript	0.20	0.53	2.60	0.01	0.09	up
AL645933	novel transcript	0.36	1.00	2.79	0.01	0.09	up
AL035661	novel transcript	1.14	2.56	2.25	0.01	0.09	up
RAB11B-AS1	RAB11B antisense RNA 1 [Source:HGNC Symbol;Acc:HGNC:44178]	0.46	1.16	2.52	0.01	0.09	up
CPLX2	complexin 2 [Source:HGNC Symbol;Acc:HGNC:2310]	0.17	0.38	2.25	0.01	0.09	up
C17orf100	chromosome 17 open reading frame 100 [Source:HGNC Symbol;Acc:HGNC:34494]	0.25	0.59	2.37	0.01	0.10	up
RAP2C-AS1	RAP2C antisense RNA 1 [Source:HGNC Symbol;Acc:HGNC:40957]	0.24	0.55	2.36	0.01	0.10	up
FAM27E3	family with sequence similarity 27 member E3 [Source:HGNC Symbol;Acc:HGNC:28655]	0.61	1.52	2.51	0.01	0.10	up
AC097478	novel transcript, antisense to SNCA	0.60	1.80	3.01	0.01	0.10	up
TCAM1P	testicular cell adhesion molecule 1, pseudogene [Source:HGNC Symbol;Acc:HGNC:30707]	0.32	0.72	2.25	0.01	0.10	up
TRIM73	tripartite motif containing 73 [Source:HGNC Symbol;Acc:HGNC:18162]	0.37	0.77	2.10	0.01	0.10	up
BISPR	BST2 interferon stimulated positive regulator [Source:HGNC Symbol;Acc:HGNC:51290]	0.21	0.54	2.59	0.01	0.10	up
SH2D6	SH2 domain containing 6 [Source:HGNC Symbol;Acc:HGNC:30439]	0.26	0.58	2.20	0.01	0.10	up

TLK2P1	tousled like kinase 2 pseudogene 1 [Source:HGNC Symbol;Acc:HGNC:18048]	0.54	1.16	2.15	0.01	0.10	up
LINC00482	long intergenic non-protein coding RNA 482 [Source:HGNC Symbol;Acc:HGNC:26816]	0.83	1.75	2.10	0.01	0.11	up
AC104823	novel transcript	0.53	1.29	2.44	0.01	0.11	up
RBM26-AS1	RBM26 antisense RNA 1 [Source:HGNC Symbol;Acc:HGNC:39805]	0.34	0.71	2.10	0.01	0.11	up
MIR210HG	MIR210 host gene [Source:HGNC Symbol;Acc:HGNC:39524]	1.13	2.44	2.15	0.01	0.11	up
ZSWIM5	zinc finger SWIM-type containing 5 [Source:HGNC Symbol;Acc:HGNC:29299]	0.25	0.52	2.08	0.01	0.11	up
LINC02454	long intergenic non-protein coding RNA 2454 [Source:HGNC Symbol;Acc:HGNC:53387]	2.00	4.16	2.09	0.01	0.11	up
AP4S1	adaptor related protein complex 4 subunit sigma 1 [Source:HGNC Symbol;Acc:HGNC:575]	0.20	0.40	2.05	0.01	0.11	up
LINC02577	long intergenic non-protein coding RNA 2577 [Source:HGNC Symbol;Acc:HGNC:53749]	0.28	0.60	2.11	0.01	0.11	up
LCN12	lipocalin 12 [Source:HGNC Symbol;Acc:HGNC:28733]	0.15	0.40	2.67	0.01	0.11	up
ZNF747	zinc finger protein 747 [Source:HGNC Symbol;Acc:HGNC:28350]	0.33	0.75	2.25	0.01	0.11	up
AP002380	novel transcript	0.14	0.45	3.14	0.01	0.11	up
BX322234	novel transcript	0.47	1.20	2.53	0.01	0.11	up
AC007405	glutamate rich 2 [Source:NCBI gene;Acc:285141]	1.63	3.49	2.13	0.01	0.11	up
AL390879	transmembrane 9 superfamily member 2 (TM9SF2) pseudogene	0.18	0.45	2.47	0.01	0.12	up
AC068338	novel transcript, antisense to NEIL1	0.85	2.41	2.83	0.01	0.12	up
MCPH1-AS1	MCPH1 antisense RNA 1 [Source:HGNC Symbol;Acc:HGNC:51655]	0.27	0.64	2.42	0.01	0.12	up
GLIPR1L2	GLIPR1 like 2 [Source:HGNC Symbol;Acc:HGNC:28592]	0.17	0.42	2.50	0.01	0.12	up
LINC02593	long intergenic non-protein coding RNA 2593 [Source:HGNC Symbol;Acc:HGNC:53933]	0.34	0.71	2.06	0.01	0.12	up
Z97180	novel pseudogene	0.55	1.41	2.56	0.01	0.12	up
AL590652	novel transcript	0.31	0.73	2.34	0.01	0.12	up
SPX	spexin hormone [Source:HGNC Symbol;Acc:HGNC:28139]	0.02	0.12	5.15	0.01	0.12	up
AK9	adenylate kinase 9 [Source:HGNC Symbol;Acc:HGNC:33814]	0.06	0.15	2.46	0.01	0.12	up
AL109811	novel transcript, antisense to MASP2	1.04	2.30	2.20	0.01	0.12	up
ST20	suppressor of tumorigenicity 20 [Source:HGNC Symbol;Acc:HGNC:33520]	0.83	1.96	2.35	0.01	0.12	up
AC027117	novel transcript	0.16	0.44	2.71	0.01	0.12	up
CTBP1-DT	CTBP1 divergent transcript [Source:HGNC Symbol;Acc:HGNC:28307]	0.19	0.53	2.78	0.01	0.12	up
PPP1R26-AS1	PPP1R26 antisense RNA 1 [Source:HGNC Symbol;Acc:HGNC:48717]	0.18	0.44	2.43	0.01	0.12	up
TMEM164	transmembrane protein 164 [Source:HGNC Symbol;Acc:HGNC:26217]	2.26	4.67	2.07	0.01	0.12	up
THAP7-AS1	THAP7 antisense RNA 1 [Source:HGNC Symbol;Acc:HGNC:41013]	0.49	0.99	2.05	0.01	0.12	up
EXOC3-AS1	EXOC3 antisense RNA 1 [Source:HGNC Symbol;Acc:HGNC:25175]	0.69	1.48	2.15	0.01	0.12	up
TREX1	three prime repair exonuclease 1 [Source:HGNC Symbol;Acc:HGNC:12269]	0.75	2.88	3.86	0.01	0.13	up
AL135910	novel transcript	0.32	0.66	2.09	0.01	0.13	up
DIAPH2-AS1	DIAPH2 antisense RNA 1 [Source:HGNC Symbol;Acc:HGNC:16972]	0.14	0.53	3.91	0.01	0.13	up
TEN1	TEN1, CST complex subunit [Source:HGNC Symbol;Acc:HGNC:37242]	0.49	2.24	4.59	0.02	0.13	up
POC1B-AS1	POC1B antisense RNA 1 [Source:HGNC Symbol;Acc:HGNC:52949]	0.10	0.23	2.27	0.02	0.13	up
LEPROT	leptin receptor overlapping transcript [Source:HGNC Symbol;Acc:HGNC:29477]	1.71	3.44	2.01	0.02	0.13	up
C2orf15	chromosome 2 open reading frame 15 [Source:HGNC Symbol;Acc:HGNC:28436]	0.51	1.55	3.05	0.02	0.13	up
AL513497	TEC	0.38	0.84	2.23	0.02	0.13	up
CRYZL2P	crystallin zeta like 2, pseudogene [Source:HGNC Symbol;Acc:HGNC:52164]	0.58	1.27	2.20	0.02	0.13	up
ZNF180	zinc finger protein 180 [Source:HGNC Symbol;Acc:HGNC:12970]	0.73	1.62	2.23	0.02	0.13	up
AC023157	novel transcript, antisense to AMN1	0.28	0.70	2.47	0.02	0.13	up
DNAJC28	DnaJ heat shock protein family (Hsp40) member C28 [Source:HGNC Symbol;Acc:HGNC:1297]	0.47	0.99	2.08	0.02	0.14	up
AL359921	novel transcript	0.86	2.04	2.36	0.02	0.14	up
LINC01869	long intergenic non-protein coding RNA 1869 [Source:HGNC Symbol;Acc:HGNC:25050]	0.30	0.83	2.78	0.02	0.14	up
CNTD1	cyclin N-terminal domain containing 1 [Source:HGNC Symbol;Acc:HGNC:26847]	0.26	0.62	2.41	0.02	0.14	up
AC090114	novel transcript	0.08	0.20	2.53	0.02	0.14	up
KCNH8	potassium voltage-gated channel subfamily H member 8 [Source:HGNC Symbol;Acc:HGNC:18864	0.18	0.39	2.18	0.02	0.14	up
TNFSF4	TNF superfamily member 4 [Source:HGNC Symbol;Acc:HGNC:11934]	0.13	0.30	2.39	0.02	0.14	up
AC012184	novel transcript, antisense to DDX19B and DDX19A	0.05	0.41	8.23	0.02	0.14	up
EIF2S3B	eukaryotic translation initiation factor 2 subunit gamma B [Source:HGNC Symbol;Acc:HGNC:4386	0.51	1.55	3.05	0.02	0.14	up
AC021054	novel transcript	0.17	0.38	2.28	0.02	0.14	up
FOXD4	forkhead box D4 [Source:HGNC Symbol;Acc:HGNC:3805]	0.56	1.25	2.25	0.02	0.14	up
FAM226B	family with sequence similarity 226 member B [Source:HGNC Symbol;Acc:HGNC:31964]	0.67	1.40	2.10	0.02	0.14	up
CTAGE6	CTAGE family member 6 [Source:HGNC Symbol;Acc:HGNC:28644]	0.28	1.02	3.63	0.02	0.14	up
AC012313	novel transcript	0.14	0.32	2.32	0.02	0.14	up
SNRPGP10	small nuclear ribonucleoprotein polypeptide G pseudogene 10 [Source:HGNC Symbol;Acc:HGNC:3	14.19	28.69	2.02	0.02	0.15	up
PCOLCE-AS1	PCOLCE antisense RNA 1 [Source:HGNC Symbol;Acc:HGNC:40430]	0.12	0.33	2.70	0.02	0.15	up
LINC02043	long intergenic non-protein coding RNA 2043 [Source:HGNC Symbol;Acc:HGNC:52883]	0.31	0.88	2.81	0.02	0.15	up
GEMIN8P4	gem nuclear organelle associated protein 8 pseudogene 4 [Source:HGNC Symbol;Acc:HGNC:3797	0.52	1.31	2.53	0.02	0.15	up
APOM	apolipoprotein M [Source:HGNC Symbol;Acc:HGNC:13916]	0.64	1.42	2.20	0.02	0.15	up
PTGES3L-AARS	PTGES3L-AARSD1 readthrough [Source:HGNC Symbol;Acc:HGNC:43946]	1.62	6.83	4.23	0.02	0.15	up
AC020891	novel transcript	0.41	1.04	2.53	0.02	0.15	up
NUTM2D	NUT family member 2D [Source:HGNC Symbol;Acc:HGNC:23447]	0.12	0.27	2.18	0.02	0.15	up
TMCC1-AS1	TMCC1 antisense RNA 1 (head to head) [Source:HGNC Symbol;Acc:HGNC:49060]	0.31	0.63	2.01	0.02	0.15	up
AC073585	CUB and zona pellucida-like domain-containing protein 1 [Source:UniProtKB/Swiss-Prot;Acc:Q86	0.16	0.39	2.42	0.02	0.15	up
AC079174	novel transcript, antisense to CKAP4	0.24	0.59	2.44	0.02	0.15	up
TSN	translin [Source:HGNC Symbol;Acc:HGNC:12379]	7.37	14.93	2.03	0.02	0.15	up
GLIDR	glioblastoma down-regulated RNA [Source:HGNC Symbol;Acc:HGNC:48823]	0.12	0.29	2.46	0.02	0.16	up
AC105052	novel transcript, sense overlapping POLR2J2, UPK3BL and RASA4	0.32	0.75	2.32	0.02	0.16	up
AC092123	novel transcript, antisense to SPG7	0.48	1.10	2.29	0.02	0.16	up
IL20RB	interleukin 20 receptor subunit beta [Source:HGNC Symbol;Acc:HGNC:6004]	0.29	0.63	2.16	0.02	0.16	up
ACRBP	acrosin binding protein [Source:HGNC Symbol;Acc:HGNC:17195]	0.13	0.37	2.82	0.02	0.17	up
AL137779	novel transcript, antisense to PPP2R5C	0.99	2.46	2.48	0.02	0.17	up
CAPN3	calpain 3 [Source:HGNC Symbol;Acc:HGNC:1480]	0.17	0.56	3.24	0.02	0.17	up
ZNF233	zinc finger protein 233 [Source:HGNC Symbol;Acc:HGNC:30946]	0.13	0.30	2.37	0.02	0.17	up
PYGO1	pygopus family PHD finger 1 [Source:HGNC Symbol;Acc:HGNC:30256]	0.07	0.16	2.30	0.02	0.17	up
ABO	ABO, alpha 1-3-N-acetylgalactosaminyltransferase and alpha 1-3-galactosyltransferase [Source:HGN	0.07	0.17	2.46	0.02	0.17	up
ZNF429	zinc finger protein 429 [Source:HGNC Symbol;Acc:HGNC:20817]	0.09	0.19	2.13	0.02	0.17	up
AP000356	breakpoint cluster region pseudogene 3	0.21	0.55	2.66	0.02	0.17	up
IGFL4	IGF like family member 4 [Source:HGNC Symbol;Acc:HGNC:32931]	0.18	0.43	2.33	0.02	0.17	up
GATM	glycine amidinotransferase [Source:HGNC Symbol;Acc:HGNC:4175]	0.11	0.25	2.24	0.02	0.17	up
ITGB2-AS1	ITGB2 antisense RNA 1 [Source:HGNC Symbol;Acc:HGNC:44304]	0.15	0.35	2.40	0.02	0.17	up
LPP-AS2	LPP antisense RNA 2 [Source:HGNC Symbol;Acc:HGNC:27952]	0.10	0.27	2.57	0.02	0.17	up
AL592435	novel transcript	0.13	0.31	2.41	0.02	0.17	up
AC005041	novel transcript, antisense to LBX2	0.99	2.38	2.40	0.02	0.18	up
PDCL3P4	phosducin-like 3 pseudogene 4 [Source:HGNC Symbol;Acc:HGNC:44505]	0.42	1.13	2.73	0.02	0.18	up
TF	transferrin [Source:HGNC Symbol;Acc:HGNC:11740]	0.14	0.28	2.00	0.03	0.18	up
TBX2	T-box 2 [Source:HGNC Symbol;Acc:HGNC:11597]	0.10	0.24	2.46	0.03	0.18	up
KDR	kinase insert domain receptor [Source:HGNC Symbol;Acc:HGNC:6307]	0.11	0.24	2.15	0.03	0.18	up
TSPAN10	tetraspanin 10 [Source:HGNC Symbol;Acc:HGNC:29942]	0.17	0.38	2.24	0.03	0.18	up
LINC00565	long intergenic non-protein coding RNA 565 [Source:HGNC Symbol;Acc:HGNC:43709]	0.13	0.40	3.08	0.03	0.18	up
UFSP1	UFM1 specific peptidase 1 (inactive) [Source:HGNC Symbol;Acc:HGNC:33821]	0.74	1.61	2.17	0.03	0.18	up
N4BP2L1	NEDD4 binding protein 2 like 1 [Source:HGNC Symbol;Acc:HGNC:25037]	0.12	0.26	2.08	0.03	0.18	up
CYB561D1	cytochrome b561 family member D1 [Source:HGNC Symbol;Acc:HGNC:26804]	0.46	1.33	2.86	0.03	0.18	up
RRN3P2	RRN3 homolog, RNA polymerase I transcription factor pseudogene 2 [Source:HGNC Symbol;Acc:I	0.24	0.54	2.30	0.03	0.18	up
AC073655	novel transcript, antisense to PLXNC1	0.30	1.00	3.37	0.03	0.18	up
SLC22A20P	solute carrier family 22 member 20, pseudogene [Source:HGNC Symbol;Acc:HGNC:29867]	0.08	0.24	2.89	0.03	0.18	up
SLC7A2	solute carrier family 7 member 2 [Source:HGNC Symbol;Acc:HGNC:11060]	0.68	1.51	2.21	0.03	0.19	up
ZNF585A	zinc finger protein 585A [Source:HGNC Symbol;Acc:HGNC:26305]	0.11	0.27	2.46	0.03	0.19	up
EXTL3-AS1	EXTL3 antisense RNA 1 [Source:HGNC Symbol;Acc:HGNC:27985]	0.13	0.28	2.12	0.03	0.19	up
AC020910	novel transcript	0.29	0.76	2.65	0.03	0.19	up
AP003498	novel transcript	0.39	0.89	2.30	0.03	0.19	up
EML2-AS1	EML2 antisense RNA 1 [Source:HGNC Symbol;Acc:HGNC:48331]	0.42	0.97	2.30	0.03	0.19	up
IL10RB-DT	IL10RB divergent transcript [Source:HGNC Symbol;Acc:HGNC:44303]	0.22	1.35	6.02	0.03	0.19	up
RN7SK	RNA, 7SK small nuclear [Source:HGNC Symbol;Acc:HGNC:10037]	8.57	17.96	2.10	0.03	0.19	up
ASPRV1	aspartic peptidase retroviral like 1 [Source:HGNC Symbol;Acc:HGNC:26321]	0.25	0.68	2.76	0.03	0.19	up
GCNA	germ cell nuclear acidic peptidase [Source:HGNC Symbol;Acc:HGNC:15805]	0.19	0.42	2.22	0.03	0.19	up
BCDIN3D-AS1	BCDIN3D antisense RNA 1 [Source:HGNC Symbol;Acc:HGNC:44113]	0.22	0.49	2.19	0.03	0.19	up
AL627171	novel transcript	0.27	0.64	2.42	0.03	0.19	up
NKPD1	NTPase KAP family P-loop domain containing 1 [Source:HGNC Symbol;Acc:HGNC:24739]	0.14	0.38	2.63	0.03	0.19	up

FTH1P3	ferritin heavy chain 1 pseudogene 3 [Source:HGNC Symbol;Acc:HGNC:3990]	2.01	4.28	2.13	0.03	0.19	up
SAXO2	stabilizer of axonemal microtubules 2 [Source:HGNC Symbol;Acc:HGNC:33727]	0.25	0.50	2.01	0.03	0.20	up
ZNF493	zinc finger protein 493 [Source:HGNC Symbol;Acc:HGNC:23708]	0.08	0.18	2.19	0.03	0.20	up
STRC	stereocilin [Source:HGNC Symbol;Acc:HGNC:16035]	0.11	0.24	2.20	0.03	0.20	up
AL645929	MHC class I polypeptide-related sequence F pseudogene	0.21	0.44	2.08	0.03	0.20	up
LRRC32	leucine rich repeat containing 32 [Source:HGNC Symbol;Acc:HGNC:4161]	0.14	0.34	2.53	0.03	0.20	up
DNAJC27-AS1	DNAJC27 antisense RNA 1 [Source:HGNC Symbol;Acc:HGNC:42943]	0.19	0.47	2.51	0.03	0.20	up
CLK2P1	CDC like kinase 2, pseudogene 1 [Source:HGNC Symbol;Acc:HGNC:2070]	0.20	0.69	3.39	0.03	0.20	up
KLF8	Kruppel like factor 8 [Source:HGNC Symbol;Acc:HGNC:6351]	0.03	0.08	2.25	0.03	0.20	up
AC007906	novel protein	0.39	0.81	2.06	0.03	0.20	up
AC239799	novel transcript	0.16	0.43	2.64	0.03	0.20	up
ZNF268	zinc finger protein 268 [Source:HGNC Symbol;Acc:HGNC:13061]	0.09	0.21	2.42	0.03	0.21	up
PHKA2-AS1	PHKA2 antisense RNA 1 [Source:HGNC Symbol;Acc:HGNC:44110]	0.95	1.92	2.03	0.03	0.21	up
CCDC33	coiled-coil domain containing 33 [Source:HGNC Symbol;Acc:HGNC:26552]	0.11	0.25	2.30	0.03	0.21	up
AL928654	novel transcript, antisense to MTA1	0.31	0.70	2.25	0.03	0.21	up
SHLD1	shieldin complex subunit 1 [Source:HGNC Symbol;Acc:HGNC:26318]	0.37	0.75	2.06	0.03	0.21	up
AC092171	uncharacterized LOC100129484 [Source:NCBI gene;Acc:100129484]	0.12	0.34	2.82	0.03	0.21	up
TAS2R14	taste 2 receptor member 14 [Source:HGNC Symbol;Acc:HGNC:14920]	0.24	0.57	2.41	0.03	0.21	up
DRC7	dynein regulatory complex subunit 7 [Source:HGNC Symbol;Acc:HGNC:25289]	0.12	0.26	2.11	0.03	0.21	up
AC008105	novel transcript, antisense to FMNL1	0.53	1.11	2.10	0.03	0.21	up
C16orf71	chromosome 16 open reading frame 71 [Source:HGNC Symbol;Acc:HGNC:25081]	0.10	0.22	2.24	0.03	0.21	up
AC121761	novel transcript, sense intronic to CAPS2	0.61	1.44	2.36	0.03	0.21	up
AL662899	novel protein	0.90	8.44	9.40	0.04	0.22	up
AL391152	novel transcript	0.29	0.69	2.36	0.04	0.22	up
AC132872	novel transcript, antisense CCDC57	0.31	0.66	2.15	0.04	0.22	up
AC136475	novel transcript, antisense to IFITM3	0.31	0.69	2.21	0.04	0.22	up
YJEFN3	YjeF N-terminal domain containing 3 [Source:HGNC Symbol;Acc:HGNC:24785]	1.03	3.71	3.58	0.04	0.22	up
AC091173	novel transcript	0.27	0.55	2.00	0.04	0.22	up
MCF2L-AS1	MCF2L antisense RNA 1 [Source:HGNC Symbol;Acc:HGNC:39825]	0.89	1.80	2.02	0.04	0.22	up
AC022007	novel transcript	0.45	0.91	2.05	0.04	0.22	up
STARD7-AS1	STARD7 antisense RNA 1 [Source:HGNC Symbol;Acc:HGNC:40827]	0.44	0.90	2.07	0.04	0.22	up
MSH4	mutS homolog 4 [Source:HGNC Symbol;Acc:HGNC:7327]	0.18	0.39	2.25	0.04	0.22	up
AP001972	TEC	3.04	9.50	3.13	0.04	0.23	up
AC005726	novel transcript, sense intronic to novel protein	0.95	2.18	2.30	0.04	0.23	up
AC008555	novel transcript	0.85	1.81	2.13	0.04	0.23	up
SGMS1-AS1	SGMS1 antisense RNA 1 [Source:HGNC Symbol;Acc:HGNC:49683]	0.06	0.12	2.04	0.04	0.23	up
DPEP1	dipeptidase 1 [Source:HGNC Symbol;Acc:HGNC:3002]	0.24	0.50	2.12	0.04	0.23	up
SLC22A4	solute carrier family 22 member 4 [Source:HGNC Symbol;Acc:HGNC:10968]	0.18	0.42	2.32	0.04	0.23	up
AGO1	argonaute RISC catalytic component 1 [Source:HGNC Symbol;Acc:HGNC:3262]	1.24	2.50	2.02	0.04	0.24	up
AC104794	novel transcript	0.36	0.73	2.04	0.04	0.24	up
ZNF596	zinc finger protein 596 [Source:HGNC Symbol;Acc:HGNC:27268]	0.20	0.43	2.09	0.04	0.24	up
VPREB3	V-set pre-B cell surrogate light chain 3 [Source:HGNC Symbol;Acc:HGNC:12710]	1.02	2.37	2.31	0.04	0.24	up
ZNF814	zinc finger protein 814 [Source:HGNC Symbol;Acc:HGNC:33258]	0.22	0.45	2.03	0.04	0.24	up
ZNF396	zinc finger protein 396 [Source:HGNC Symbol;Acc:HGNC:18824]	0.14	0.30	2.14	0.04	0.24	up
Z82186	novel transcript	0.12	0.24	2.05	0.04	0.24	up
PPP3CB-AS1	PPP3CB antisense RNA 1 (head to head) [Source:HGNC Symbol;Acc:HGNC:50750]	0.14	0.31	2.14	0.05	0.25	up
AC022400	novel transcript	0.21	0.78	3.72	0.05	0.25	up
AL132639	novel transcript, antisense to CTAGE5	0.38	0.81	2.15	0.05	0.25	up
AC010491	novel transcript, antisense to FAM105B	0.21	0.49	2.27	0.05	0.25	up
GGACT	gamma-glutamylamine cyclotransferase [Source:HGNC Symbol;Acc:HGNC:25100]	0.44	0.99	2.27	0.05	0.25	up
LINC01301	long intergenic non-protein coding RNA 1301 [Source:HGNC Symbol;Acc:HGNC:50464]	0.18	0.39	2.14	0.05	0.25	up
ZHX1-C8orf76	ZHX1-C8orf76 readthrough [Source:HGNC Symbol;Acc:HGNC:42975]	1.18	2.98	2.53	0.05	0.25	up
AC021739	novel transcript, antisense to AKAP13	0.42	1.13	2.69	0.05	0.25	up
HIC1	HIC ZBTB transcriptional repressor 1 [Source:HGNC Symbol;Acc:HGNC:4909]	0.39	0.99	2.56	0.05	0.25	up
DAG1	dystroglycan 1 [Source:HGNC Symbol;Acc:HGNC:2666]	17.47	2.30	0.13	0.00	0.00	down
AC117378	novel protein	11.45	0.00	0.00	0.00	0.00	down
NPHP3-ACAD11	NPHP3-ACAD11 readthrough (NMD candidate) [Source:HGNC Symbol;Acc:HGNC:48351]	0.47	0.00	0.00	0.00	0.00	down
SUSD2	sushi domain containing 2 [Source:HGNC Symbol;Acc:HGNC:30667]	3.08	0.46	0.15	0.00	0.00	down
NPIPB5	nuclear pore complex interacting protein family member B5 [Source:HGNC Symbol;Acc:HGNC:37	8.09	2.78	0.34	0.00	0.00	down
TENT5B	terminal nucleotidyltransferase 5B [Source:HGNC Symbol;Acc:HGNC:28273]	16.89	5.25	0.31	0.00	0.00	down
RASAL2	RAS protein activator like 2 [Source:HGNC Symbol;Acc:HGNC:9874]	1.27	0.28	0.22	0.00	0.00	down
GOLGA6L9	golgin A6 family-like 9 [Source:HGNC Symbol;Acc:HGNC:37229]	3.89	0.97	0.25	0.00	0.00	down
PGRMC1	progesterone receptor membrane component 1 [Source:HGNC Symbol;Acc:HGNC:16090]	53.17	22.09	0.42	0.00	0.00	down
SORL1	sortilin related receptor 1 [Source:HGNC Symbol;Acc:HGNC:11185]	5.74	2.35	0.41	0.00	0.00	down
KRTAP2-3	keratin associated protein 2-3 [Source:HGNC Symbol;Acc:HGNC:18906]	91.75	36.74	0.40	0.00	0.00	down
CCN1	cellular communication network factor 1 [Source:HGNC Symbol;Acc:HGNC:2654]	49.56	18.23	0.37	0.00	0.00	down
CA2	carbonic anhydrase 2 [Source:HGNC Symbol;Acc:HGNC:1373]	19.41	7.25	0.37	0.00	0.00	down
INSIG1	insulin induced gene 1 [Source:HGNC Symbol;Acc:HGNC:6083]	23.84	5.96	0.25	0.00	0.00	down
TSPAN13	tetraspanin 13 [Source:HGNC Symbol;Acc:HGNC:21643]	37.05	15.75	0.43	0.00	0.00	down
PGM2L1	phosphoglucomutase 2 like 1 [Source:HGNC Symbol;Acc:HGNC:20898]	2.31	0.68	0.30	0.00	0.00	down
MINDY2	MINDY lysine 48 deubiquitinase 2 [Source:HGNC Symbol;Acc:HGNC:26954]	4.49	0.63	0.14	0.00	0.00	down
MSMO1	methylsterol monooxygenase 1 [Source:HGNC Symbol;Acc:HGNC:10545]	43.75	19.36	0.44	0.00	0.00	down
SLC4A8	solute carrier family 4 member 8 [Source:HGNC Symbol;Acc:HGNC:11034]	0.87	0.26	0.30	0.00	0.00	down
VSIR	V-set immunoregulatory receptor [Source:HGNC Symbol;Acc:HGNC:30085]	10.85	4.93	0.45	0.00	0.00	down
RTEL1-TNFRSF(	RTEL1-TNFRSF6B readthrough (NMD candidate) [Source:HGNC Symbol;Acc:HGNC:44095]	28.98	12.36	0.43	0.00	0.00	down
CPA4	carboxypeptidase A4 [Source:HGNC Symbol;Acc:HGNC:15740]	15.87	7.36	0.46	0.00	0.00	down
HMGB1	high mobility group box 1 [Source:HGNC Symbol;Acc:HGNC:4983]	34.60	15.95	0.46	0.00	0.00	down
SCD	stearoyl-CoA desaturase [Source:HGNC Symbol;Acc:HGNC:10571]	122.78	61.31	0.50	0.00	0.00	down
ADGRB2	adhesion G protein-coupled receptor B2 [Source:HGNC Symbol;Acc:HGNC:944]	4.80	1.99	0.41	0.00	0.00	down
GCOM1	GRINL1A complex locus 1 [Source:HGNC Symbol;Acc:HGNC:26424]	0.70	0.16	0.22	0.00	0.00	down
LINC00641	long intergenic non-protein coding RNA 641 [Source:HGNC Symbol;Acc:HGNC:27511]	9.39	4.40	0.47	0.00	0.00	down
STARD4	StAR related lipid transfer domain containing 4 [Source:HGNC Symbol;Acc:HGNC:18058]	13.06	5.99	0.46	0.00	0.00	down
AL035461	novel protein	1.79	0.00	0.00	0.00	0.00	down
TIGD1	tigger transposable element derived 1 [Source:HGNC Symbol;Acc:HGNC:14523]	2.91	0.00	0.00	0.00	0.00	down
APC	APC, WNT signaling pathway regulator [Source:HGNC Symbol;Acc:HGNC:583]	1.58	0.34	0.21	0.00	0.00	down
IDI1	isopentenyl-diphosphate delta isomerase 1 [Source:HGNC Symbol;Acc:HGNC:5387]	11.27	5.24	0.46	0.00	0.00	down
MYC	MYC proto-oncogene, bHLH transcription factor [Source:HGNC Symbol;Acc:HGNC:7553]	32.71	2.39	0.07	0.00	0.00	down
AC009812	novel transcript	0.37	0.03	0.08	0.00	0.00	down
AL133230	novel transcript, antisense to PTPN1	0.36	0.04	0.12	0.00	0.00	down
IL1RAP	interleukin 1 receptor accessory protein [Source:HGNC Symbol;Acc:HGNC:5995]	1.93	0.82	0.42	0.00	0.00	down
SGPP1	sphingosine-1-phosphate phosphatase 1 [Source:HGNC Symbol;Acc:HGNC:17720]	9.44	4.37	0.46	0.00	0.00	down
CD79B	CD79b molecule [Source:HGNC Symbol;Acc:HGNC:1699]	0.89	0.12	0.14	0.00	0.00	down
GJB3	gap junction protein beta 3 [Source:HGNC Symbol;Acc:HGNC:4285]	16.02	7.99	0.50	0.00	0.00	down
DEK	DEK proto-oncogene [Source:HGNC Symbol;Acc:HGNC:2768]	11.74	5.71	0.49	0.00	0.00	down
UBE2S	ubiquitin conjugating enzyme E2 S [Source:HGNC Symbol;Acc:HGNC:17895]	124.78	57.49	0.46	0.00	0.00	down
AC015712	novel transcript, antisense to LRRK1 and ALDH1A3	6.51	2.91	0.45	0.00	0.00	down
PADI3	peptidyl arginine deiminase 3 [Source:HGNC Symbol;Acc:HGNC:18337]	2.92	0.97	0.33	0.00	0.00	down
AC092073	novel protein, readthrough between GPI and PDCD2	1.74	0.00	0.00	0.00	0.00	down
CEND1	cell cycle exit and neuronal differentiation 1 [Source:HGNC Symbol;Acc:HGNC:24153]	1.17	0.13	0.11	0.00	0.00	down
POLR2J2	RNA polymerase II subunit J2 [Source:HGNC Symbol;Acc:HGNC:23208]	1.18	0.12	0.10	0.00	0.00	down
SAR1A	secretion associated Ras related GTPase 1A [Source:HGNC Symbol;Acc:HGNC:10534]	11.87	4.46	0.38	0.00	0.00	down
NUAK1	NUAK family kinase 1 [Source:HGNC Symbol;Acc:HGNC:14311]	4.41	2.19	0.50	0.00	0.00	down
RC3H1	ring finger and CCCH-type domains 1 [Source:HGNC Symbol;Acc:HGNC:29434]	1.89	0.33	0.18	0.00	0.00	down
MRPL10	mitochondrial ribosomal protein L10 [Source:HGNC Symbol;Acc:HGNC:14055]	38.18	16.18	0.42	0.00	0.00	down
HIST2H2BE	histone cluster 2 H2B family member e [Source:HGNC Symbol;Acc:HGNC:4760]	9.66	3.72	0.38	0.00	0.00	down
NDE1	nudE neurodevelopment protein 1 [Source:HGNC Symbol;Acc:HGNC:17619]	9.89	4.11	0.42	0.00	0.00	down
CCN2	cellular communication network factor 2 [Source:HGNC Symbol;Acc:HGNC:2500]	25.93	10.11	0.39	0.00	0.00	down
PIM2	Pim-2 proto-oncogene, serine/threonine kinase [Source:HGNC Symbol;Acc:HGNC:8987]	8.27	3.99	0.48	0.00	0.00	down
SSC4D	scavenger receptor cysteine rich family member with 4 domains [Source:HGNC Symbol;Acc:HGNC	1.39	0.38	0.27	0.00	0.00	down
PRR5-ARHGAP8	PRR5-ARHGAP8 readthrough [Source:HGNC Symbol;Acc:HGNC:34512]	3.40	0.42	0.12	0.00	0.00	down
DES	desmin [Source:HGNC Symbol;Acc:HGNC:2770]	1.33	0.28	0.21	0.00	0.00	down

FAM72C	family with sequence similarity 72 member C [Source:HGNC Symbol;Acc:HGNC:30602]	9.08	4.38	0.48	0.00	0.00	down
ACBD7	acyl-CoA binding domain containing 7 [Source:HGNC Symbol;Acc:HGNC:17715]	3.04	1.20	0.40	0.00	0.00	down
SNORD26	small nucleolar RNA, C/D box 26 [Source:HGNC Symbol;Acc:HGNC:10148]	63.51	19.10	0.30	0.00	0.00	down
IQGAP2	IQ motif containing GTPase activating protein 2 [Source:HGNC Symbol;Acc:HGNC:6111]	1.24	0.48	0.38	0.00	0.00	down
ADAM32	ADAM metallopeptidase domain 32 [Source:HGNC Symbol;Acc:HGNC:15479]	0.44	0.13	0.31	0.00	0.00	down
CALHM3	calcium homeostasis modulator 3 [Source:HGNC Symbol;Acc:HGNC:23458]	1.54	0.36	0.24	0.00	0.00	down
AL031714	novel transcript, antisense to UBE2I	2.04	0.67	0.33	0.00	0.00	down
NANOS1	nanos C2HC-type zinc finger 1 [Source:HGNC Symbol;Acc:HGNC:23044]	1.03	0.31	0.30	0.00	0.00	down
FRMD5	FERM domain containing 5 [Source:HGNC Symbol;Acc:HGNC:28214]	11.84	5.92	0.50	0.00	0.00	down
TMSB15A	thymosin beta 15a [Source:HGNC Symbol;Acc:HGNC:30744]	15.60	6.22	0.40	0.00	0.00	down
AL591806	novel transcript, TSTD1 - F11R readthrough	6.75	0.41	0.06	0.00	0.00	down
AC015712	novel transcript, antisense to ALDH1A3	5.30	1.13	0.21	0.00	0.00	down
LGR4	leucine rich repeat containing G protein-coupled receptor 4 [Source:HGNC Symbol;Acc:HGNC:132	2.65	1.26	0.47	0.00	0.00	down
SNORA8	small nucleolar RNA, H/ACA box 8 [Source:HGNC Symbol;Acc:HGNC:32596]	67.63	27.29	0.40	0.00	0.00	down
FAM92B	family with sequence similarity 92 member B [Source:HGNC Symbol;Acc:HGNC:24781]	1.20	0.31	0.26	0.00	0.00	down
MAP3K14	mitogen-activated protein kinase kinase kinase 14 [Source:HGNC Symbol;Acc:HGNC:6853]	6.14	3.00	0.49	0.00	0.00	down
JAZF1	JAZF zinc finger 1 [Source:HGNC Symbol;Acc:HGNC:28917]	3.46	1.63	0.47	0.00	0.00	down
DEPP1	DEPP1, autophagy regulator [Source:HGNC Symbol;Acc:HGNC:23355]	3.77	1.64	0.43	0.00	0.00	down
EHBP1L1	EH domain binding protein 1 like 1 [Source:HGNC Symbol;Acc:HGNC:30682]	18.56	9.21	0.50	0.00	0.00	down
CPXM1	carboxypeptidase X, M14 family member 1 [Source:HGNC Symbol;Acc:HGNC:15771]	0.61	0.06	0.10	0.00	0.00	down
MED12L	mediator complex subunit 12 like [Source:HGNC Symbol;Acc:HGNC:16050]	1.32	0.63	0.48	0.00	0.00	down
AC015813	novel transcript	5.90	2.30	0.39	0.00	0.00	down
ITGAX	integrin subunit alpha X [Source:HGNC Symbol;Acc:HGNC:6152]	0.70	0.24	0.34	0.00	0.00	down
FKTN	fukutin [Source:HGNC Symbol;Acc:HGNC:3622]	1.30	0.36	0.28	0.00	0.00	down
GNL3LP1	G protein nucleolar 3 like pseudogene 1 [Source:HGNC Symbol;Acc:HGNC:25733]	1.01	0.24	0.24	0.00	0.00	down
ADGRV1	adhesion G protein-coupled receptor V1 [Source:HGNC Symbol;Acc:HGNC:17416]	0.20	0.07	0.38	0.00	0.00	down
AC105429	novel transcript, sense intronic to ZCCHC14	0.71	0.20	0.28	0.00	0.00	down
AL049844	novel transcript	2.40	0.42	0.17	0.00	0.00	down
AF129075	novel transcript, antisense to CCT8	7.39	2.85	0.39	0.00	0.00	down
NOP14-AS1	NOP14 antisense RNA 1 [Source:HGNC Symbol;Acc:HGNC:20205]	1.98	0.78	0.40	0.00	0.00	down
SDHAP2	succinate dehydrogenase complex flavoprotein subunit A pseudogene 2 [Source:HGNC Symbol;Acc	2.23	0.10	0.04	0.00	0.00	down
SNORA18	small nucleolar RNA, H/ACA box 18 [Source:HGNC Symbol;Acc:HGNC:32608]	67.32	29.99	0.45	0.00	0.00	down
FRMD6	FERM domain containing 6 [Source:HGNC Symbol;Acc:HGNC:19839]	1.77	0.80	0.45	0.00	0.00	down
FXYD6	FXYD domain containing ion transport regulator 6 [Source:HGNC Symbol;Acc:HGNC:4030]	1.55	0.67	0.43	0.00	0.00	down
AC011498	novel transcript	1.04	0.30	0.28	0.00	0.00	down
FAM217B	family with sequence similarity 217 member B [Source:HGNC Symbol;Acc:HGNC:16170]	0.97	0.26	0.27	0.00	0.00	down
HIST2H2AA3	histone cluster 2 H2A family member a3 [Source:HGNC Symbol;Acc:HGNC:4736]	30.01	10.81	0.36	0.00	0.00	down
AC021087	novel protein	1.65	0.30	0.18	0.00	0.00	down
AP000692	novel transcript, sense overlapping MORC3	1.44	0.25	0.17	0.00	0.00	down
AC027290	novel transcript	0.64	0.21	0.33	0.00	0.00	down
UMAD1	UBAP1-MVB12-associated (UMA) domain containing 1 [Source:HGNC Symbol;Acc:HGNC:4895	4.08	1.39	0.34	0.00	0.00	down
AP003392	novel transcript, antisense to CCDC84	2.43	0.53	0.22	0.00	0.00	down
AL359851	novel transcript	0.17	0.03	0.21	0.00	0.00	down
SESN3	sestrin 3 [Source:HGNC Symbol;Acc:HGNC:23060]	0.95	0.44	0.46	0.00	0.01	down
AC106886	novel transcript, antisense to RNF40	1.51	0.51	0.34	0.00	0.01	down
AL359922	novel transcript, antisense MTAP	2.40	0.87	0.36	0.00	0.01	down
DRP2	dystrophin related protein 2 [Source:HGNC Symbol;Acc:HGNC:3032]	0.24	0.06	0.27	0.00	0.01	down
PAG1	phosphoprotein membrane anchor with glycosphingolipid microdomains 1 [Source:HGNC Symbol;A	0.96	0.44	0.46	0.00	0.01	down
PLEKHG1	pleckstrin homology and RhoGEF domain containing G1 [Source:HGNC Symbol;Acc:HGNC:20884	1.01	0.43	0.43	0.00	0.01	down
FRY	FRY microtubule binding protein [Source:HGNC Symbol;Acc:HGNC:20367]	0.34	0.13	0.40	0.00	0.01	down
AC008147	novel transcript, sense intronic to LIMA1	0.64	0.21	0.33	0.00	0.01	down
TERT	telomerase reverse transcriptase [Source:HGNC Symbol;Acc:HGNC:11730]	0.95	0.37	0.39	0.00	0.01	down
RN7SKP23	RNA, 7SK small nuclear pseudogene 23 [Source:HGNC Symbol;Acc:HGNC:45747]	0.37	0.09	0.25	0.00	0.01	down
GRIK4	glutamate ionotropic receptor kainate type subunit 4 [Source:HGNC Symbol;Acc:HGNC:4582]	0.18	0.05	0.28	0.00	0.01	down
CHST1	carbohydrate sulfotransferase 1 [Source:HGNC Symbol;Acc:HGNC:1969]	0.95	0.36	0.38	0.00	0.01	down
SDCBP2	syndecan binding protein 2 [Source:HGNC Symbol;Acc:HGNC:15756]	1.00	0.32	0.32	0.00	0.01	down
DCLK1	doublecortin like kinase 1 [Source:HGNC Symbol;Acc:HGNC:2700]	0.60	0.28	0.47	0.00	0.01	down
MSRB3	methionine sulfoxide reductase B3 [Source:HGNC Symbol;Acc:HGNC:27375]	2.14	0.58	0.27	0.00	0.01	down
STBD1	starch binding domain 1 [Source:HGNC Symbol;Acc:HGNC:24854]	4.72	1.53	0.32	0.00	0.01	down
AC012181	novel transcript, sense intronic to HERPUD1	5.36	1.18	0.22	0.00	0.01	down
RPLP1P11	ribosomal protein lateral stalk subunit P1 pseudogene 11 [Source:HGNC Symbol;Acc:HGNC:36196	3.81	0.41	0.11	0.00	0.01	down
AC245452	novel transcript, antisense to PPM1F and TOP3B	0.20	0.09	0.46	0.00	0.01	down
PDXP	pyridoxal phosphatase [Source:HGNC Symbol;Acc:HGNC:30259]	8.90	1.96	0.22	0.00	0.01	down
NRXN2	neurexin 2 [Source:HGNC Symbol;Acc:HGNC:8009]	0.14	0.04	0.31	0.00	0.01	down
TRIM52	tripartite motif containing 52 [Source:HGNC Symbol;Acc:HGNC:19024]	2.11	0.95	0.45	0.00	0.01	down
ARHGAP11B	Rho GTPase activating protein 11B [Source:HGNC Symbol;Acc:HGNC:15782]	1.23	0.17	0.14	0.00	0.01	down
AC109587	novel transcript	0.61	0.21	0.35	0.00	0.01	down
CYP4F2	cytochrome P450 family 4 subfamily F member 2 [Source:HGNC Symbol;Acc:HGNC:2645]	0.42	0.11	0.25	0.00	0.01	down
SYT11	synaptotagmin 11 [Source:HGNC Symbol;Acc:HGNC:19239]	0.70	0.26	0.37	0.00	0.01	down
DNPH1	2'-deoxynucleoside 5'-phosphate N-hydrolase 1 [Source:HGNC Symbol;Acc:HGNC:21218]	36.15	18.03	0.50	0.00	0.01	down
RPSAP52	ribosomal protein SA pseudogene 52 [Source:HGNC Symbol;Acc:HGNC:35752]	3.19	1.34	0.42	0.00	0.02	down
DCAF17	DDB1 and CUL4 associated factor 17 [Source:HGNC Symbol;Acc:HGNC:25784]	1.10	0.45	0.41	0.00	0.02	down
FAM20A	FAM20A, golgi associated secretory pathway pseudokinase [Source:HGNC Symbol;Acc:HGNC:23	0.34	0.08	0.23	0.00	0.02	down
TNNC1	troponin C1, slow skeletal and cardiac type [Source:HGNC Symbol;Acc:HGNC:11943]	3.74	1.51	0.40	0.00	0.02	down
AC039056	novel transcript, sense intronic to EHD4	2.88	0.85	0.30	0.00	0.02	down
RF00099		36.64	15.30	0.42	0.00	0.02	down
AC009812	novel transcript	1.56	0.60	0.38	0.00	0.02	down
PFN1P6	profilin 1 pseudogene 6 [Source:HGNC Symbol;Acc:HGNC:42988]	2.72	0.75	0.28	0.00	0.02	down
PRDX3P1	peroxiredoxin 3 pseudogene 1 [Source:HGNC Symbol;Acc:HGNC:39264]	1.31	0.33	0.25	0.00	0.02	down
RUSC1-AS1	RUSC1 antisense RNA 1 [Source:HGNC Symbol;Acc:HGNC:26680]	1.70	0.76	0.45	0.00	0.02	down
KRTAP2-4	keratin associated protein 2-4 [Source:HGNC Symbol;Acc:HGNC:18891]	10.06	4.78	0.47	0.00	0.02	down
AL691442	novel ephrin-A4 (EFNA4) and ephrin-A3 (EFNA3) protein	2.40	0.69	0.29	0.00	0.02	down
TRAPPC5	trafficking protein particle complex 5 [Source:HGNC Symbol;Acc:HGNC:23067]	2.79	0.96	0.34	0.00	0.02	down
POU2F1	POU class 2 homeobox 1 [Source:HGNC Symbol;Acc:HGNC:9212]	3.26	1.36	0.42	0.00	0.03	down
CDC42SE2	CDC42 small effector 2 [Source:HGNC Symbol;Acc:HGNC:18547]	5.72	2.66	0.46	0.00	0.03	down
HAUS2	HAUS augmin like complex subunit 2 [Source:HGNC Symbol;Acc:HGNC:25530]	2.98	1.45	0.49	0.00	0.03	down
EBPL	EBP like [Source:HGNC Symbol;Acc:HGNC:18061]	20.54	9.12	0.44	0.00	0.03	down
SNORA4	small nucleolar RNA, H/ACA box 4 [Source:HGNC Symbol;Acc:HGNC:32587]	30.51	13.55	0.44	0.00	0.03	down
CDK19	cyclin dependent kinase 19 [Source:HGNC Symbol;Acc:HGNC:19338]	3.27	1.56	0.48	0.00	0.03	down
LINC02211	long intergenic non-protein coding RNA 2211 [Source:HGNC Symbol;Acc:HGNC:53078]	0.67	0.25	0.37	0.00	0.03	down
EBI3	Epstein-Barr virus induced 3 [Source:HGNC Symbol;Acc:HGNC:3129]	2.17	0.76	0.35	0.00	0.03	down
BDKRB2	bradykinin receptor B2 [Source:HGNC Symbol;Acc:HGNC:1030]	0.57	0.21	0.37	0.00	0.03	down
SLC9A9	solute carrier family 9 member A9 [Source:HGNC Symbol;Acc:HGNC:20653]	0.48	0.18	0.38	0.00	0.03	down
DNAH5	dynein axonemal heavy chain 5 [Source:HGNC Symbol;Acc:HGNC:2950]	0.22	0.10	0.44	0.00	0.03	down
PCDH19	protocadherin 19 [Source:HGNC Symbol;Acc:HGNC:14270]	0.17	0.06	0.33	0.00	0.03	down
AC132938	TEC	3.09	1.26	0.41	0.00	0.03	down
PHEX	phosphate regulating endopeptidase homolog X-linked [Source:HGNC Symbol;Acc:HGNC:8918]	0.22	0.07	0.33	0.00	0.04	down
GPRIN3	GPRIN family member 3 [Source:HGNC Symbol;Acc:HGNC:27733]	0.15	0.05	0.32	0.00	0.04	down
LINC00887	long intergenic non-protein coding RNA 887 [Source:HGNC Symbol;Acc:HGNC:48574]	0.49	0.15	0.30	0.00	0.04	down
WASH5P	WAS protein family homolog 5, pseudogene [Source:HGNC Symbol;Acc:HGNC:33884]	3.92	1.86	0.48	0.00	0.04	down
LINC00668	long intergenic non-protein coding RNA 668 [Source:HGNC Symbol;Acc:HGNC:44328]	0.25	0.08	0.33	0.00	0.04	down
AL136115	novel transcript	1.29	0.35	0.27	0.00	0.04	down
MEIOC	meiosis specific with coiled-coil domain [Source:HGNC Symbol;Acc:HGNC:26670]	0.25	0.09	0.36	0.00	0.04	down
HBA1	hemoglobin subunit alpha 1 [Source:HGNC Symbol;Acc:HGNC:4823]	4.36	1.96	0.45	0.00	0.04	down
AC122688	novel transcript	0.78	0.32	0.41	0.00	0.04	down
CEACAM1	carcinoembryonic antigen related cell adhesion molecule 1 [Source:HGNC Symbol;Acc:HGNC:181	0.90	0.43	0.47	0.00	0.04	down
ZNF24	zinc finger protein 24 [Source:HGNC Symbol;Acc:HGNC:13032]	11.96	4.93	0.41	0.00	0.04	down
SERPINA5	serpin family A member 5 [Source:HGNC Symbol;Acc:HGNC:8723]	0.42	0.09	0.23	0.00	0.04	down
C1QL1	complement C1q like 1 [Source:HGNC Symbol;Acc:HGNC:24182]	4.15	1.97	0.47	0.00	0.04	down
AC004057	ribosomal protein S26 (RPS26) pseudogene	64.38	28.37	0.44	0.00	0.05	down
KLF15	Kruppel like factor 15 [Source:HGNC Symbol;Acc:HGNC:14536]	2.15	1.05	0.49	0.00	0.05	down

NRIP2	nuclear receptor interacting protein 2 [Source:HGNC Symbol;Acc:HGNC:23078]	0.43	0.14	0.32	0.00	0.05	down
KCNN3	potassium calcium-activated channel subfamily N member 3 [Source:HGNC Symbol;Acc:HGNC:62	0.39	0.19	0.49	0.00	0.05	down
KRTAP3-1	keratin associated protein 3-1 [Source:HGNC Symbol;Acc:HGNC:16778]	1.52	0.42	0.28	0.00	0.05	down
TRPV6	transient receptor potential cation channel subfamily V member 6 [Source:HGNC Symbol;Acc:HGN	0.11	0.04	0.32	0.00	0.05	down
C3orf35	chromosome 3 open reading frame 35 [Source:HGNC Symbol;Acc:HGNC:24082]	1.31	0.61	0.47	0.00	0.05	down
ST6GALNAC3	ST6 N-acetylgalactosaminide alpha-2,6-sialyltransferase 3 [Source:HGNC Symbol;Acc:HGNC:193	0.92	0.45	0.49	0.00	0.05	down
AC023818	novel transcript	0.46	0.12	0.27	0.00	0.06	down
AL158163	novel transcript, antisense to BBIP1	0.71	0.22	0.31	0.00	0.06	down
AL049552	novel transcript	1.55	0.43	0.28	0.00	0.06	down
AC109460	novel transcript	1.40	0.55	0.39	0.00	0.06	down
LGALS7B	galectin 7B [Source:HGNC Symbol;Acc:HGNC:34447]	3.79	1.54	0.41	0.00	0.06	down
AJ239328	PCBP3 overlapping transcript 1	0.19	0.07	0.36	0.00	0.06	down
SRGAP1	SLIT-ROBO Rho GTPase activating protein 1 [Source:HGNC Symbol;Acc:HGNC:17382]	0.39	0.17	0.43	0.00	0.06	down
AC131212	novel transcript	0.75	0.25	0.34	0.00	0.06	down
PADI1	peptidyl arginine deiminase 1 [Source:HGNC Symbol;Acc:HGNC:18367]	0.35	0.14	0.40	0.00	0.06	down
ELMOD1	ELMO domain containing 1 [Source:HGNC Symbol;Acc:HGNC:25334]	0.95	0.46	0.48	0.00	0.06	down
VWF	von Willebrand factor [Source:HGNC Symbol;Acc:HGNC:12726]	0.12	0.04	0.33	0.00	0.06	down
AP001107	novel transcript	0.52	0.20	0.39	0.00	0.06	down
HIST2H2BF	histone cluster 2 H2B family member f [Source:HGNC Symbol;Acc:HGNC:24700]	0.83	0.33	0.40	0.01	0.07	down
AUXG01000058	COPG2 imprinted transcript 1	0.70	0.32	0.46	0.01	0.07	down
GCNT1	glucosaminyl (N-acetyl) transferase 1 [Source:HGNC Symbol;Acc:HGNC:4203]	0.47	0.15	0.32	0.01	0.07	down
HLA-DRB1	major histocompatibility complex, class II, DR beta 1 [Source:HGNC Symbol;Acc:HGNC:4948]	2.46	1.10	0.45	0.01	0.07	down
FAM189A2	family with sequence similarity 189 member A2 [Source:HGNC Symbol;Acc:HGNC:24820]	0.21	0.08	0.37	0.01	0.07	down
FRZB	frizzled related protein [Source:HGNC Symbol;Acc:HGNC:3959]	1.27	0.62	0.48	0.01	0.07	down
GRIN2B	glutamate ionotropic receptor NMDA type subunit 2B [Source:HGNC Symbol;Acc:HGNC:4586]	0.11	0.05	0.44	0.01	0.07	down
PRKG1	protein kinase cGMP-dependent 1 [Source:HGNC Symbol;Acc:HGNC:9414]	0.26	0.12	0.48	0.01	0.07	down
ANKRD26	ankyrin repeat domain 26 [Source:HGNC Symbol;Acc:HGNC:29186]	0.73	0.34	0.47	0.01	0.07	down
HIPK2	homeodomain interacting protein kinase 2 [Source:HGNC Symbol;Acc:HGNC:14402]	5.41	2.36	0.44	0.01	0.07	down
AL356019	novel transcript	0.80	0.39	0.48	0.01	0.08	down
SNORD27	small nucleolar RNA, C/D box 27 [Source:HGNC Symbol;Acc:HGNC:10149]	39.57	8.56	0.22	0.01	0.08	down
SCAMP5	secretory carrier membrane protein 5 [Source:HGNC Symbol;Acc:HGNC:30386]	1.67	0.60	0.36	0.01	0.08	down
CELF2-AS1	CELF2 antisense RNA 1 [Source:HGNC Symbol;Acc:HGNC:23515]	0.42	0.13	0.32	0.01	0.08	down
AC037459	novel protein	0.81	0.08	0.10	0.01	0.08	down
AC112128	novel protein	0.59	0.20	0.33	0.01	0.08	down
APLN	apelin [Source:HGNC Symbol;Acc:HGNC:16665]	0.77	0.33	0.42	0.01	0.08	down
MITF	melanocyte inducing transcription factor [Source:HGNC Symbol;Acc:HGNC:7105]	0.51	0.25	0.49	0.01	0.08	down
LAPTM5	lysosomal protein transmembrane 5 [Source:HGNC Symbol;Acc:HGNC:29612]	0.41	0.17	0.42	0.01	0.08	down
NPTN-IT1	NPTN intronic transcript 1 [Source:HGNC Symbol;Acc:HGNC:45091]	2.36	1.14	0.48	0.01	0.08	down
AC067956	novel transcript	0.53	0.20	0.38	0.01	0.08	down
PRR20G	proline rich 20G [Source:HGNC Symbol;Acc:HGNC:53837]	1.14	0.40	0.35	0.01	0.09	down
ZNF460	zinc finger protein 460 [Source:HGNC Symbol;Acc:HGNC:21628]	0.88	0.35	0.40	0.01	0.09	down
SULT1C2	sulfotransferase family 1C member 2 [Source:HGNC Symbol;Acc:HGNC:11456]	0.30	0.12	0.42	0.01	0.09	down
PRSS35	serine protease 35 [Source:HGNC Symbol;Acc:HGNC:21387]	0.52	0.21	0.40	0.01	0.09	down
AC092953	novel transcript, antisense to ATP11B	1.68	0.64	0.38	0.01	0.09	down
SNORD58A	small nucleolar RNA, C/D box 58A [Source:HGNC Symbol;Acc:HGNC:10208]	24.60	10.70	0.44	0.01	0.09	down
HS6ST3	heparan sulfate 6-O-sulfotransferase 3 [Source:HGNC Symbol;Acc:HGNC:19134]	0.46	0.23	0.50	0.01	0.09	down
AC008957	TEC	0.46	0.14	0.30	0.01	0.09	down
AC100821	ESF1, nucleolar pre-rRNA processing protein, homolog (S. cerevisiae) (ESF1) pseudogene	0.35	0.12	0.34	0.01	0.09	down
ORM1	orosomucoid 1 [Source:HGNC Symbol;Acc:HGNC:8498]	1.26	0.28	0.22	0.01	0.09	down
NOG	noggin [Source:HGNC Symbol;Acc:HGNC:7866]	1.56	0.70	0.45	0.01	0.09	down
DOCK11	dedicator of cytokinesis 11 [Source:HGNC Symbol;Acc:HGNC:23483]	0.46	0.19	0.42	0.01	0.09	down
HBA2	hemoglobin subunit alpha 2 [Source:HGNC Symbol;Acc:HGNC:4824]	2.92	1.33	0.46	0.01	0.09	down
AKAP2	A-kinase anchoring protein 2 [Source:HGNC Symbol;Acc:HGNC:372]	9.22	2.64	0.29	0.01	0.10	down
ATXN7	ataxin 7 [Source:HGNC Symbol;Acc:HGNC:10560]	0.33	0.05	0.16	0.01	0.10	down
AC078795	novel transcript, antisense to MYNN	1.00	0.48	0.47	0.01	0.10	down
ATP6V1C2	ATPase H+ transporting V1 subunit C2 [Source:HGNC Symbol;Acc:HGNC:18264]	1.66	0.80	0.48	0.01	0.10	down
KRT80	keratin 80 [Source:HGNC Symbol;Acc:HGNC:27056]	1.31	0.64	0.49	0.01	0.10	down
SPINK2	serine peptidase inhibitor, Kazal type 2 [Source:HGNC Symbol;Acc:HGNC:11245]	3.03	1.40	0.46	0.01	0.10	down
AC005089	novel transcript	1.18	0.41	0.35	0.01	0.10	down
TMEM202-AS1	TMEM202 antisense RNA 1 [Source:HGNC Symbol;Acc:HGNC:53265]	1.16	0.30	0.26	0.01	0.11	down
TTYH1	tweety family member 1 [Source:HGNC Symbol;Acc:HGNC:13476]	0.48	0.24	0.49	0.01	0.11	down
HLA-DPA1	major histocompatibility complex, class II, DP alpha 1 [Source:HGNC Symbol;Acc:HGNC:4938]	0.48	0.24	0.50	0.01	0.11	down
TCTEX1D4	Tctex1 domain containing 4 [Source:HGNC Symbol;Acc:HGNC:32315]	2.05	0.67	0.33	0.01	0.12	down
SNORD14D	small nucleolar RNA, C/D box 14D [Source:HGNC Symbol;Acc:HGNC:30353]	12.43	2.33	0.19	0.01	0.12	down
CLIC5	chloride intracellular channel 5 [Source:HGNC Symbol;Acc:HGNC:13517]	0.13	0.05	0.41	0.01	0.12	down
AC105052	novel protein, POLR2J2-UPK3BL readthrough	1.35	0.57	0.42	0.01	0.12	down
NOTCH2NLA	notch 2 N-terminal like A [Source:HGNC Symbol;Acc:HGNC:31862]	2.56	1.12	0.44	0.01	0.12	down
SLC25A48	solute carrier family 25 member 48 [Source:HGNC Symbol;Acc:HGNC:30451]	0.20	0.09	0.46	0.01	0.12	down
GFY	golgi associated olfactory signaling regulator [Source:HGNC Symbol;Acc:HGNC:44663]	1.93	0.89	0.46	0.01	0.13	down
FOLR1	folate receptor 1 [Source:HGNC Symbol;Acc:HGNC:3791]	0.78	0.26	0.33	0.01	0.13	down
JPT2	Jupiter microtubule associated homolog 2 [Source:HGNC Symbol;Acc:HGNC:14137]	10.41	4.89	0.47	0.02	0.13	down
AC000123	TEC	0.62	0.23	0.37	0.02	0.13	down
AK4P3	adenylate kinase 4 pseudogene 3 [Source:HGNC Symbol;Acc:HGNC:21596]	1.77	0.71	0.40	0.02	0.13	down
FO681492	synaptotagmin-15 [Source:NCBI gene;Acc:102724488]	0.23	0.10	0.45	0.02	0.13	down
SOX7	SRY-box 7 [Source:HGNC Symbol;Acc:HGNC:18196]	0.29	0.11	0.37	0.02	0.14	down
GCSAM	germinal center associated signaling and motility [Source:HGNC Symbol;Acc:HGNC:20253]	0.48	0.23	0.47	0.02	0.14	down
ZNF684	zinc finger protein 684 [Source:HGNC Symbol;Acc:HGNC:28418]	0.71	0.30	0.42	0.02	0.14	down
POMK	protein-O-mannose kinase [Source:HGNC Symbol;Acc:HGNC:26267]	3.90	1.77	0.45	0.02	0.14	down
AP002990	novel transcript	191.82	23.86	0.12	0.02	0.14	down
TATDN2P2	TatD DNase domain containing 2 pseudogene 2 [Source:HGNC Symbol;Acc:HGNC:39255]	0.50	0.21	0.42	0.02	0.14	down
OXCT2P1	3-oxoacid CoA-transferase 2 pseudogene 1 [Source:HGNC Symbol;Acc:HGNC:21627]	1.21	0.59	0.49	0.02	0.14	down
CFL1P5	cofilin 1 pseudogene 5 [Source:HGNC Symbol;Acc:HGNC:1877]	1.77	0.65	0.37	0.02	0.14	down
ACER3	alkaline ceramidase 3 [Source:HGNC Symbol;Acc:HGNC:16066]	3.23	1.61	0.50	0.02	0.15	down
AL731566	novel transcript, antisense to NSMCE4A	0.92	0.39	0.43	0.02	0.15	down
AL136084	novel transcript, antisense to GALNT12	0.30	0.12	0.40	0.02	0.15	down
HLA-DPB1	major histocompatibility complex, class II, DP beta 1 [Source:HGNC Symbol;Acc:HGNC:4940]	0.30	0.12	0.39	0.02	0.16	down
KRR1P1	KRR1, small subunit processome component homolog pseudogene 1 [Source:HGNC Symbol;Acc:H	1.33	0.56	0.42	0.02	0.16	down
BMS1P1	BMS1, ribosome biogenesis factor pseudogene 1 [Source:HGNC Symbol;Acc:HGNC:23649]	0.59	0.28	0.48	0.02	0.16	down
GRIK2	glutamate ionotropic receptor kainate type subunit 2 [Source:HGNC Symbol;Acc:HGNC:4580]	0.22	0.11	0.49	0.02	0.16	down
ANKRD20A2	ankyrin repeat domain 20 family member A2 [Source:HGNC Symbol;Acc:HGNC:31979]	0.26	0.10	0.39	0.02	0.16	down
LVRN	laeverin [Source:HGNC Symbol;Acc:HGNC:26904]	0.32	0.14	0.43	0.02	0.16	down
RBM44	RNA binding motif protein 44 [Source:HGNC Symbol;Acc:HGNC:24756]	0.22	0.10	0.46	0.02	0.16	down
REPS2	RALBP1 associated Eps domain containing 2 [Source:HGNC Symbol;Acc:HGNC:9963]	0.45	0.22	0.50	0.02	0.17	down
AC004918	novel transcript	1.72	0.79	0.46	0.02	0.17	down
AC012181	novel transcript, sense intronic to HERPUD1	4.78	2.35	0.49	0.02	0.17	down
AC067852	novel transcript	0.49	0.14	0.29	0.02	0.17	down
KRTAP2-5P	keratin associated protein 2-5, pseudogene [Source:HGNC Symbol;Acc:HGNC:18920]	2.45	0.91	0.37	0.03	0.18	down
AGPAT4-IT1	AGPAT4 intronic transcript 1 [Source:HGNC Symbol;Acc:HGNC:20988]	0.67	0.30	0.44	0.03	0.18	down
RPS26P6	ribosomal protein S26 pseudogene 6 [Source:HGNC Symbol;Acc:HGNC:31090]	33.08	13.85	0.42	0.03	0.18	down
USP32P3	ubiquitin specific peptidase 32 pseudogene 3 [Source:HGNC Symbol;Acc:HGNC:43576]	0.51	0.24	0.48	0.03	0.18	down
KCNK9	potassium two pore domain channel subfamily K member 9 [Source:HGNC Symbol;Acc:HGNC:62	0.16	0.07	0.45	0.03	0.18	down
AC018695	novel transcript, antisense to COX4NB	1.37	0.62	0.46	0.03	0.18	down
AC069544	novel transcript	1.23	0.53	0.43	0.03	0.18	down
MICE	MHC class I polypeptide-related sequence E (pseudogene) [Source:HGNC Symbol;Acc:HGNC:709	0.87	0.39	0.45	0.03	0.19	down
CMYA5	cardiomyopathy associated 5 [Source:HGNC Symbol;Acc:HGNC:14305]	0.10	0.05	0.45	0.03	0.19	down
AC005519	novel transcript, sense overlapping ABCD4	1.21	0.59	0.49	0.03	0.19	down
AC093495	novel transcript, antisense to XPC	0.22	0.10	0.45	0.03	0.19	down
AP001033	novel transcript, sense intronic to ANKRD12	1.86	0.87	0.47	0.03	0.19	down
AC025031	novel transcript	0.36	0.14	0.39	0.03	0.19	down
UBA7	ubiquitin like modifier activating enzyme 7 [Source:HGNC Symbol;Acc:HGNC:12471]	0.33	0.16	0.47	0.03	0.19	down
CSDC2	cold shock domain containing C2 [Source:HGNC Symbol;Acc:HGNC:30359]	0.26	0.11	0.43	0.03	0.20	down

AC115618	TEC	3.84	1.09	0.28	0.03	0.21	down
AC068587	RNA binding protein, fox-1 homolog (C. elegans) 1 (RBFOX1) pseudogene	4.83	1.50	0.31	0.03	0.21	down
MT-TG	mitochondrially encoded tRNA-Gly (GGN) [Source:HGNC Symbol;Acc:HGNC:7486]	83.84	33.59	0.40	0.03	0.21	down
AC106886	novel transcript	1.02	0.14	0.14	0.03	0.21	down
AL049844	synaptophysin-like 1 (SYPL1) pseudogene	1.23	0.51	0.42	0.03	0.21	down
AL359513	novel transcript	2.13	0.93	0.44	0.04	0.21	down
AC004264	novel transcript, antisense to LIF	1.85	0.88	0.47	0.04	0.22	down
AL669918	novel protein, TAP2-HLA-DOB readthrough	0.37	0.16	0.44	0.04	0.22	down
LINC02562	long intergenic non-protein coding RNA 2562 [Source:HGNC Symbol;Acc:HGNC:53602]	0.98	0.47	0.48	0.04	0.22	down
AC008115	novel transcript	1.81	0.77	0.43	0.04	0.22	down
SPDYE5	speedy/RINGO cell cycle regulator family member E5 [Source:HGNC Symbol;Acc:HGNC:35464]	0.24	0.07	0.31	0.04	0.23	down
CTAGE9	CTAGE family member 9 [Source:HGNC Symbol;Acc:HGNC:37275]	0.97	0.45	0.46	0.04	0.23	down
BTN1A1	butyrophilin subfamily 1 member A1 [Source:HGNC Symbol;Acc:HGNC:1135]	0.28	0.13	0.47	0.04	0.23	down
MT-TL2	mitochondrially encoded tRNA-Leu (CUN) 2 [Source:HGNC Symbol;Acc:HGNC:7491]	97.68	38.76	0.40	0.04	0.23	down
EMC3-AS1	EMC3 antisense RNA 1 [Source:HGNC Symbol;Acc:HGNC:49223]	0.46	0.19	0.42	0.04	0.24	down
AC091185	novel transcript	1.92	0.91	0.47	0.04	0.24	down
ZNF320	zinc finger protein 320 [Source:HGNC Symbol;Acc:HGNC:13842]	1.05	0.35	0.34	0.05	0.25	down
KCNAB1	potassium voltage-gated channel subfamily A member regulatory beta subunit 1 [Source:HGNC Syn	0.19	0.09	0.48	0.05	0.25	down
SLC6A6	solute carrier family 6 member 6 [Source:HGNC Symbol;Acc:HGNC:11052]	13.94	5.90	0.42	0.05	0.25	down
AC010531	novel protein	2.04	0.52	0.26	0.05	0.25	down
AL049697	novel transcript, C6orf165-SLC35A1 readthrough	0.42	0.16	0.38	0.05	0.25	down
AL031775	novel transcript, antisense to ACOT13	2.12	0.86	0.40	0.05	0.25	down
CNTNAP3C	contactin associated protein like 3C [Source:HGNC Symbol;Acc:HGNC:53878]	0.84	0.39	0.46	0.05	0.25	down
AC068580	novel transcript	0.49	0.23	0.48	0.05	0.26	down

Table S3 The 20 most enriched oncogenic signature gene sets (ten positive and ten negative each) in circHIPK2 knockdown cells according to GSEA analysis.

Signaling pathway	Log_{10}(*P*- value)	NES	Brief description
CORDENONSI_YAP_CONSERVED_SIGNATURE	-2.596597096	-2.487118833	YAP conserved signature.
BCAT_BILD_ET_AL_DN	-1.743733686	-2.042004458	Genes down-regulated in primary epithelial breast cancer cell culture over-expressing activated CTNNB1 [GeneID=1499] gene.
VEGF_A_UP.V1_DN	-2.18610838	-1.84679511	Genes down-regulated in HUVEC cells (endothelium) by treatment with VEGFA [GeneID=7422].
CSR_LATE_UP.V1_UP	-1.720159303	-1.699287584	Genes up-regulated in late serum response of CRL 2091 cells (foreskin fibroblasts).
ESC_V6.5_UP_LATE.V1_UP	-1.789580712	-1.684021918	Genes up-regulated during late stages of differentiation of embryoid bodies from V6.5 embryonic stem cells.
RB_P107_DN.V1_UP	-1.376015127	-1.681254463	Genes up-regulated in primary keratinocytes from RB1 and RBL1 [GeneID=5925] [GeneID=5933] skin specific knockout mice.
IL15_UP.V1_DN	-1.545444573	-1.674604921	Genes down-regulated in Sez-4 cells (T lymphocyte) that were first starved of IL2 [GeneID=3558] and then stimulated with IL15 [GeneID=3600].
RPS14_DN.V1_UP	-1.485721426	-1.656571131	Genes up-regulated in CD34+ hematopoietic progenitor cells after knockdown of RPS14 [GeneID=6208] by RNAi.
RPS14_DN.V1_DN	-1.489958479	-1.645875582	Genes down-regulated in CD34+ hematopoietic progenitor cells after knockdown of RPS14 [GeneID=6208] by RNAi.
P53_DN.V1_UP	-1.482873584	-1.590415279	Genes up-regulated in NCI-60 panel of cell lines with mutated TP53 [GeneID=7157].
SIRNA_EIF4GI_UP	2.525044807	1.691934636	Genes up-regulated in MCF10A cells vs knockdown of EIF4G1 [GeneID=1981] gene by RNAi.
ALK_DN.V1_UP	2.35666312	1.669994877	Genes up-regulated in DAOY cells (medulloblastoma) upon knockdown of ALK [GeneID=238] gene by RNAi.
IL2_UP.V1_UP	2.140508043	1.580049807	Genes up-regulated in Sez-4 cells (T lymphocyte) that were first starved of IL2 [GeneID=3558] and then stimulated with IL2 [GeneID=3558].
TBK1.DN.48HRS_DN	1.486430479	1.547411773	Genes down-regulated in epithelial lung cancer cell lines upon over-expression of an oncogenic form of KRAS [GeneID=3845] gene and knockdown of TBK1
NFE2L2.V2	2.586587305	1.532439115	Genes down-regulated in MEF cells (embryonic fibroblasts) after knockout of NFE2L2 [GeneID=4780] gene.
RB_P107_DN.V1_DN	1.687656339	1.519076994	Genes down-regulated in primary keratinocytes from RB1 and RBL1 [GeneID=5925] [GeneID=5933] skin specific knockout mice.
MTOR_UP.N4.V1_DN	2.060067972	1.50665087	Genes down-regulated in CEM-C1 cells (T-CLL) in comparison of control vs rapamycin (sirolimus) [PubChem=6610346], an mTOR pathway inhibitor.
ESC_J1_UP_EARLY.V1_DN	1.757775491	1.451447101	Genes down-regulated during early stages of differentiation of embryoid bodies from J1 embryonic stem cells.
MTOR_UP.N4.V1_UP	1.694605199	1.412269752	Genes up-regulated in CEM-C1 cells (T-CLL) in comparison of control vs rapamycin (sirolimus) [PubChem=6610346], an mTOR pathway inhibitor.
AKT_UP_MTOR_DN.V1_DN	1.696481204	1.404994533	Genes down-regulated by everolimus [PubChem=6442177] in mouse prostate tissue transgenically expressing human AKT1 gene [GeneID=207] vs untreated controls.

Table S4 The 59 enriched proteins pulled down by circHIPK2 in HCT116 cells.

Protein name	Description	Protein score	Protein coverage	EmPAI
ACOT9	Acyl-coenzyme A thioesterase 9, mitochondrial OS=Homo sapiens OX=9606 GN=ACOT9 PE=1 SV=2	451	27.8	1.76
ACTB	Actin, cytoplasmic 1 OS=Homo sapiens OX=9606 GN=ACTB PE=1 SV=1	381	29.3	1.88
ACTA2	Actin, aortic smooth muscle OS=Homo sapiens OX=9606 GN=ACTA2 PE=1 SV=1	214	22.5	1.12
TARBP2	RISC-loading complex subunit TARBP2 OS=Homo sapiens OX=9606 GN=TARBP2 PE=1 SV=3	190	12.6	0.38
ALDOA	Fructose-bisphosphate aldolase A OS=Homo sapiens OX=9606 GN=ALDOA PE=1 SV=2	129	7.4	0.27
CNN3	Calponin-3 OS=Homo sapiens OX=9606 GN=CNN3 PE=1 SV=1	113	10.3	0.3
GAPDH	Glyceraldehyde-3-phosphate dehydrogenase OS=Homo sapiens OX=9606 GN=GAPDH PE=1 SV=3	108	8.1	0.3
M6PR	Cation-dependent mannose-6-phosphate receptor OS=Homo sapiens OX=9606 GN=M6PR PE=1 SV=1	106	5.1	0.22
RAB1B	Ras-related protein Rab-1B OS=Homo sapiens OX=9606 GN=RAB1B PE=1 SV=1	102	18.9	0.52
RAB6A	Ras-related protein Rab-6A OS=Homo sapiens OX=9606 GN=RAB6A PE=1 SV=3	95	12	0.3
**EIF4A3**	**Eukaryotic initiation factor 4A-III OS=Homo sapiens OX=9606 GN=EIF4A3 PE=1 SV=4**	**90**	**8**	**0.22**
RPS13	40S ribosomal protein S13 OS=Homo sapiens OX=9606 GN=RPS13 PE=1 SV=2	89	7.9	0.43
APMAP	Adipocyte plasma membrane-associated protein OS=Homo sapiens OX=9606 GN=APMAP PE=1 SV=2	87	4.6	0.15
PFN2	Profilin-2 OS=Homo sapiens OX=9606 GN=PFN2 PE=1 SV=3	86	10	0.22
TPI1	Triosephosphate isomerase OS=Homo sapiens OX=9606 GN=TPI1 PE=1 SV=4	84	5.6	0.26
IGKV2-28	Immunoglobulin kappa variable 2-28 OS=Homo sapiens OX=9606 GN=IGKV2-28 PE=3 SV=1	80	10.8	0.26
VAMP8	Vesicle-associated membrane protein 8 OS=Homo sapiens OX=9606 GN=VAMP8 PE=1 SV=1	80	24	0.69
DCD	Dermcidin OS=Homo sapiens OX=9606 GN=DCD PE=1 SV=2	79	12.7	0.69
PKM	Pyruvate kinase PKM OS=Homo sapiens OX=9606 GN=PKM PE=1 SV=4	72	5.1	0.12
TFRC	Transferrin receptor protein 1 OS=Homo sapiens OX=9606 GN=TFRC PE=1 SV=2	65	1.3	0.04
RAB4B	Ras-related protein Rab-4B OS=Homo sapiens OX=9606 GN=RAB4B PE=1 SV=1	62	11.3	0.3
HSPE1	10 kDa heat shock protein, mitochondrial OS=Homo sapiens OX=9606 GN=HSPE1 PE=1 SV=2	62	13.7	0.32
RPS20	40S ribosomal protein S20 OS=Homo sapiens OX=9606 GN=RPS20 PE=1 SV=1	60	9.2	0.25
SLC3A2	4F2 cell-surface antigen heavy chain OS=Homo sapiens OX=9606 GN=SLC3A2 PE=1 SV=3	59	2.4	0.05
PPIA	Peptidyl-prolyl cis-trans isomerase A OS=Homo sapiens OX=9606 GN=PPIA PE=1 SV=2	59	17	0.67
CHTOP	Chromatin target of PRMT1 protein OS=Homo sapiens OX=9606 GN=CHTOP PE=1 SV=2	58	6.5	0.43
CFL2	Cofilin-2 OS=Homo sapiens OX=9606 GN=CFL2 PE=1 SV=1	54	6.6	0.18
AHCY	Adenosylhomocysteinase OS=Homo sapiens OX=9606 GN=AHCY PE=1 SV=4	53	3	0.07
H4C1	Histone H4 OS=Homo sapiens OX=9606 GN=H4C1 PE=1 SV=2	53	9.7	0.3
RPS3	40S ribosomal protein S3 OS=Homo sapiens OX=9606 GN=RPS3 PE=1 SV=2	50	10.7	0.26
RPL23A	60S ribosomal protein L23a OS=Homo sapiens OX=9606 GN=RPL23A PE=1 SV=1	47	8.3	0.19
NUDT21	Cleavage and polyadenylation specificity factor subunit 5 OS=Homo sapiens OX=9606 GN=NUDT21 PE=1 SV=1	46	8.8	0.27
TMED10	Transmembrane emp24 domain-containing protein 10 OS=Homo sapiens OX=9606 GN=TMED10 PE=1 SV=2	45	11.9	0.28
PP1G	Serine/threonine-protein phosphatase PP1-gamma catalytic subunit OS=Homo sapiens OX=9606 GN=PPP1CC PE=[illegible]	44	3.1	0.09
CYB5R3	NADH-cytochrome b5 reductase 3 OS=Homo sapiens OX=9606 GN=CYB5R3 PE=1 SV=3	43	4	0.1
ENO1	Alpha-enolase OS=Homo sapiens OX=9606 GN=ENO1 PE=1 SV=2	43	2.3	0.07
TMED9	Transmembrane emp24 domain-containing protein 9 OS=Homo sapiens OX=9606 GN=TMED9 PE=1 SV=2	39	3.8	0.12
DCLK3	Serine/threonine-protein kinase DCLK3 OS=Homo sapiens OX=9606 GN=DCLK3 PE=2 SV=2	36	1.2	0.04
CNOT2	CCR4-NOT transcription complex subunit 2 OS=Homo sapiens OX=9606 GN=CNOT2 PE=1 SV=1	36	1.7	0.05
MYL6B	Myosin light chain 6B OS=Homo sapiens OX=9606 GN=MYL6B PE=1 SV=1	36	6.3	0.15
PLP2	Proteolipid protein 2 OS=Homo sapiens OX=9606 GN=PLP2 PE=1 SV=1	36	8.6	0.2
PPL34	60S ribosomal protein L34 OS=Homo sapiens OX=9606 GN=RPL34 PE=1 SV=3	35	6.8	0.25
ALDOC	Fructose-bisphosphate aldolase C OS=Homo sapiens OX=9606 GN=ALDOC PE=1 SV=2	33	2.2	0.08
ERH	Enhancer of rudimentary homolog OS=Homo sapiens OX=9606 GN=ERH PE=1 SV=1	33	14.4	0.28
RPS4Y1	40S ribosomal protein S4, Y isoform 1 OS=Homo sapiens OX=9606 GN=RPS4Y1 PE=1 SV=2	33	4.2	0.11
RPS15	40S ribosomal protein S15 OS=Homo sapiens OX=9606 GN=RPS15 PE=1 SV=2	32	8.3	0.2
RPS2	40S ribosomal protein S2 OS=Homo sapiens OX=9606 GN=RPS2 PE=1 SV=2	30	3.8	0.11
RPS9	40S ribosomal protein S9 OS=Homo sapiens OX=9606 GN=RPS9 PE=1 SV=3	28	5.7	0.15
MUC5B	Mucin-5B OS=Homo sapiens OX=9606 GN=MUC5B PE=1 SV=3	28	0.2	0.01
FBL	rRNA 2~-O-methyltransferase fibrillarin OS=Homo sapiens OX=9606 GN=FBL PE=1 SV=2	28	3.7	0.1
APAF1	Apoptotic protease-activating factor 1 OS=Homo sapiens OX=9606 GN=APAF1 PE=1 SV=2	25	1.1	0.02
N6AMT1	Methyltransferase N6AMT1 OS=Homo sapiens OX=9606 GN=N6AMT1 PE=1 SV=4	25	4.2	0.14
RBM10	RNA-binding protein 10 OS=Homo sapiens OX=9606 GN=RBM10 PE=1 SV=3	24	1.5	0.03
NMS	Neuromedin-S OS=Homo sapiens OX=9606 GN=NMS PE=2 SV=1	24	9.8	0.19
SEC61B	Protein transport protein Sec61 subunit beta OS=Homo sapiens OX=9606 GN=SEC61B PE=1 SV=2	24	10.4	0.35
ARL16	ADP-ribosylation factor-like protein 16 OS=Homo sapiens OX=9606 GN=ARL16 PE=1 SV=1	21	6.6	0.16
UGT1A7	UDP-glucuronosyltransferase 1A7 OS=Homo sapiens OX=9606 GN=UGT1A7 PE=1 SV=2	18	1.3	0.05
CNST	Consortin OS=Homo sapiens OX=9606 GN=CNST PE=1 SV=3	15	1.5	0.04
KIF4B	Chromosome-associated kinesin KIF4B OS=Homo sapiens OX=9606 GN=KIF4B PE=2 SV=2	14	0.9	0.02

Table S5 The 22 significantly downregulated genes of YAP conserved signature upon circHIPK2

Gene name	Fold change (sicircHIPK2/siNC)	*P* value
ITGB5	0.78797985	0.031862361
THBS1	0.62721031	0.032156513
FLNA	0.757407732	0.00964431
ASAP1	0.746867337	0.007324769
PDLIM2	0.710893933	0.02242059
TOP2A	0.725957178	0.004461819
HMMR	0.713418617	0.027779319
TK1	0.710462561	0.003421415
CDC20	0.704622074	0.00151803
STMN1	0.693047897	0.002561475
AXL	0.689341455	0.001265939
ETV5	0.684212917	0.000761244
ECT2	0.680609596	0.000596476
GGH	0.666985927	0.000357567
SHCBP1	0.650638133	0.001135049
CRIM1	0.585984191	1.85318E-06
AMOTL2	0.5589943	9.67123E-06
SLIT2	0.516645557	0.001514753
CENPF	0.527298091	1.25574E-07
TGM2	0.51494916	3.24199E-09
**CCN2**	**0.390052056**	**1.17809E-06**
**CCN1**	**0.367786285**	**3.52053E-13**

Table S6 The predicted RBPs binding to flanking regions of circHIPK2 pre-mRNA.

circAtlas	RBPmap	Ref.16
DDX3X	A1CF	MBL
DDX3	ANKHD1	RBM20
**FUS**	BOLL	QKI
UPF1	BRUNOL4	HNRNPL
XPO5	BRUNOL5	**FUS**
HNRNPC	BRUNOL6	ILF3
CSTF2T	CELF1	DHX9
CELF2	CNOT4	ADAR1
YTHDC1	CPEB1	
CSTF2	CPEB2	
HNRNPA1	CPEB4	
TARDBP	DAZ3	
HNRNPH	DAZAP1	
WDR43	EIF4G2	
PTBP1	ELAVL4	
CDC40	ENOX1	
NUDT21	ESRP1	
RBM15	ESRP2	
TAF15	EWSR1	
DGCR8	FMR1	
UCHL5	FUBP1	
PCBP1	FUBP3	
SF3B1	**FUS**	
FIP1L1	FXR1	
CPSF6	FXR2	
WDR33	G3BP2	
RBM15B	HNRNPA0	
	HNRNPA1	
	HNRNPA1L2	
	HNRNPA2B1	
	HNRNPC	
	HNRNPCL1	
	HNRNPD	
	HNRNPDL	
	HNRNPF	
	HNRNPH1	
	HNRNPH2	
	HNRNPK	
	HNRNPL	
	HNRNPM	
	HNRNPU	
	HNRPLL	
	HuR	
	IGF2BP1	
	IGF2BP2	
	IGF2BP3	
	ILF2	
	KHDRBS1	
	KHDRBS2	
	KHDRBS3	
	KHSRP	
	LIN28A	
	MATR3	
	MBNL1	
	MSI1	
	NOVA1	
	NUPL2	
	PABPC1	
	PABPC3	
	PABPC4	
	PABPC5	
	PABPN1	
	PABPN1L	
	PCBP1	
	PCBP2	
	PCBP3	
	PCBP4	
	PRR3	
	PTB3	
	PTBP3	
	PUF60	
	PUM1	
	PUM2	
	QKI	
	RALY	

RBFOX1
RBFOX2
RBFOX3
RBM15B
RBM22
RBM23
RBM24
RBM25
RBM28
RBM3
RBM38
RBM4
RBM41
RBM42
RBM45
RBM46
RBM47
RBM4B
RBM5
RBM6
RBM8A
RBMS1
RBMS2
RBMS3
RC3H1
SAMD4A
SART3
SF1
SFPQ
SNRNP70
SNRPA
SRSF1
SRSF10
SRSF11
SRSF2
SRSF4
SRSF5
SRSF7
SRSF8
SRSF9
TAF15
TARDBP
TIA1
TRA2A
TRNAU1AP
TUT1
U2AF2
UNK
YBX1
YBX2
ZC3H14
ZCRB1
ZFP36
ZNF326
ZNF638

Table S7 Oligonucleotides used for RT-qPCR, siRNA, and plasmid construction.

Name	Forward	Reverse
**Oligonucleotides used for real-time RT-PCR**		
circHIPK2	5' CTGTGTGCTCCACCTACTTG 3'	5' TACCCAGTCATGTCCCAGTTG 3'
GAPDH	5' ACCCACTCCTCCACCTTTG 3'	5' CACCACCCTGTTGCTGTAG 3'
NEAT1	5' GCTGGACCTTTCATGTAACGGG 3'	5' TGAACTCTGCCGGTACAGGGAA 3'
HIPK2	5' ACACAGGCTCAAGATGGCAG 3'	5' TTCGTACACGGGGGCCAT 3'
YAP	5' TGTCCCAGATGAACGTCACAGC 3'	5' TGGTGGCTGTTTCACTGGAGCA 3'
TAZ	5' GAGGACTTCCTCAGCAATGTGG 3'	5' CGTTTGTTCCTGGAAGACAGTCA 3'
EIF4A3	5' GGCACAGGAAAAACAGCCACCT 3'	5' TGTAGTCACCGAGAGCAAGCAG 3'
CCN1	5' GGAAAAGGCAGCTCACTGAAGC 3'	5' GGAGATACCAGTTCCACAGGTC 3'
CCN2	5' CTTGCGAAGCTGACCTGGAAGA 3'	5' CCGTCGGTACATACTCCACAGA 3'
CCN1-intron	5' CAACGAAAGCGCATACGGAA 3'	5' ATTCCAAACCACCGCCTGAA 3'
CCN1-exon-intron	5' CGAGGACTGCAGCAAAACG 3'	5' ACTTCTGGACTATGGGGACT 3'
CCN2-intron	5' TCCAAGAGAGGCTCTGGCTAT 3'	5' AGGAGTGGCTTTCTTCAACCC 3'
CCN2-exon-intron	5' ACCAATGACAACGCCTCCTG 3'	5' AGAGCCCTAAGTTGGGTCCTA 3'
ACTIN	5' GAGCTACGAGCTGCCTGACG 3'	5' GTAGTTTCGTGGATGCCACAG 3'
QKI	5' AATCCTTGAGTATCCTATTGAACCTAGT 3'	5' GCATATCGTGCCTTCGAACTTT 3'
FUS	5' CAAGGCCTGGGTGAGAATGT 3'	5' TTGCCTCTCCCTTCAGCTTG 3'
HNRNPL	5' GTGTGGTGGAAGCAGACCTTGT 3'	5' CAAACTCCACCAGTGCTTGTCTC 3'
a	5' CGAGTTGGGGGAGATGGATTA 3'	5' GAGTAACAGCCGCATTCCCC 3'
c	5' CAGGGATGGAAAGCAAGACC 3'	5' CTGCACTGTTCGGCACCA 3'
d	5' CCGGAGGCCATCCTGCTT 3'	5' CCTTGAAAGGCGCATGGAGA 3'
e	5' CTGAGGATTCCTTTCCCGCTC 3'	5' ACAGAGTGGATGGCCTGGTC 3'
intron 14	5' GAGAATCACTGCACGGGGAA 3'	5' TCACGTTGCTGGAGGACTTG 3'
m-circHipk2	5' TCCAAAGCTGTCTGCTCTACG 3'	5' AGGGTGTGAGGGGAGAAAAC 3'
m-Taz	5' CCTTATCACCGTCTCCAACCAC 3'	5' CCTTGGTGAAGCAGATGTCTGC 3'
m-Il10	5' GGTTGCCAAGCCTTATCGGA 3'	5' ACCTGCTCCACTGCCTTGCT 3'
m-Tgfβ	5' TGACGTCACTGGAGTTGTACGG 3'	5' GGTTCATGTCATGGATGGTGC 3'
m-Il6	5' TACCACTTCACAAGTCGGAGGC 3'	5' CTGCAAGTGCATCATCGTTGTTC 3'
m-Il17	5'CAGACTACCTCAACCGTTCCAC 3'	5' TCCAGCTTTCCCTCCGCATTGA 3'
m-Il22	5' GCTTGAGGTGTCCAACTTCCAG 3'	5' ACTCCTCGGAACAGTTTCTCCC 3'
m-Ccn1	5' GGAGGTGGAGTTAACGAGAAAC 3'	5' GTGGTCTGAACGATGCATTTC 3'
m-Ccn2	5' TGCGAAGCTGACCTGGAGGAAA 3'	5' CCGCAGAACTTAGCCCTGTATG 3'
m-Actin	5' CATTGCTGACAGGATGCAGAAGG 3'	5' TGCTGGAAGGTGGACAGTGAGG 3'
**Oligonucleotides used for siRNA**		
sicircHIPK2	5' CCAGAUAUUACAGGUAUGG 3'	5' CCAUACCUGUAAUAUCUGG 3'
sicircHIPK2-2/sicircHipk2	5' GUAUGGCCUCACAUGUGCA 3'	5' UGCACAUGUGAGGCCAUAC 3'
sicircHipk2-2	5' UGCAAUCCAGAUACUACCG 3'	5' CGGUAGUAUCUGGAUUGCA 3'
siEIF4A3	5' GAGCAGAUUUACGAUGUAUTT 3'	5' AUACAUCGUAAAUCUGCUCTT 3'
siEIF4A3-2	5' CAAUCAAGCAGAUCAUCAA 3'	5' UUGAUGAUCUGCUUGAUUG 3'
siFUS	5' AAUAAUACCAAUCUGCUUG 3'	5' CAAGCAGAUUGGUAUUAUU 3'
siFUS-2	5' GGACAGCAGCAAAGCUAUA 3'	5' UAUAGCUUUGCUGCUGUCC 3'
siQKI	5' CUAUUAACCCACAGCAUUA 3'	5' UAAUGCUGUGGGUUAAUAG 3'
siHNRNPL	5' GACGGGTCTTGCAGTTACA 3'	5' UGUAACUGCAAGACCCGUC 3'
siNC	5‘ UUCUCCGAACGUGUCACGUTT 3’	5‘ ACGUGACACGUUCGGAGAATT 3’
**Oligonucleotides used for plasmid construction**		
shcircHIPK2	5' CCGGAACCAGATATTACAGGTATGGCTCGAGCCATACCTGTAATATCTGGTTTTT[illegible]	5' AATTCAAAAAAACCAGATATTACAGGTATGGCTCGAGCCATACCTGTAATATCTGGTT 3'
shcircHipk2	5' CCGGTACCGGTATGGCCTCACATTTCTCGAGAAATGTGAGGCCATACCGGTATTT[illegible]	5' AATTCAAAAATACCGGTATGGCCTCACATTTCTCGAGAAATGTGAGGCCATACCGGTA 3'
pCCN1	5' TACCGCTAGCCTCGAGATCCCACTCCCTCTAGCAAATG 3'	5' GCGTGAGCTCCTCGAGTGGTGTGCAGCGGACAG 3'
pCCN2	5' TACCGCTAGCCTCGAGTGAGGAATGCTTGGAAAGGA 3'	5' GCGTGAGCTCCTCGAGGAGCTGAATGGAGTCCTACAC 3'

Table S8 Clinical information of patients with IBD and healthy donor.

No.	Gender	Age	Diagnosis	Tissue
Healthy 1	Female	58	Healthy	Rectum
Healthy 2	Female	58	Healthy	Rectum
Healthy 3	Female	49	Healthy	Rectum
Healthy 4	Female	21	Healthy	Rectum
Healthy 5	Male	32	Healthy	Rectum
Healthy 6	Male	46	Healthy	Rectum
Healthy 7	Male	34	Healthy	Rectum
Healthy 8	Male	43	Healthy	Rectum
Healthy 9	Male	39	Healthy	Rectum
Patient 1	Male	23	CD	Sigmoid colon
Patient 2	Male	24	CD	Transverse colon
Patient 3	Male	34	CD	Terminal ileum
Patient 4	Male	25	CD	Ascending colon
Patient 5	Male	34	CD	Sigmoid colon
Patient 6	Male	40	CD	Terminal ileum
Patient 7	Male	19	CD	Ascending colon
Patient 8	Male	33	CD	Terminal ileum
Patient 9	Male	18	CD	Rectum
Patient 10	Male	20	CD	Ascending colon
Patient 11	Male	29	UC	Sigmoid colon
Patient 12	Female	24	UC	Sigmoid colon
Patient 13	Male	60	UC	Rectum
Patient 14	Female	29	UC	Rectum
Patient 15	Male	41	UC	Rectum
Patient 16	Female	29	UC	Descending colon
Patient 17	Female	16	UC	Rectum
Patient 18	Female	45	UC	Rectum
Patient 19	Male	31	UC	Sigmoid colon
Patient 20	Male	68	UC	Rectum
Patient 21	Male	44	UC	Rectum
Patient 22	Male	34	UC	Rectum
Patient 23	Female	55	UC	Sigmoid colon
Patient 24	Male	58	UC	Ascending colon
Patient 25	Female	27	UC	Rectum
Patient 26	Male	36	UC	Rectum
Patient 27	Male	51	UC	Sigmoid colon
Patient 28	Male	29	UC	Rectum
